# New Insights into the Binding Features of F508del CFTR Potentiators: A Molecular Docking, Pharmacophore Mapping and QSAR Analysis Approach

**DOI:** 10.3390/ph13120445

**Published:** 2020-12-04

**Authors:** Giada Righetti, Monica Casale, Michele Tonelli, Nara Liessi, Paola Fossa, Nicoletta Pedemonte, Enrico Millo, Elena Cichero

**Affiliations:** 1 Department of Pharmacy, Section of Medicinal Chemistry, School of Medical and Pharmaceutical Sciences, University of Genoa, Viale Benedetto XV 3, 16132 Genoa, Italy; righetti@difar.unige.it (G.R.); tonelli@difar.unige.it (M.T.); 2 Department of Pharmacy, Section of Chemistry and Food and Pharmaceutical Technologies, University of Genoa, Viale Cembrano 4, 16148 Genoa, Italy; casale@difar.unige.it; 3 Center of Excellence for Biomedical Research (CEBR), University of Genoa, Viale Benedetto XV 9, 16132 Genoa, Italy; liessi_nara@libero.it (N.L.); enrico.millo@unige.it (E.M.); 4 Department of Experimental Medicine, Section of Biochemistry, University of Genoa, Viale Benedetto XV 1, 16132 Genoa, Italy; 5 UOC Genetica Medica, IRCCS Istituto Giannina Gaslini, 16147 Genova, Italy; nicoletta.pedemonte@unige.it

**Keywords:** CFTR potentiators, F508del CFTR, CFTR modulator, VX-770, molecular docking, pharmacophore analyses

## Abstract

Cystic fibrosis (CF) is the autosomal recessive disorder most recurrent in Caucasian populations. To combat this disease, many life-prolonging therapies are required and deeply investigated, including the development of the so-called cystic fibrosis transmembrane conductance regulator (CFTR) modulators, such as correctors and potentiators. Combination therapy with the two series of drugs led to the approval of several multi-drug effective treatments, such as Orkambi, and to the recent promising evaluation of the triple-combination Elexacaftor-Tezacaftor-Ivacaftor. This scenario enlightened the effectiveness of the multi-drug approach to pave the way for the discovery of novel therapeutic agents to contrast CF. The recent X-crystallographic data about the human CFTR in complex with the well-known potentiator Ivacaftor (VX-770) opened the possibility to apply a computational study aimed to explore the key features involved in the potentiator binding. Herein, we discussed molecular docking studies performed onto the chemotypes so far discussed in the literature as CFTR potentiator, reporting the most relevant interactions responsible for their mechanism of action, involving Van der Waals interactions and π–π stacking with F236, Y304, F305 and F312, as well as H-bonding F931, Y304, S308 and R933. This kind of positioning will stabilize the effective potentiator at the CFTR channel. These data have been accompanied by pharmacophore analyses, which promoted the design of novel derivatives endowed with a main (hetero)aromatic core connected to proper substituents, featuring H-bonding moieties. A highly predictive quantitative-structure activity relationship (QSAR) model has been developed, giving a cross-validated r^2^ (r^2^_cv_) = 0.74, a non-cross validated r^2^ (r^2^_ncv_) = 0.90, root mean square error (RMSE) = 0.347, and a test set r^2^ (r^2^_pred_) = 0.86. On the whole, the results are expected to gain useful information to guide the further development and optimization of new CFTR potentiators.

## 1. Introduction

Cystic fibrosis (CF) is an autosomal recessive genetic disorder that results from the functional deficiency in a plasma membrane anion channel known as the cystic fibrosis transmembrane conductance regulator (CFTR) [1,2,3]. Cystic fibrosis transmembrane conductance regulator (CFTR) is a glycoprotein, which is encoded by the CFTR gene, and consists of five domains: two transmembrane domains (MSD1 and MSD2), two nucleotide binding domains (NBD1 and NBD2), and a regulatory R region. CFTR is a member of the ABC transporter ATPase family and allows for the transmembrane chloride ions to flow down the electrochemical gradient [4,5]. Defective function of this channel can be caused by a variety of mutations, grouped in six classes according to the mechanism through which they cause loss of function [6]. In general, class II mutations cause defects resulting in the improper folding (misfolding) of the protein and consequently, in its retention in endoplasmic reticulum and its subsequent degradation before reaching the plasma membrane of epithelial cells.

Conversely, mutations belonging to class III impair CFTR function and are involved in a channel gating defect. The most common mutation related to CF is the deletion of phenylalanine 508 (F508del) leading to F508del-CFTR, that presents both maturation (class II) and gating (class III) defects. The most frequent class III mutation is G551D [7,8]. 

Up to now, the search for drugs to be used for cystic fibrosis therapy was focused on the design and development of small molecules, namely CFTR modulators [9]. They were classified into five main groups, including read-through agents, correctors, potentiators, stabilizers and amplifiers [10], according to their different ability to target specific defects relative to a CFTR gene mutation. In particular, the main efforts revolved around the discovery of compounds that correct at least CFTR misfolding and ER retention as well as defective channel gating, which represent the two major contributors to CF [11]. 

It is believed that the folding and the channel activity defects of F508del can be addressed by means of modulators known as correctors (e.g., Lumacaftor; VX-809) [12] and potentiators (e.g., Ivacaftor; VX-770) [13,14,15], respectively. Correctors improve the trafficking of mutant CFTR to the plasma membrane, instead, potentiators should bind to F508del CFTR at the cell surface and increase chloride channel gating. 

VX-809 or VX-770 monotherapy did not lead to clinical benefit for CF patients with the F508del mutation [16,17], while their combination regimen provided a positive outcome, stabilizing the disease progression [18]. Accordingly, the combination therapy lumacaftor-ivacaftor reached the market, while other triple-combinations are under evaluation [19,20]. 

Along with this, recent experimental data released on the protein data bank (PDB code: 6O2P) [21] reported the detailed binding mode of VX-770 within the CFTR channel. In particular, the Authors reported a cryo-electron microscopy structure of human CFTR in complex with the U.S. Food and Drug Administration (FDA)-approved drug ivacaftor (VX-770) at 3.3-angstrom resolution.

This information highlighted a cavity within the transmembrane region of the channel as the main binding site for potentiators, focusing especially on aromatic residues, such as F312, F931 and F932 as key anchoring points at the protein, by means especially of Van der Waals contacts and π–π stackings. Indeed, Liu and co-workers reported two aromatic interactions and six hydrophobic contacts with the biological target, while only two H-bonds with F931 and S308 were pointed out. Conceivably, these data represent preliminary useful information to apply a thorough structure-based analysis of the most relevant amino-acids and key interactions involved in the protein-targeting, as well as to explore the pharmacophore features experienced not only by VX-770 but also by the most promising potentiators so far described in the literature. 

These calculations have been accompanied by quantitative structure–activity relationship (QSAR) analysis, to point out a number of chemical descriptors turning in the different potentiator ability of the collected derivatives. This kind of information has been used herein to apply combined structure-based and ligand-based approaches to explore in detail the putative molecular interactions of the aforementioned compounds.

Accordingly, in the search for new drugs for CF treatment, we recently reported the rational design, chemical synthesis and biochemical characterization of aminoarylthiazole derivatives (AAT) exhibiting interesting properties as F508del CFTR modulators [22,23,24,25]. 

These kind of molecules are quite interesting, behaving as dual acting compounds (corrector and potentiator) [23,24] or only potentiator depending on the type of substitution on the thiazole ring [22].

**Figure 1:**
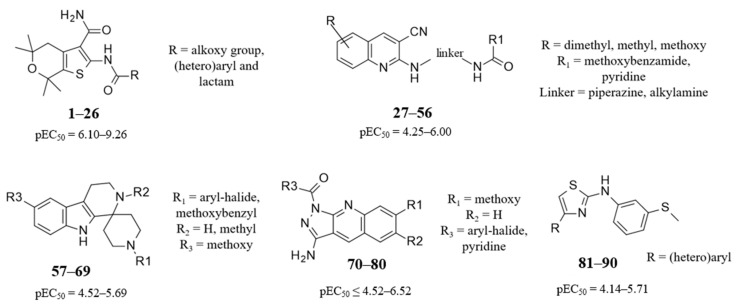
Chemical structure and biological activity of the selected series of F508del-CFTR potentiators. Potency of the compounds has been described as the negative logarithm of the half maximal effective concentration (pEC_50_), reported as molar unit.

Herein, we deem it interesting to collect different compounds’ library from the literature based on indole-containing compounds [26], pyrazolquinoline [26], thienopyrane [27], cyanoquinoline [28,29] and AAT [22,23] scaffolds, which showed a variable CFTR potentiator profile, in order to perform deepening molecular docking studies, pharmacophore analyses and QSAR studies (Figure 1).

This strategy allowed us to gain useful information, which is expected to pave the way for further rational design of novel derivatives and virtual screening strategy, speeding up the drug discovery processes towards improved and druggable CFTR modulators. 

## 2. Results 

### 2.1. Molecular Docking Studies

In this work, we proceeded with deepening molecular docking studies of different series of F508del CFTR potentiators, featuring variable chemo-types as well as potency trend, with the aim at exploring the most relevant key features turning in more effective potentiators (Supplementary Tables S1–S6). For all of them, the related potentiator activity has been assessed based on the iodide influx measured in the F508del CFTR expressing Fisher rat thyroid (FRT) cells, after low-temperature rescue in the presence of forskolin. While a number of cyanoquinolines experienced potentiator activity comparable with that of genistein, several piperidinepyridoindole and pyrazoloquinoline were endowed of 6-fold greater ability than that produced by VX-770 alone based on increased short-circuit current in primary human bronchial epithelial cells [26].

We deemed it interesting to take into account the X-ray crystallographic structure of VX-770 in complex with CFTR (PDB code = 6O2P, resolution = 3.30 Å) [21] as well as of GLPG1837 (PDB code = 6O1V, resolution = 3.20 Å) [21], being the thienopyrane analogues included in the collected dataset.

In particular, VX-770 was highly investigated in the literature, increasing the open probability (Po) of both wild-type (*wt*) and mutant CFTRs in membrane patches, proteo-liposomes and planar lipid bilayers. Since the potentiation by VX-770 requires phosphorylation of CFTR by protein kinase A (PKA) being independent of ATP, it is thought that this potentiator could directly target CFTR. Recently, the new potentiator GLPG1837 has been discovered, exhibiting higher efficacy than VX-770. Indeed, at saturating ATP concentration, the Po of the phosphorylated *wt* CFTR increased from 0.23 to 0.54 upon addition of 10 μM GLPG1837 [21].

**Figure 2:**
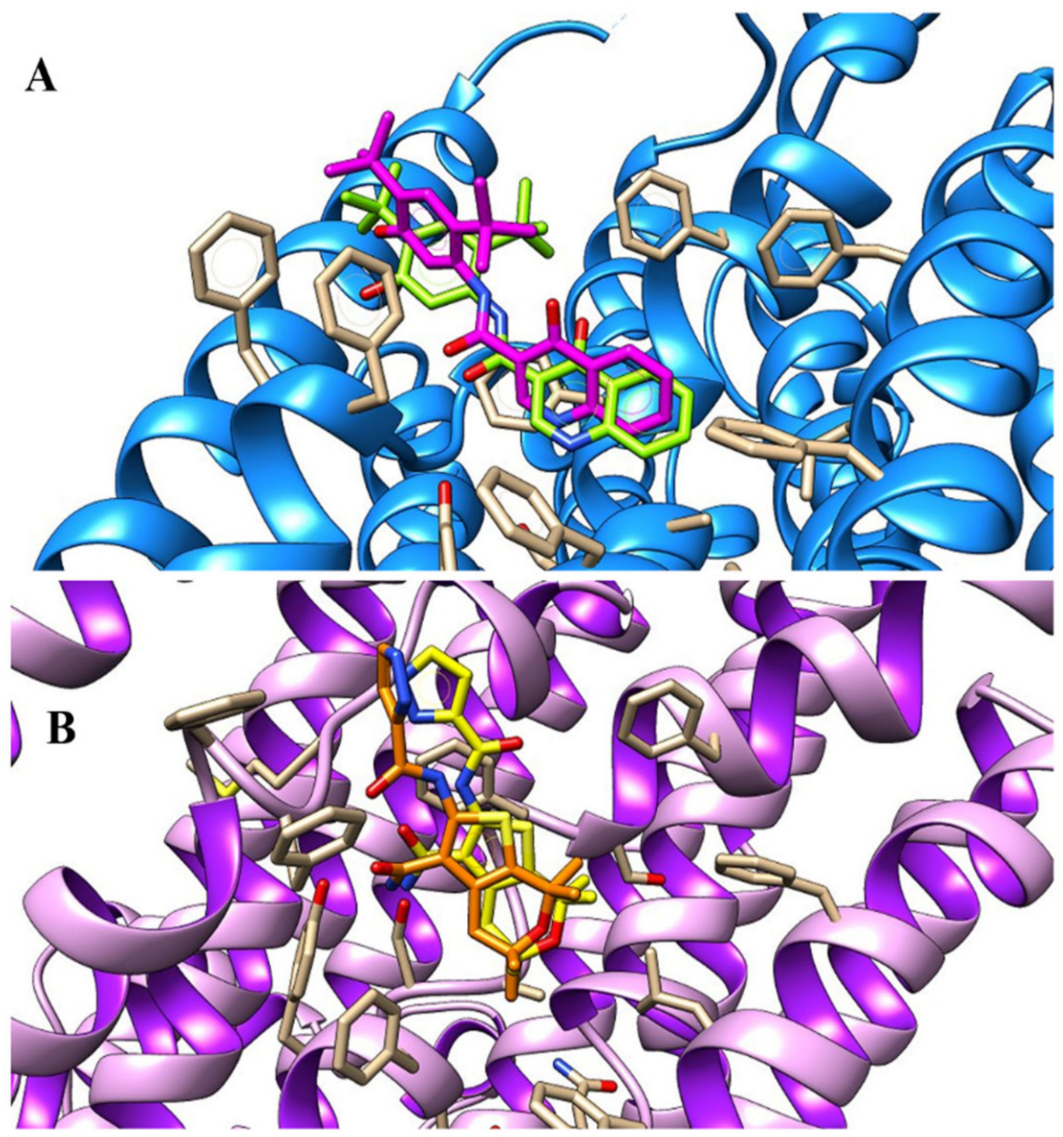
Docking positioning of VX-770 (*C* atom; magenta) (**A**) and of GLPG1837 (*C* atom; orange) (**B**) within the X-ray crystallographic data 6O2P and 6O1V, respectively. The co-crystallized ligands are shown in green and yellow [21].

Consequently, we proceeded with performing re-docking of the co-crystallized ligands in order to evaluate the reliability of the molecular docking protocol, to be applied in the following modeling studies of the potentiator series. As shown in Figure 2, both the two potentiators proved to be properly predicted within the CFTR channel, with the selected docking poses being in good agreement with the experimental data.

Comparing the two proteins, VX-770 and GLPG1837 occupied the same channel crevice, with the backbone of CFTR being highly overlapped (RMSD = 0.182 Å) (see Supplementary Figure S7). In any case, both the two potentiators were engaged in π–π stacking with F931 and F312 thanks to the main bicyclic core and the hydrophobic substituents, while the carboxamide moiety of GLPG1837 was H-bonded to Y304 and S308.

The following computational studies have been performed taking into account the experimental data concerning VX-770, chosen as a reference potentiator, being one of the CFTR modulators exploited in therapy (the related X-ray crystallographic positioning was shown in Supplementary Figure S7).

In particular, the derived CFTR-VX-770 complex was refined by energy minimization within the ‘Optimized Potentials for Liquid Simulations’-based force field (OPLS) by the Molecular Operating environment (MOE) software [30].

**Figure 3:**
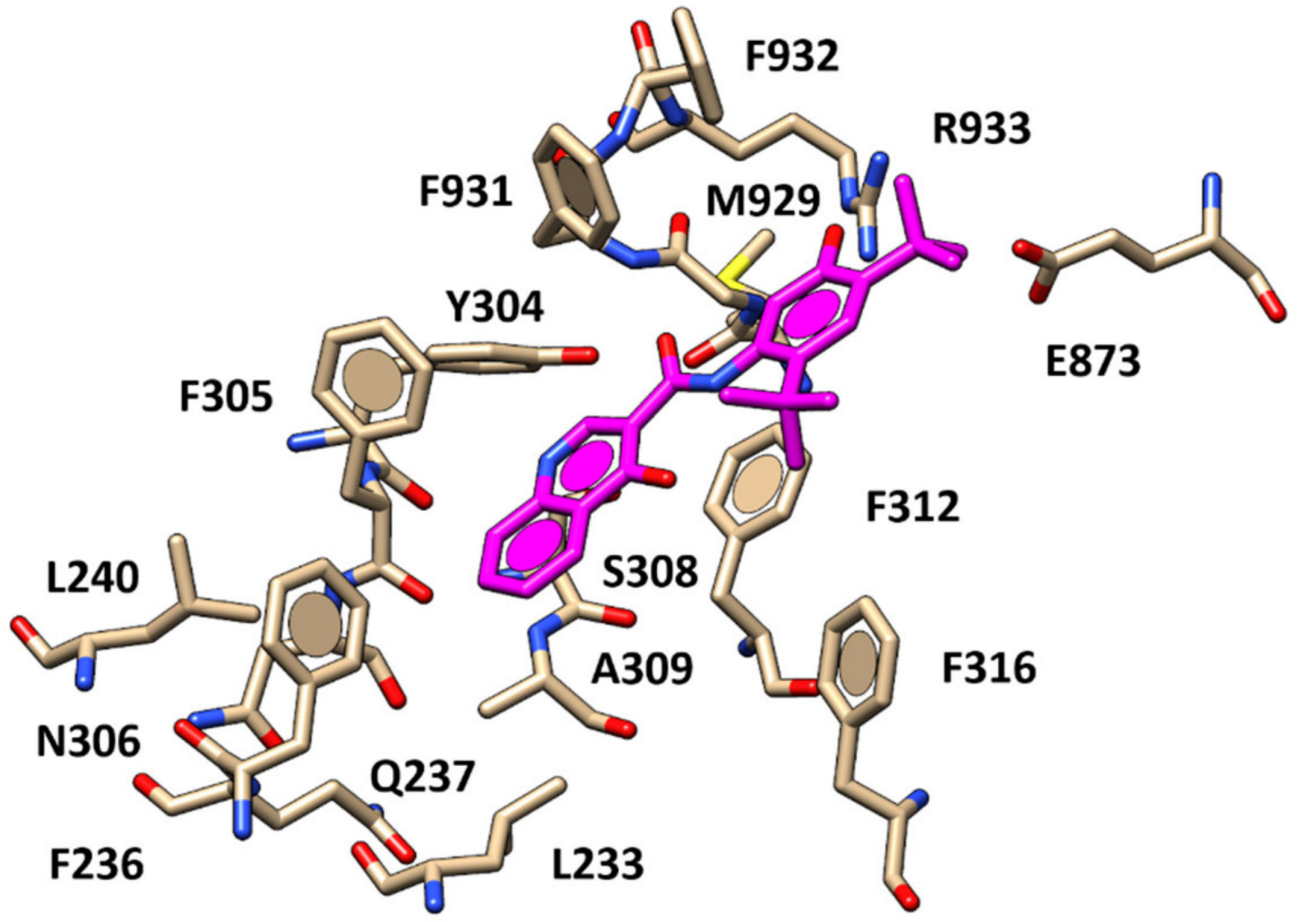
Refined docking positioning of VX-770 (*C* atom; magenta) within the human CFTR channel.

As shown in Figure 3, the quinolinone ring occupied a highly hydrophobic pocket delimited by F236, Y304, F305, S308, A309 and F312, detecting several Van der Waals and π–π stacking interactions. Moreover, the carboxamide moiety of the potentiator was H-bonded to the backbone of F931, while the hydroxyl group of the potentiator displayed one H-bond with R933. The *tert-*butyl substituents and the phenolic ring were projected towards F931 and F932, establishing hydrophobic contacts. 

The most relevant binding requirements identified within the collected potentiators’ database have been proposed based on the comparison of the obtained docking pose with the aforementioned VX-770 positioning. In this way, we investigated the most effective chemo-types able to mimic the bioactive conformation and contacts experienced by the reference potentiator VX-770 (pEC_50_ = 6.63 M). Details of the obtained scoring functions have been reported in Supplementary Tables S8–S13. A perspective of these values proved to be in good agreement with the experimental activity supporting the docking protocol at the two putative binding cavities.

The GLPG1837 analogues (**1**–**26**; pEC_50_ = 6.10–9.26), including thiophene-based compounds, represent the most active potentiators within the CFTR modulators series herein studied (Supplementary Table S1). Most of them shared the same docking positioning within the CFTR channel, featuring in any case key contacts with F931, as previously described for VX-770.

**Figure 4:**
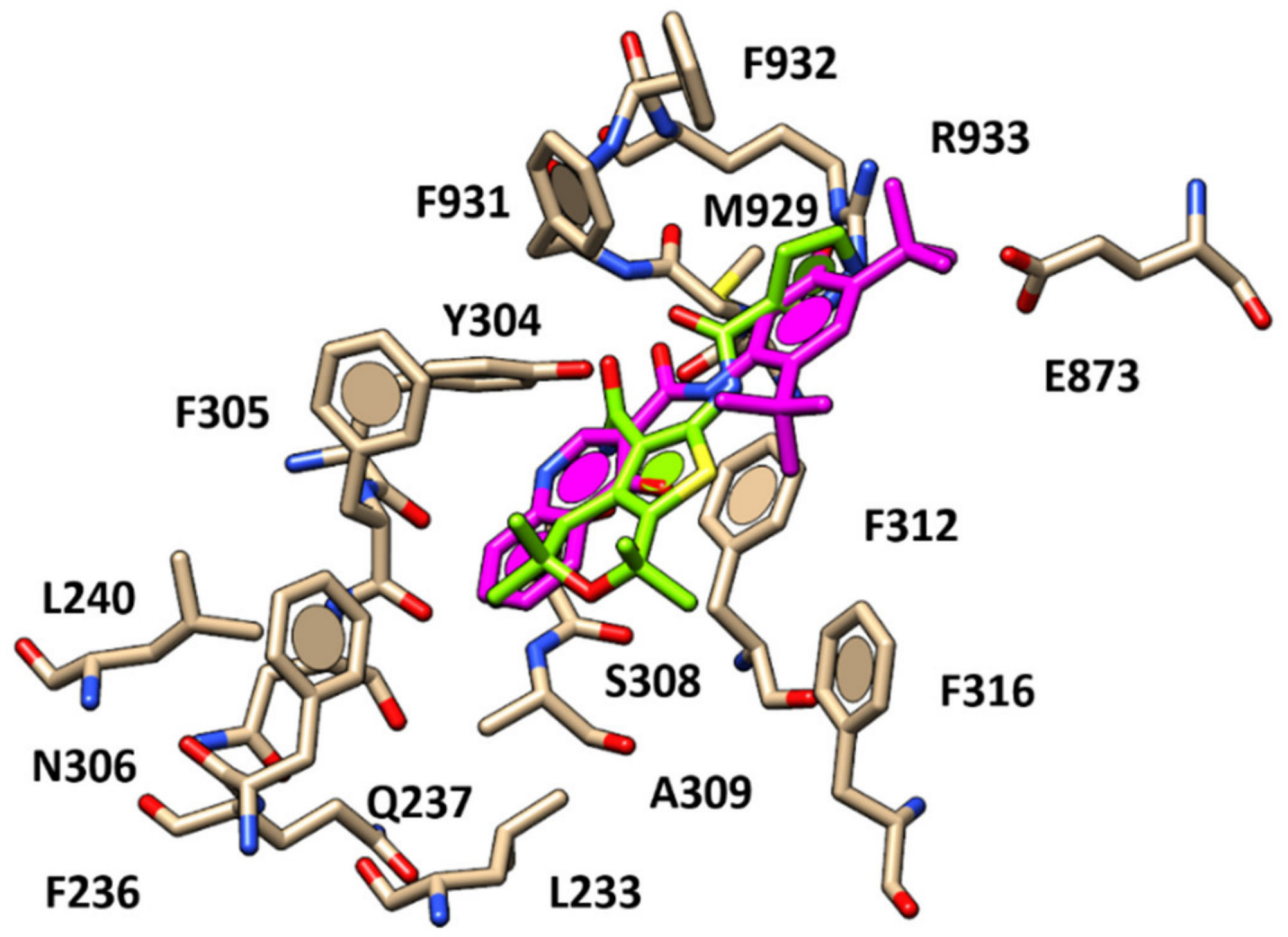
Docking positioning of compound **11** (*C* atom; green) within the X-ray crystallographic structure of the human CFTR in complex with VX-770 (*C* atom; magenta).

In general, compounds exhibiting the 5,5,7,7-tetramethyl-4*H*-thieno[2,3-c]pyran scaffold (**6**–**26**, pEC_50_ = 6.33–9.26) displayed higher potency than congeners bearing less hydrophobic bicyclic cores, such as **1**–**4** (pEC_50_ = 6.10–7.88). Among the 5-carboxamido pyrazole-containing analogues **10**–**12**, **22**–**26** (pEC_50_ = 6.86–8.69), compound **11** (pEC_50_ = 8.52) displayed two H-bonds between the two oxygen atoms of the carbonyl moieties and the F931 backbone as well as polar contacts with S308, and π–π stacking involving the same residue F931, F932 and the pyrazole substituent (Figure 4).

**Figure 5:**
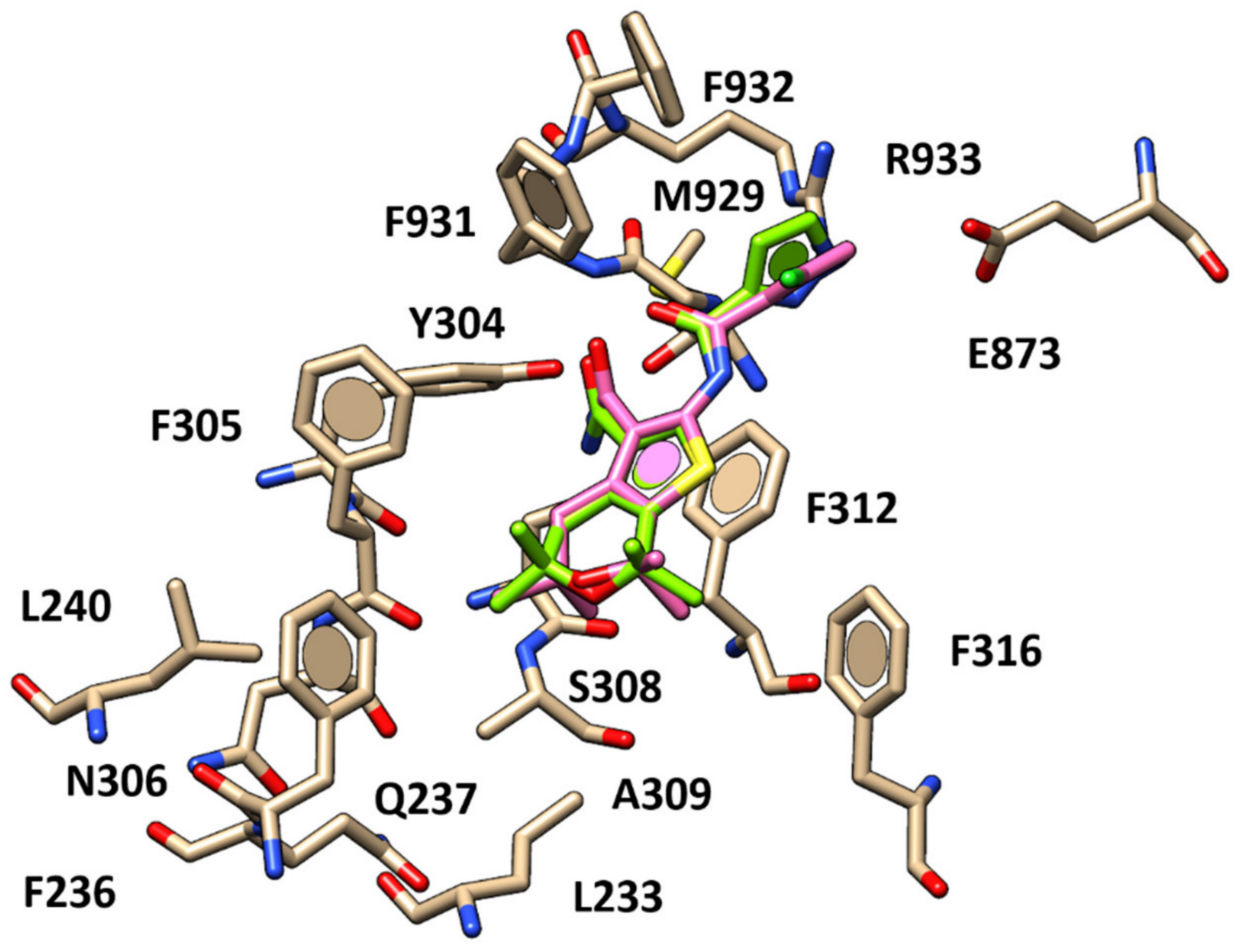
Docking positioning of compound **22** (*C* atom; pink) compared to that of compound **11** (*C* atom; green) within the human CFTR protein.

In addition, the pyrazole substituent and the potentiator primary amide group were H-bonded to the R933 and Y304 side-chains, while the thienopyran-based core was projected towards F236, F305 and F312, detecting π–π stacking and Van der Waals contacts, similarly to VX-770. As a consequence, the thienopyran ring of **11** was bioisostere of the quinoline ring of VX-770, while the presence of more H-bonding features such as two carboxamide moieties and the terminal pyrazole ring allowed **11** to be better stabilized at the biological target, interacting with R933 and Y304, making **11** (pEC_50_ = 8.52) more potent than VX-770 (pEC_50_ = 6.63). Interestingly, this kind of positioning was effectively maintained by most of the docking poses collected for those analogues featuring a CH3- or a Cl-substituent onto the position 4 of the pyrazole (**22**–**23**, pEC_50_ = 8.69) (Figure 5), then showing similar potency values to compound **11**. 

Conversely, the docking poses calculated for the 3-CH_3_ substituted pyrazole-containing (**25**–**26** pEC_50_ = 7.88–7.95) derivatives were lacking of any H-bond with R933, thus yielding lower pEC_50_ values than the previously cited 4-substituted analogues (**22**–**23**, pEC_50_ = 8.69) (Supplementary Figure S14).

The introduction of a 4-carboxamide- instead of a 5-carboxamide-pyrazole substituent linked to the main bicyclic core, such as **10** (pEC_50_ = 6.90) compared to **11** (pEC_50_ = 8.52), or the introduction of a methylene tethering the pyrazole core to the amide moiety of the potentiator (see **12** (pEC_50_ = 6.86)), impaired the potency profile of the derivatives. This was motivated by the lack of any H-bonds with R933 for all the related docking poses (see Supplementary Figure S15). 

Choosing a triazole moiety instead of the pyrazole one led to the compound **9** (pEC_50_ = 6.33), featuring two H-bonds via the carbonyl oxygen atoms and the F931 side-chain, while the primary amide group detected only one H-bond with Y304. Notably, the triazole ring proved to mimic the behavior of the R substituent exhibited by **10** and **12**, showing comparable potency levels (see Supplementary Figure S16).

**Figure 6:**
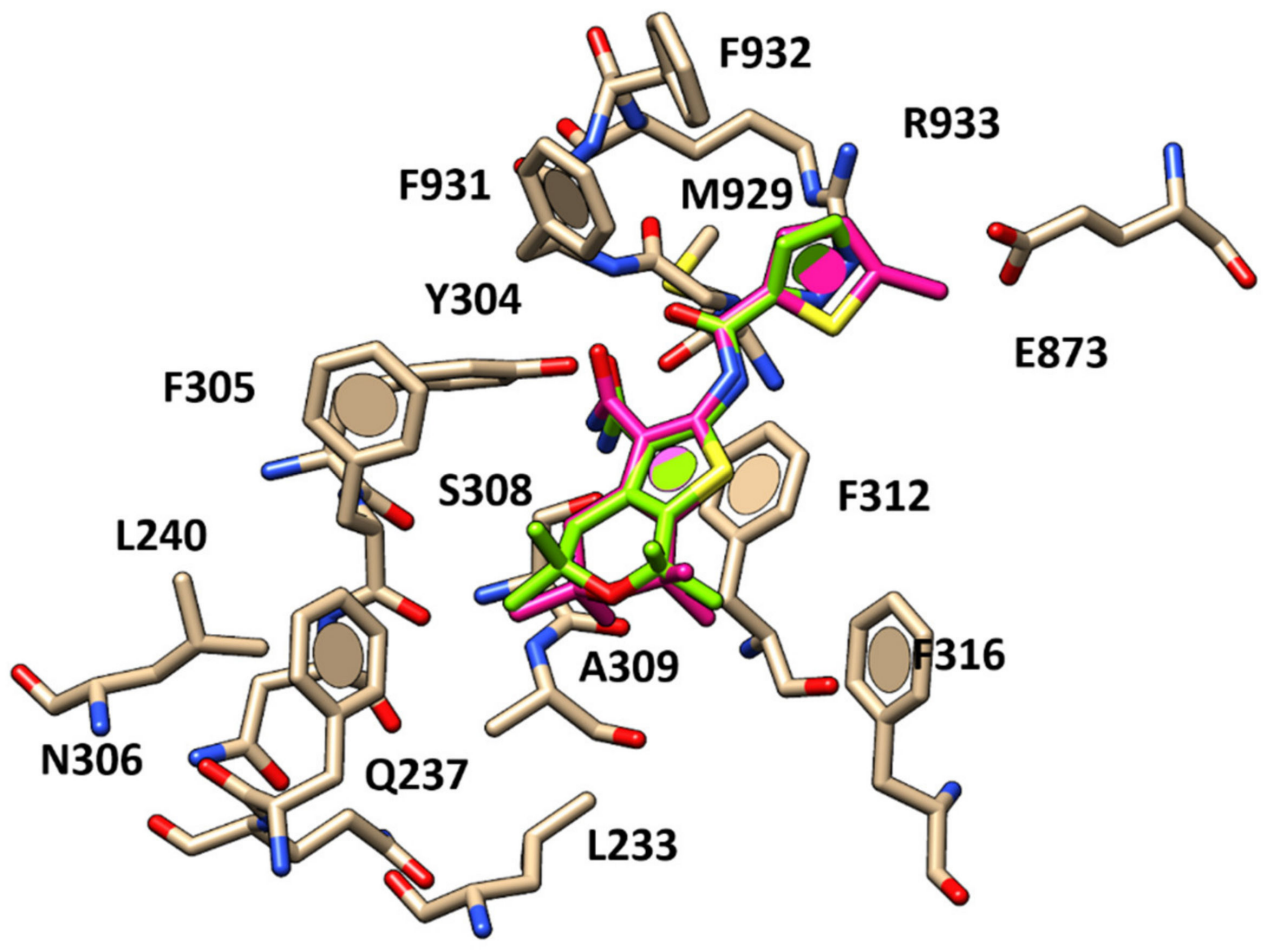
Docking positioning of compound **6** (*C* atom; deep pink) compared to that of compound **11** (*C* atom; green) within the human CFTR protein.

Moving from the five-membered ring derivatives (**9**–**14**; **22**–**26**; pEC_50_ = 6.33–8.69) to the six-membered ones (**4**–**8**; pEC_50_ = 6.72–8.39) allowed the preservation of the promising potency values as F508del-CFTR potentiators. In particular, **4**–**6** featured the two key contacts with F931 and the H-bond with Y304. Even if they were not able to interact with R933, these potentiators were efficiently stabilized within the biological target thanks to cation-π contacts with the same residue (Figure 6).

**Figure 7:**
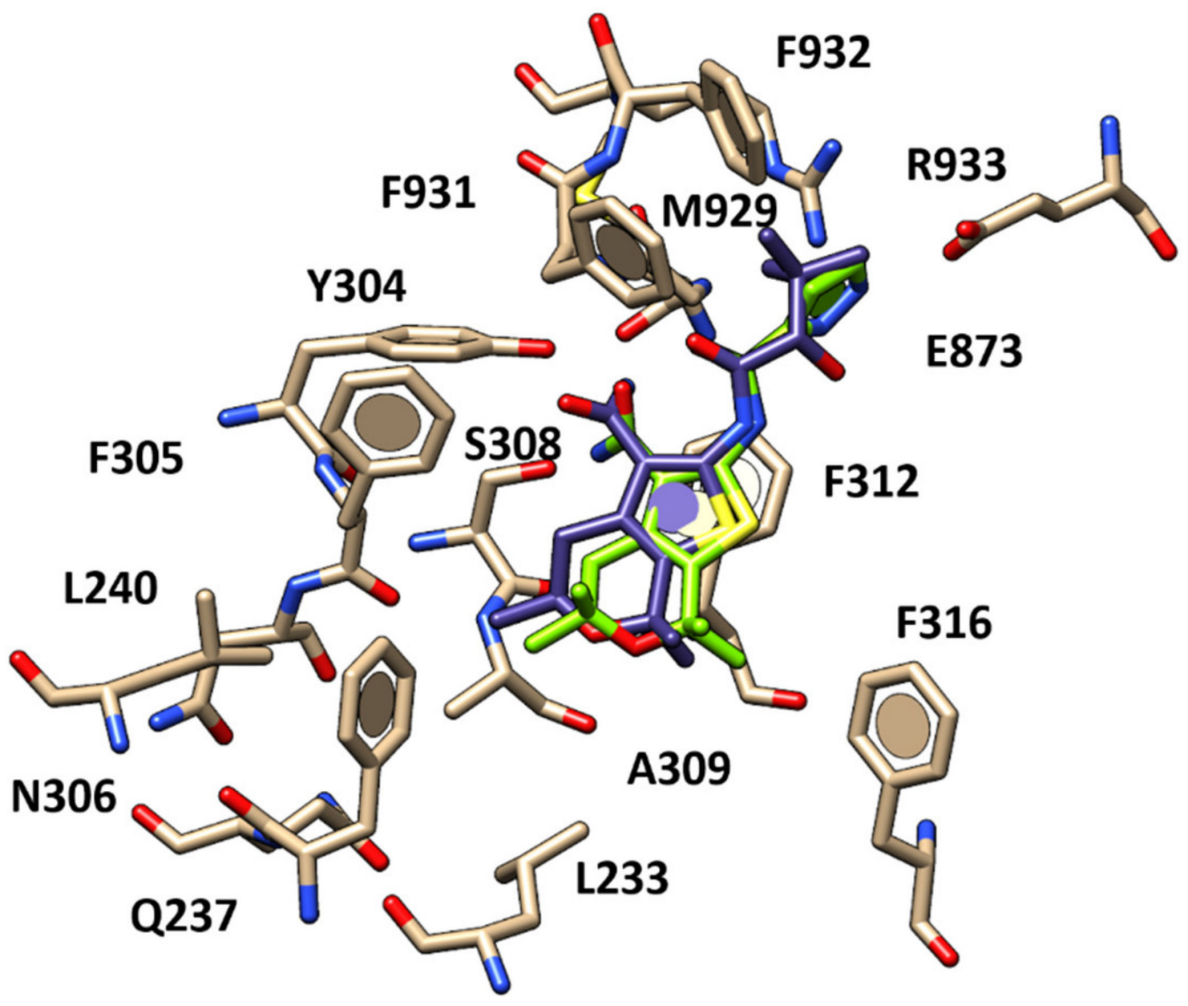
Docking positioning of compound **20** (*C* atom; deep purple) compared to that of compound **11** (*C* atom; green) within the human CFTR protein.

The relevance of H-bonding features decorating the benzoyl moiety connected to the carboxamide functions was confirmed by the adequate potentiator ability, as experienced by those derivatives bearing an alcohol group **15**–**21** (pEC_50_ = 6.89–9.26), sometimes displaying comparable interactions with those shown for VX-770 and for the effective analogue **11**. As shown in Figure 7, the compound **20** (*S* enantiomer) (pEC_50_ = 8.56) displayed one H-bond between the hydroxyl group and the R933 side-chain, mimicking the role played by the VX-770 hydroxyl or by the pyrazole group of **11**, while the **19** (*R* enantiomer) (pEC_50_ = 6.89) was unable to detect this contact (see Supplementary Figure S17).

As regards the compound **21** racemic mixture (pEC_50_ = 9.26), the *S* enantiomer was predicted to be more effective than the *R* enantiomer, giving H-bonds with R933 and with Y304, S308 and F932 thanks to the alkoxy group and the two carboxamide moieties (see Supplementary Figure S18).

This high number of interactions with the biological target guarantees a high level of potency, with **21** being more effective than **11**. 

The removal of any H-bonding moieties at the amide group tethered to the main bicyclic core when accompanied by less hydrophobic R substituents, such as compounds **1**–**3** (pEC_50_ = 6.10–6.18), led to impairment of potency as F508del CFTR potentiator. Accordingly, compound **3** only partially mimicked the positioning of VX-770, being characterized by a limited number of hydrophobic contacts and featuring H-bonds with F931 and Y304. 

The second class of potentiators evaluated herein includes cyanoquinoline-containing (CQ) derivatives. All the compounds show a moderate potency as F508del-CFTR potentiator in comparison with the first class (Supplementary Tables S2 and S3).

In particular, compounds **27**–**56** (pEC_50_ = 4.25–5.92) include CQ derivatives, featuring a modest potency as F508del-CFTR potentiators in comparison to the aforementioned series of thienopyran-based compounds (**1**–**26**; pEC_50_ = 6.10–9.26). According to our molecular docking calculations, this potency range may be explained by the CQ ability to interact only with F931 and sometimes with R933, lacking any contact with Y304 or S308. 

In particular, the most potent CQs (**29** and **33**–**56** pEC_50_ = 4.25–5.92) bore a 5,7-dimethylquinoline-3-carbonitrile main core, being more effective than the congeners characterized by a monomethyl-substituted quinoline ring, such as 8-methylquinoline-3-carbonitrile analogue **27** (pEC_50_ = 4.82) and the 6-methylquinoline-3-carbonitrile derivative **28** (pEC_50_ = 4.82).

**Figure 8:**
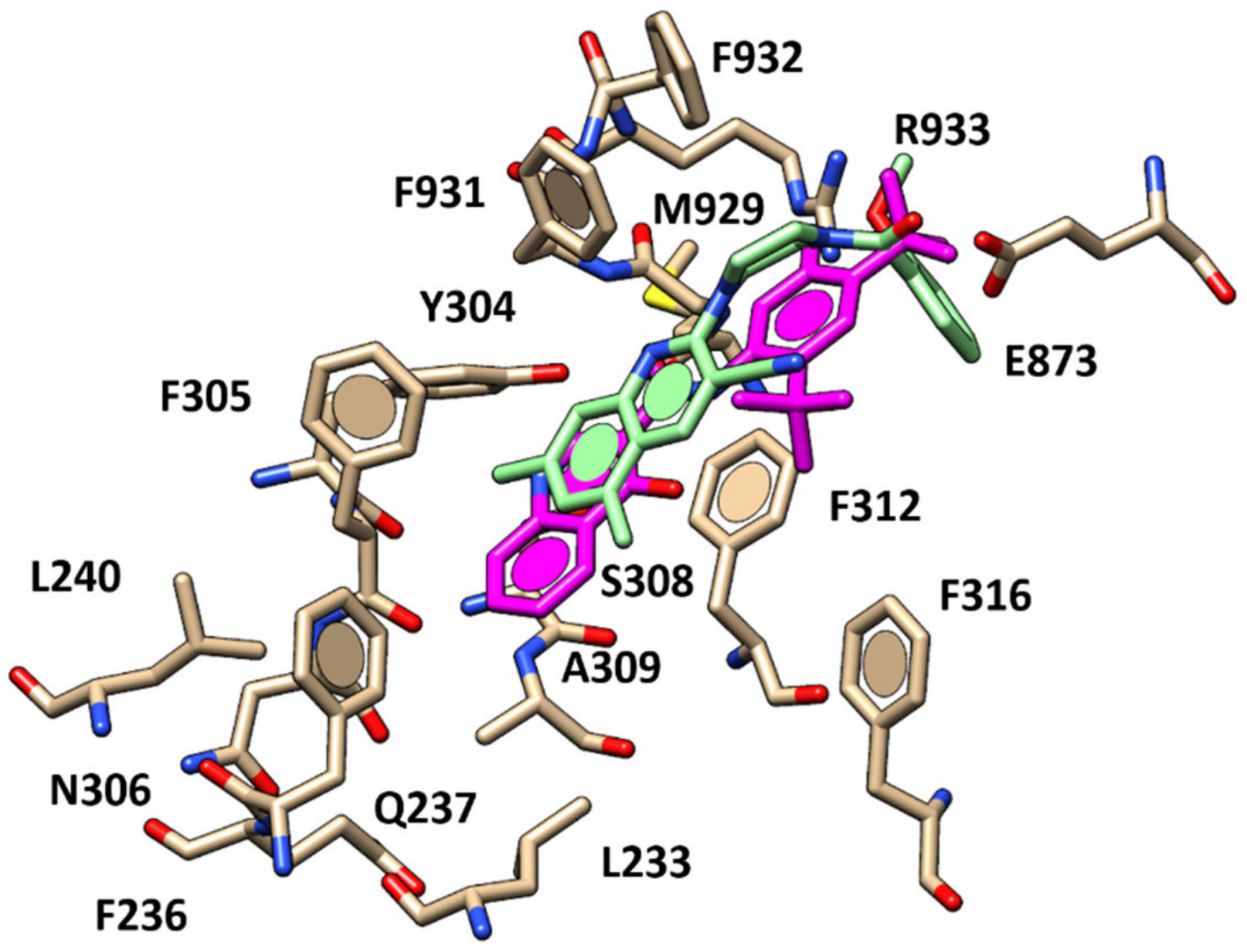
Docking positioning of compound **45** (*C* atom; light green) within the X-ray crystallographic structure of the human CFTR in the presence of the docked VX-770 (*C* atom; magenta).

Among them, the presence of a rigid spacer between the bicyclic core and the terminal aromatic ring allowed to derive effective 5,7-dimethylquinoline-3-carbonitrile-containing derivatives, as confirmed by the promising potency values of the methoxy-benzoyl substituted **45**–**47** (pEC_50_ = 5.88–6.00). As shown in Figure 8, the piperazine ring of the potentiator **45** moved the terminal benzoyl group in proximity of R933, detecting H-bonds and cation-π contacts. 

On the other hand, the 5,7-dimethylquinoline main core only partially mimicked the hydrophobic contacts and π–π stacking featured by the VX-770 quinoline scaffold, while the nitrogen atom of the bicyclic ring of **45** was properly H-bonded to the F931 backbone. Therefore, the potency profile shown for **45** (pEC_50_ = 6.00) was lower than that of VX-770 (pEC_50_ = 6.63).

Interestingly, the isosteric substitution on the benzoyl group with a pyridine one deeply impaired the potency trend within the piperazine-containing 5,7-dimethyl-3-carbonitrile CQs, lacking the previously cited contacts. This information is confirmed by the lower pEC_50_ of **48** (pEC_50_ = 5.13) and by the inactive analogs **49**–**51**. In particular, the picolinamide group of **48** was the only one engaged in key contacts with F931 (see Supplementary Figure S19).

The relevance of the methoxy benzoyl substituents (probably in terms of balanced hydrophobic-hydrophilic properties at the potentiator surface and of the related ligand positioning) is also confirmed within the CQs bearing the 1,2-ethylendiamine (**33**–**35**; pEC_50_ = 5.22–5.63), with **33**–**35** being more potent than the unsubstituted benzoyl congener **39** (pEC_50_ = 5.00) and the dimethoxy benzoyl substituted analogues **40**, **41**, **44** (pEC_50_ = 4.25–4.93). As shown in Supplementary Figure S20, the flexible spacer of **34** allowed the benzoyl group to detect one H-bond with R933, while the nitrogen atom of the quinoline moiety was H-bonded to F931. 

This kind of positioning guarantees the same contacts for **45** as well as for **34** at the CFTR protein, except for cation-π between the benzoyl group of **34** and R933, turning in lower potency of this potentiator. 

The elongation of the linker by means of introduction of the 1,3-propylendiamine chain allowed the yield of potentiators **51**–**53** (pEC_50_ = 4.55–5.33), decorated with a methoxy-benzoyl ring which featured an adequate F508del-CFTR potentiator potency, while the related pyridine analogues **54**–**56** (pEC_50_ = 4.79–5.21) were somehow endowed with lower pEC_50_ values. Among them, the docking mode observed for **53** (pEC_50_ = 5.30) was highly comparable with that previously described for **34** (pEC_50_ = 5.63), therefore exhibiting similar potency. Indeed, **53** was H-bonded to the R933 side chain, while the quinoline nitrogen atom to F931. In addition, several π–π stacking involving the Y304, F305 and F312 were detected by the aforementioned quinoline core.

Finally, the introduction of the 6-methoxyquinoline-3-carbonitrile ring, instead of the 5,7-dimethyl-3-carbonitrile one, led to moderately active potentiators, when accompanied by the piperazine spacer, as shown by **30**–**32** (pEC_50_ = 4.95–5.33). Indeed, based on our computational study, compounds **30**–**32** exhibited a comparable positioning, moving the benzoyl moiety towards R933 to display one H-bond with this key residue, while the bicyclic ring showed π–π stacking with Y304, F305 and F312 (Supplementary Figure S21).

A further series of F508del-CFTR potentiators explored herein includes the anellated tetrahydropyridoindole-based compounds (**57**–**69**; pEC_50_ = 4.52–5.69), that were endowed with lower pEC_50_ values than the GLPG analogues (**1**–**26**; pEC_50_ = 6.10–9.26) and with comparable potency to CQ derivatives (**33**–**56**; pEC_50_ = 4.25–5.92) (Supplementary Table S4). 

Among them, the benzyl-substituted derivatives **57**–**59** (pEC_50_ = 5.00–5.69) and **61**–**63** (pEC_50_ = 4.52–4.88) were more potent than **64**–**69** (pEC_50_ = 4.52), featuring benzoyl rings or other alkyl-based substituents. In particular, the most promising derivatives **57**–**59** were also decorated with a methoxy substituent onto the indole portion.

**Figure 9:**
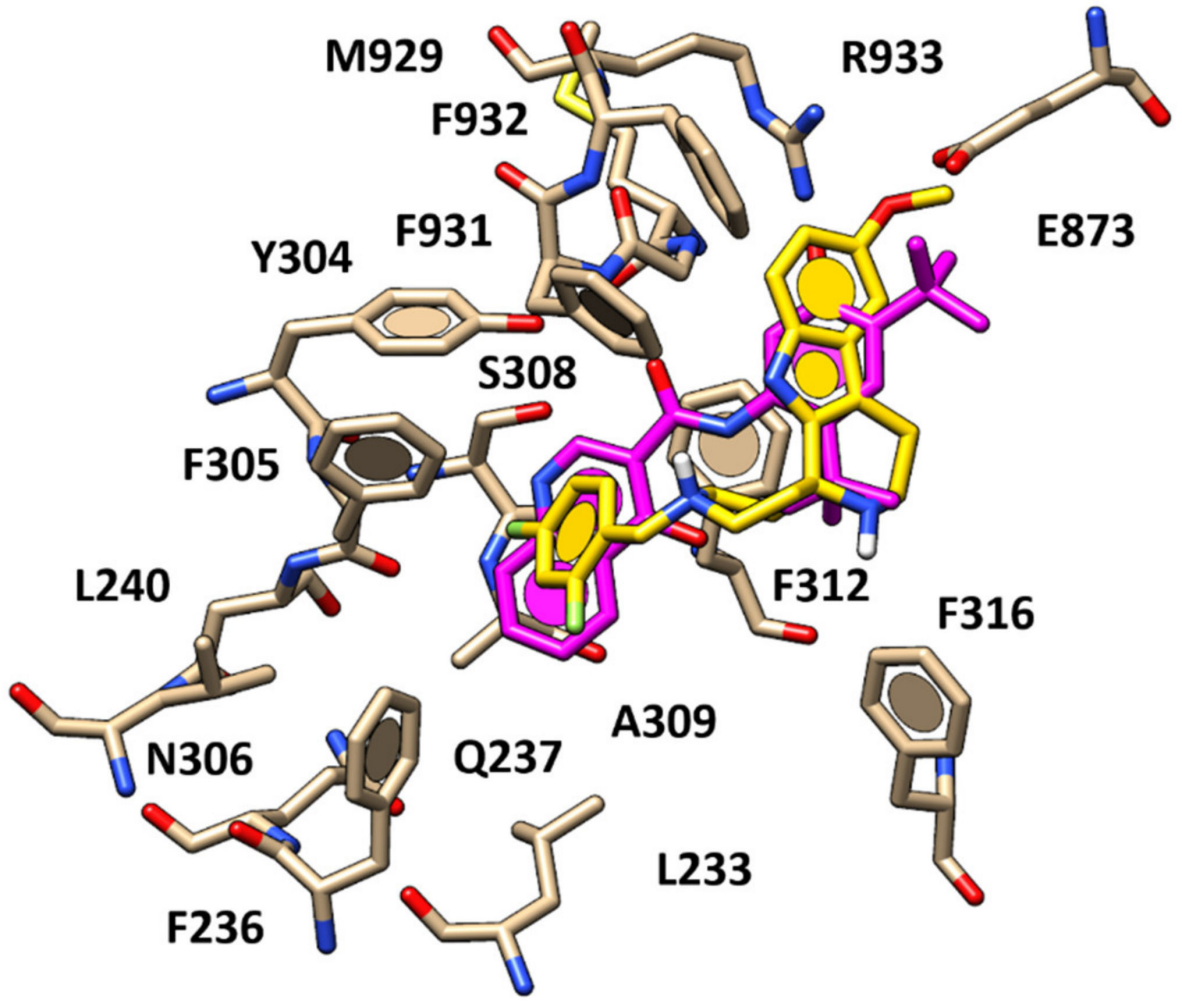
Docking positioning of compound **58** (*C* atom; yellow) within the X-ray crystallographic structure of the human CFTR in the presence of the docked VX-770 (*C* atom; magenta).

As a consequence, the related docking poses allowed the enlightenment of a predominant role played by a quite flexible aromatic group linked to a basic nitrogen atom as R1 substituent, which is the potentiator involved in hydrophobic contacts and π–π stacking with F236, Y304, F305 and A309. In addition, for the most promising compounds, such as **58**, the previously mentioned protonated nitrogen atom showed cation-π interactions with F931, playing a key role to stabilize the ligand at the CFTR channel, while the methoxy group in R3 was H-bonded to R933 (Figure 9).

In addition, the 2,4-di-F-benzyl moiety of **58** proved to highly mimic the quinoline ring of VX-770, while the tetrahydropyridoindole maintained a comparable positioning with respect to the *tert*-butyl-substituted phenyl ring of VX-770. Thus, compound **58** experienced Van der Waals contacts and π–π stacking with F312, F931 and F932. On the other hand, the absence of polar contacts with S308 and of H-bonds with F931 motived the lower potency of **58** and of this series of derivatives if compared to VX-770 ore GLPG1837 analogues. 

The 2,4-di-F derivatives **58** (pEC_50_ = 5.69) and **59** (pEC_50_ = 5.58) display comparable docking poses and higher potency values than the mono-substituted congeners **57** (pEC_50_ = 5.00) or the isostere **60** (pEC_50_ = 5.29), bearing a thiazole ring instead of the benzyl ring. 

Removing any halogen atom from the benzyl moiety and also the methoxy group on the indole core led to the modest potentiators **61**–**63** (pEC_50_ = 4.52–4.88), probably because of the limited number of hydrophobic contacts and polar interactions with the aforementioned key residues, especially lacking H-bonds with R933. Thus, while compound **62** (pEC_50_ = 4.88) arranged within the protein crevice by H-bonds to Y304 and S308 thanks to the protonated nitrogen atom of the tetrahydropyrido moiety, the introduction of the carbonyl moiety in place of the benzyl group shown by compounds **65**–**67** (pEC_50_ < 4.52) impaired the effectiveness of the potentiators, despite the methoxy group in R3 (see Supplementary Figure S22). 

While both **64** and **67** maintained one H-bond with R933 by means of the methoxy group, the R1 substituent was in any case unable to exhibit cation-π contacts with F931. In particular, **67** brings an amide nitrogen atom in place of an amine one. As regards compound **64**, the cyclohexyl group in R1 impaired π–π stacking with the surrounding aromatic residues.

**Figure 10:**
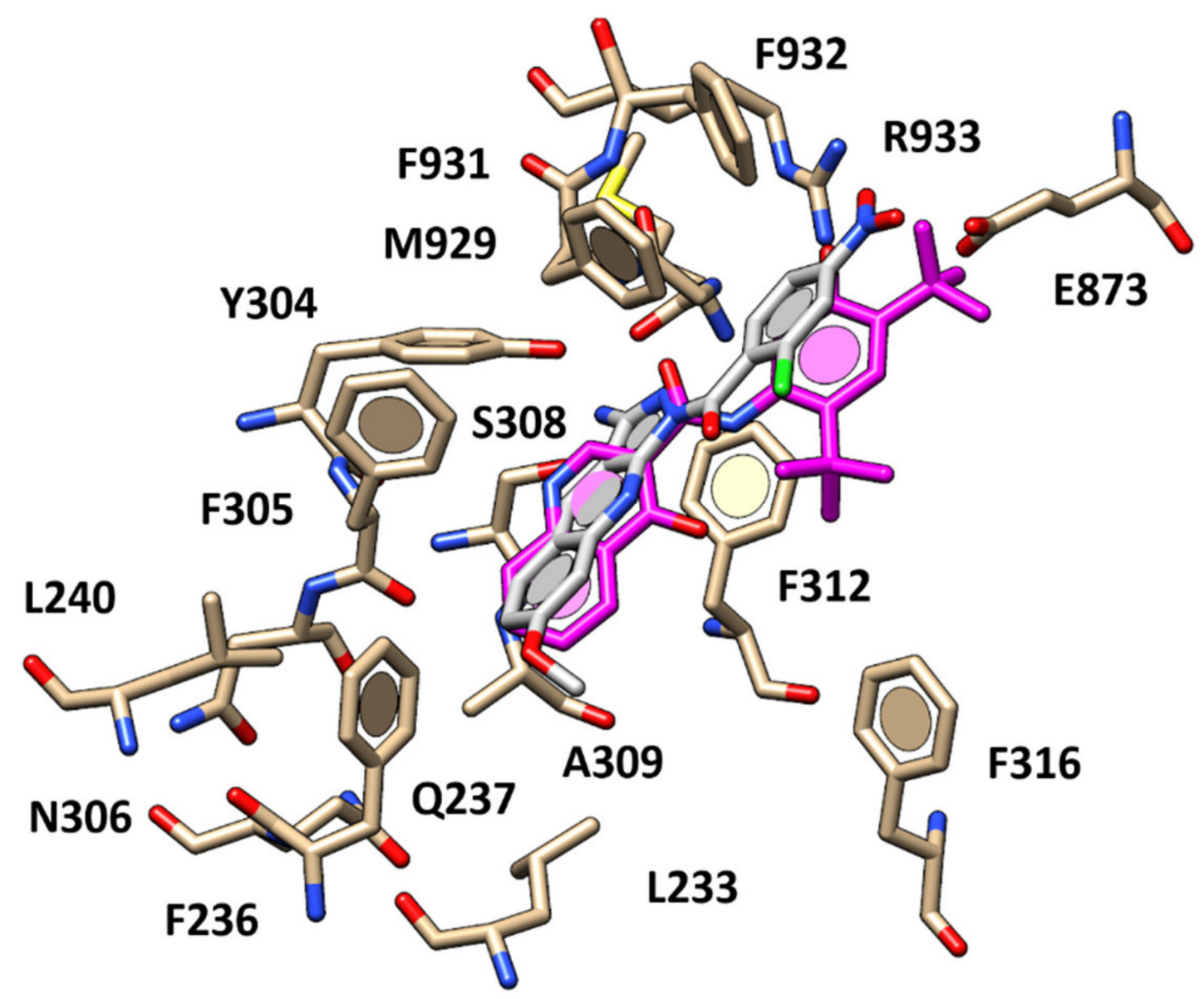
Docking positioning of compound **78** (*C* atom; light gray) within the X-ray crystallographic structure of the human CFTR in the presence of the docked VX-770 (*C* atom; magenta).

Concerning the pyrazolo-quinolines **70**–**80** (pEC_50_ = 4.52–6.52), the presence of lipophilic and electron-withdrawing groups at the positions 2 and 4 of the aromatic ring (R3 substituent) led to the promising derivatives **74** (pEC_50_ = 5.61) and **78** (pEC_50_ = 5.76), by properly positioning the potentiators within the CFTR cavity (Supplementary Table S5). Indeed, as shown in Figure 10, the compound **78** was able to mimic the carboxamide-substituted quinolone-based ring of VX-770, detecting H-bonds with S308 and M929 thanks to the primary amine group. 

In addition, the pyrazole portion of the tricyclic main core of **78** and the -NO2 group were H-bonded to F931 and R933 respectively, as the docking mode previously reported for VX-770. While the π–π stacking, such as with F236, Y304, F305, were maintained, other ones with F312 or cation-π contacts with R933 and Van der Waals interactions with A309 and F312 were weaker or lacking. These findings were in agreement with the quite lower potency experienced by **78** (pEC_50_ = 5.61) if compared to VX-770 (pEC_50_ = 6.63). Conversely, the higher potency value displayed by compound **80** (pEC_50_ = 6.52), bearing a bulky, aromatic and hydrophobic portion interacting with R933 through H-bonds as well as by cation-π contacts, was guaranteed thanks to a high number of interactions involving the R3 substituent and Y304, F305, A309, F312 and F316 (Supplementary Figure S23). 

In addition, the carbonyl group of **80** maintained the polar contacts with S308 and F931. 

Among the AATs featuring F508del-CFTR potentiator ability, we reported and discussed compounds **81**–**90** (pEC_50_ = 4.14–5.71) [22,23]. 

The introduction of a 4-substituted phenyl ring at the position 4 of the thiazole ring or of a bicyclic ring led to the most promising compounds (**82**, **89**–**90**; pEC_50_ = 4.56–5.71) with respect to 3-substituted derivative **83** (pEC_50_ = 4.27) (Supplementary Table S6). 

**Figure 11:**
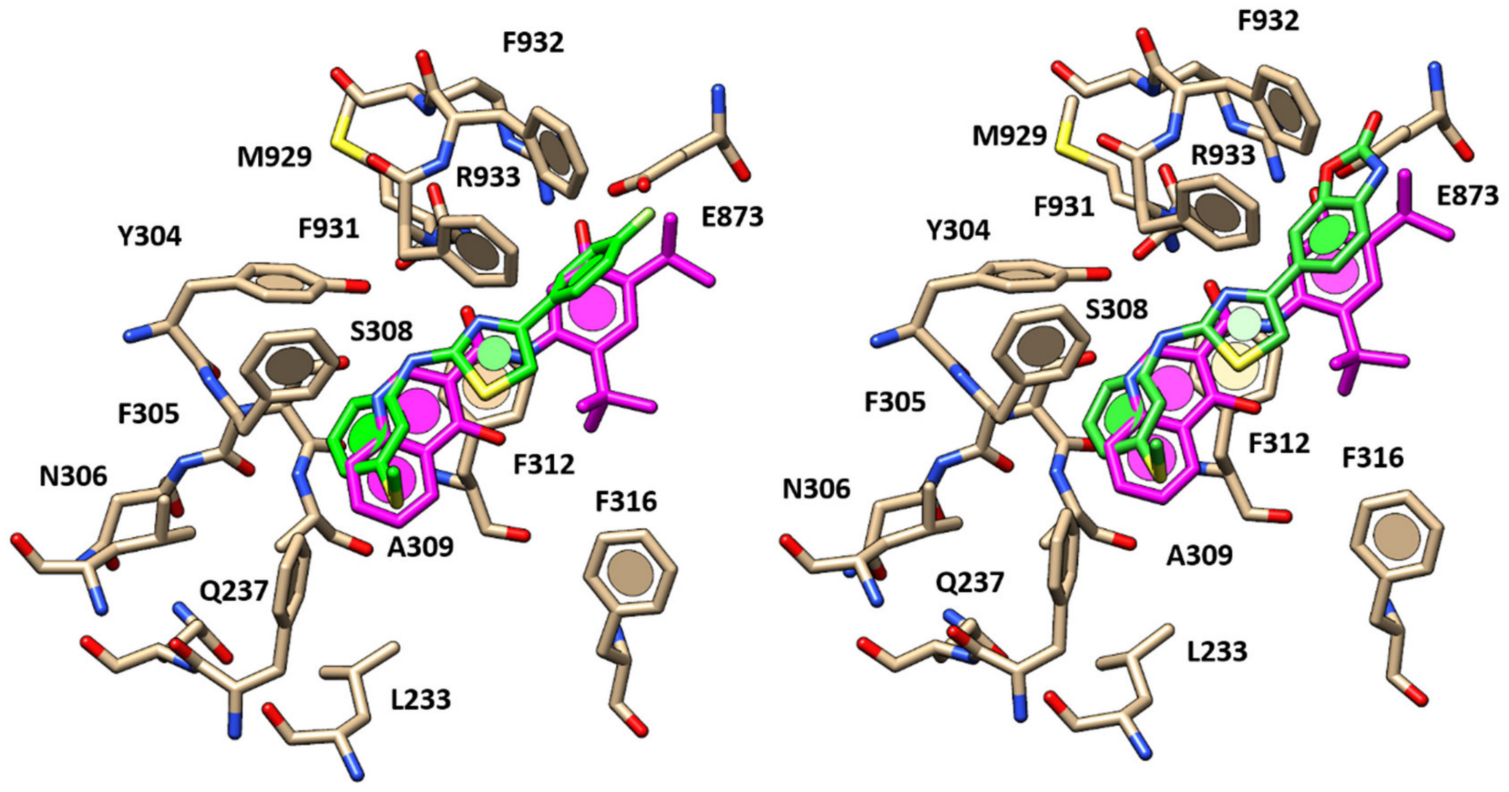
Docking positioning of compound **82** (**Left**) and **84** (**Right**) within the X-ray crystallographic structure of the human CFTR in the presence of the docked VX-770 (*C* atom; magenta). Both the two aminoarylthiazoles (AATs) re shown in stick, *C* atom in green.

As shown in Figure 11, **82** (pEC_50_ = 4.56) oriented the 4-F-phenyl group in proximity of the *tert*-butyl portion of VX-770, detecting the same hydrophobic contacts with F931 and F932. The main thiazole core was bioisostere of the reference compound carboxamide function, while the aminoaryl moiety of **82**, featuring the lipophilic thiomethyl substituent, was properly overlaid onto the quinolone group of VX-770. This allowed the compound to engage Van der Waals contacts and π–π stacking with F305 and F312. 

More interestingly, this kind of positioning guarantees H-bonds to the F931 backbone and to Y304 sidechain through the thiazole nitrogen atom and the amine group, respectively. The presence of a 4-CN group instead of the 4-F substituent onto the terminal phenyl ring switched the docking mode, maintaining π–π stacking with F931 and only one H-bond with S308 through the amine group. This finding was in agreement with the lower potency of **81** (pEC_50_ = 4.46) in comparison to **82**. 

**Figure 12:**
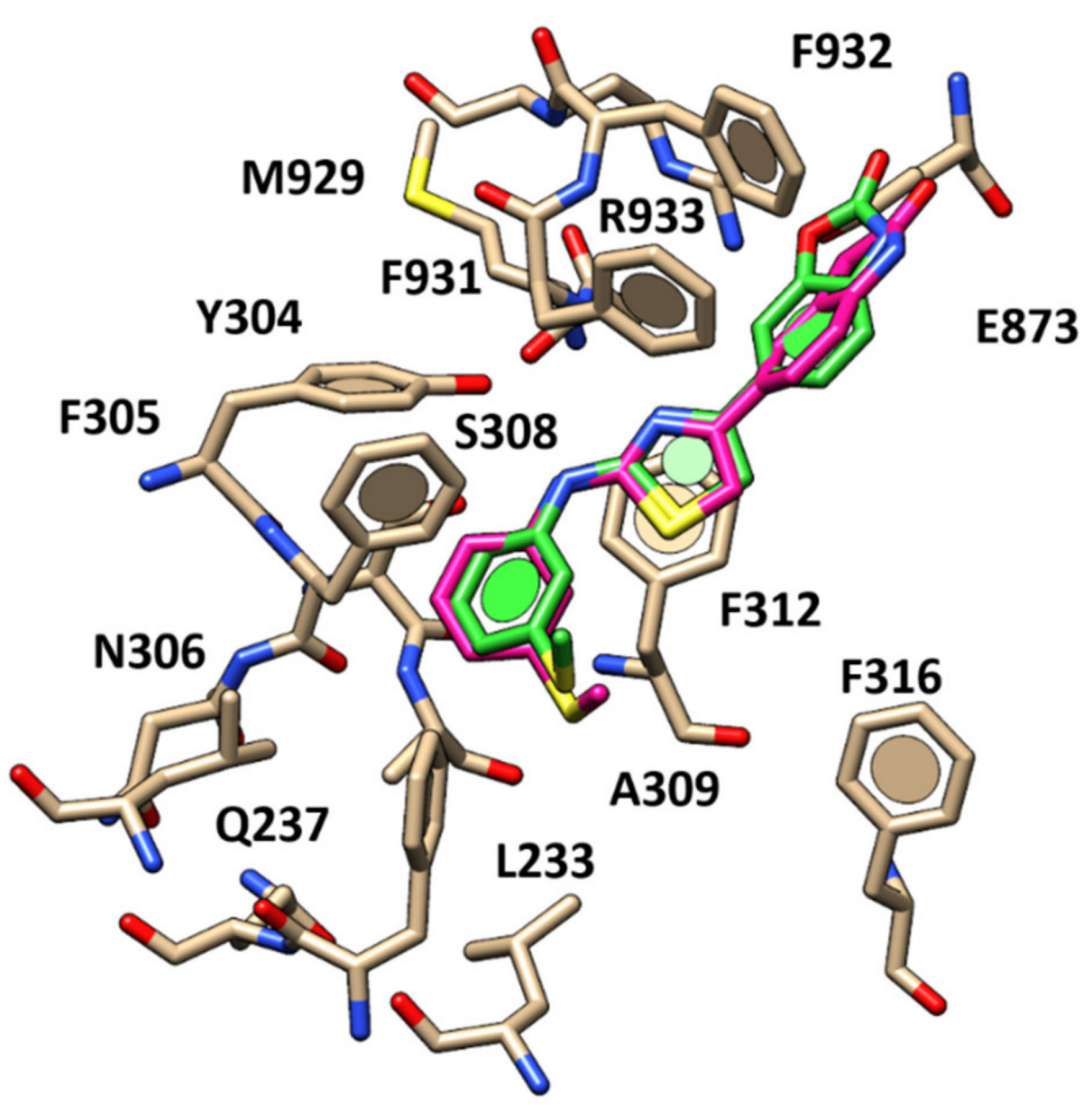
Docking positioning of compound **84** (*C* atom; green) and **89** (*C* atom; magenta) within the X-ray crystallographic structure of the human CFTR.

The introduction of the benzoxazolone ring at the position 4 of the thiazole ring led to one of the most potent AAT-based potentiator **84** (pEC_50_ = 5.19), characterized by the key H-bond involving the R933 side chain and the oxygen atom of the carbamate portion (Figure 12). 

This kind of effective positioning was accompanied by a higher number of hydrophobic contacts with CFTR, being also involved in more hydrophobic contacts with F931 and F932. 

Accordingly, the most promising derivative **89** (pEC_50_ = 5.71) experienced a comparable docking mode if compared to **84**, being endowed with higher hydrophobic contacts played by the indolinone moiety with respect to the benzoxazolone one of **84** (Figure 12). Conceivably, this allowed to better mimic the *tert*-butyl portion of VX-770.

The bioisostere replacement of the phenyl ring at the position 4 of the thiazole of **81**–**83** (pEC_50_ = 4.27–4.56) with a five-membered ring, such as **86**–**88** (pEC_50_ = 4.23–5.00), left the potentiator binding ability unvaried, which showed comparable docking mode and potency trend. Among them, compound **86** (pEC_50_ = 5.00) experienced two H-bonds with F931 and Y304 thanks to the thiazole nitrogen atom and the amine group. 

### 2.2. CFTR Potentiator Pharmacophore Model

Molecular docking studies have been accompanied by pharmacophore analysis focusing on the most effective thienopyrane-based derivatives **1**–**26**, exhibiting the higher potency values as potentiators (Supplementary Table S1). This study allowed us to gain new insights to ameliorate the different chemo-types investigated herein and also to elucidate useful guidelines for the rational design of more promising derivatives featuring the mandatory structural requirements, responsible for the F508del CFTR modulator ability. This was performed by considering the bioactive conformations of the compounds, based on the docking poses previously discussed. The alignment obtained for **1**–**26** is represented in Supplementary Figure S24, taking the potent thienopyrane **11** (pEC_50_ = 8.52) as a reference compound.

The derived pharmacophore model was generated using the pharmacophore consensus module integrated into the MOE software. The program is based on the identification and classification of the most shared recurrent pharmacophore features within the proposed set of molecules. Any pharmacophore group is classified by an identification code associated with the program (ID), the percentage by which this feature appears among the molecules taken into account (SCORE), by a radius that exemplifies the maximum space within which this functional group, can be placed within the ligand (RADIUS) and by a symbol that represents its role in terms of interaction with the receptor (EXPRESSION). 

**Scheme 1:**
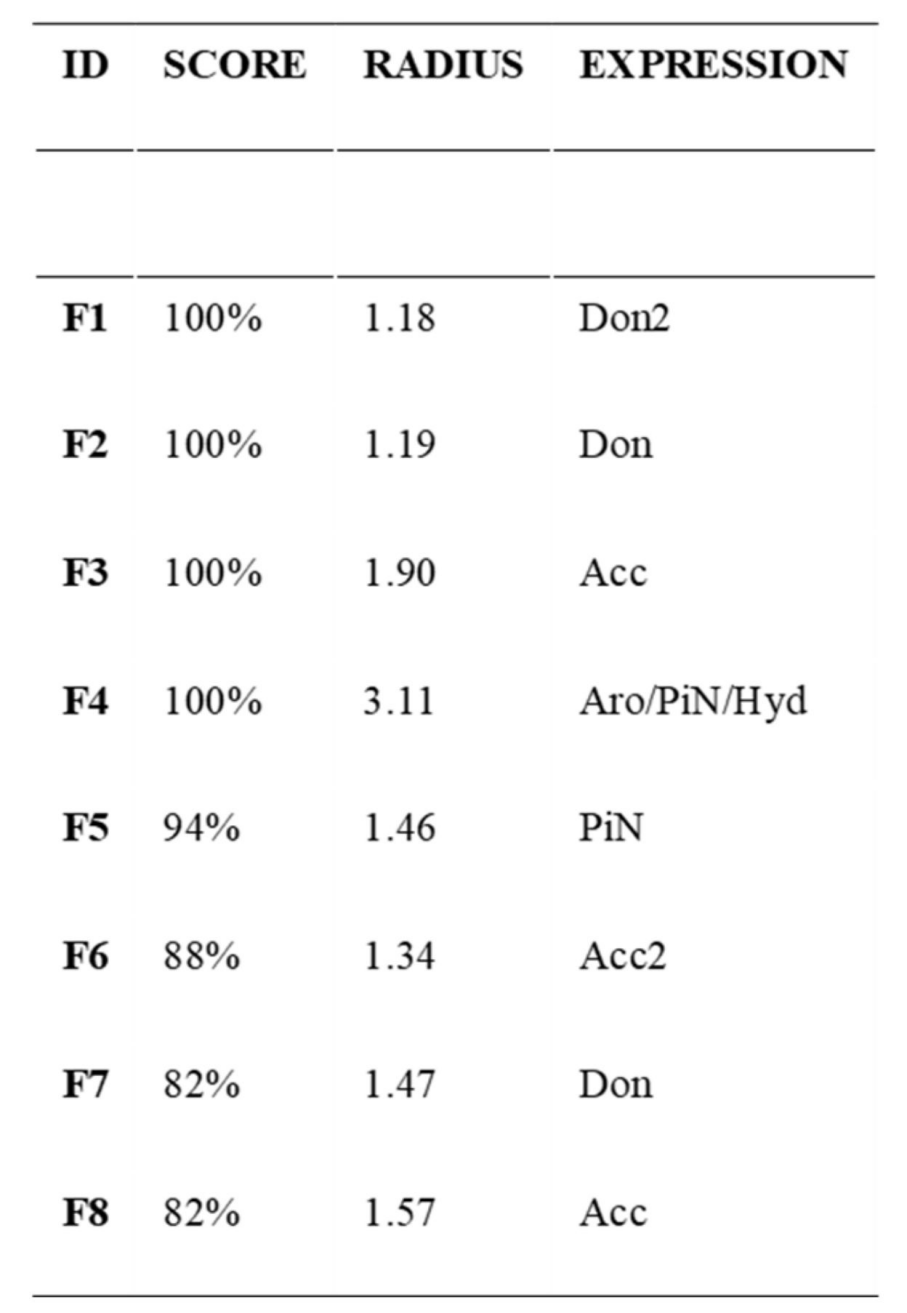
Detected pharmacophore features and the related ID, score, radius and expression.

As shown in Scheme 1, based on the data obtained, the most important pharmacophore requirements (represented by at least 80% of the molecules under examination) to draw a F508del CFTR potentiator include eight characteristic groups, especially H-bonding features properly tethered to (hetero)aromatic or hydrophobic groups.

**Figure 13:**
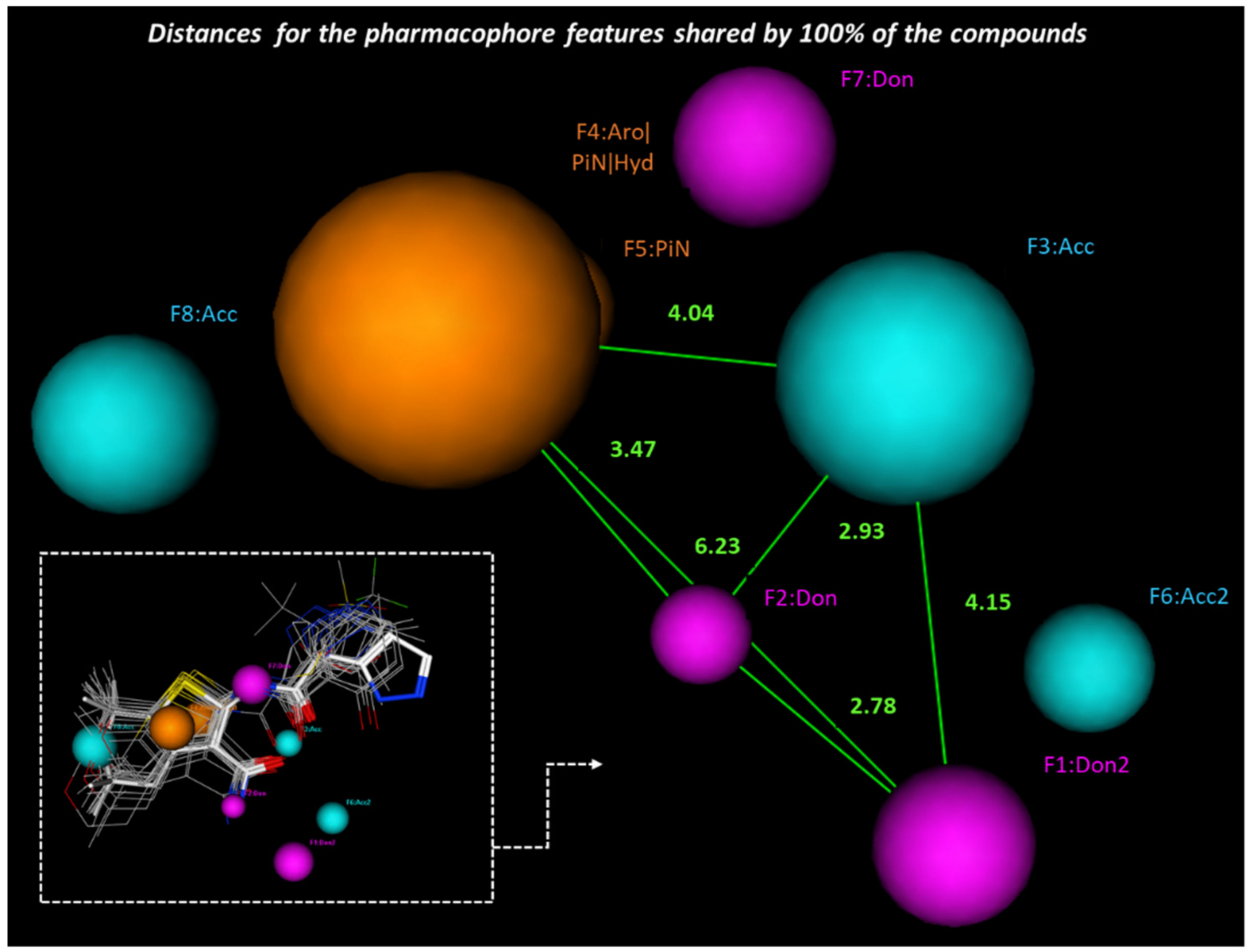
Most relevant pharmacophore features experienced and related distance by the thienoderivatives **1**–**26**, acting as CFTR potentiators.

In particular, Figure 13 shows a bulky (hetero)aromatic ring including H-bonding function (namely F4 Aro/PiN/Hyd) properly connected to H-bond donor (F2 Don and F1Don2) and acceptor features (F3 Acc), as exhibited by all the thienopyranes (see Scheme 1, score = 100%). Additional H-bonding features could decorate the main scaffold of the potentiator, as suggested by at least 80% of the derivatives, through F6Acc2, F7Don and F8Acc.

The expected reciprocal distances between F1 and F4 shared by all the derivatives included in the pharmacophore calculation reveal useful information for the further development of novel ligands (see Figure 13). Indeed, the main heterocyclic core exemplified by F4 should be at 6.23 Ǻ from the F1Don2 H-bonding donor projection, with respect to the F2Don placed at 3.47 Ǻ from the hydrophobic F4 ring, and at 4.04 Ǻ from the F3Acc.

On the other hand, in at least 82% of the congeners, the key F4 Aro feature has to be tethered to additional H-bonding groups, being placed between the acceptor and donor moieties, namely F8 (distance = 3.05 angstrom) and F7 (distance = 3.14 angstrom) (see Supplementary Figure S22). The key F1don2, F2don and F3acc polar requirements could be enriched by a further H-bond acceptor feature F6 placed at 2.21, 3.50 and 3.04 Ǻ, respectively (Supplementary Figure S25).

### 2.3. QSAR Analysis

In order to better explore the main features affecting the different potentiator ability displayed within the chemo-types **1**–**90**, we deemed it interesting to proceed with the development of a QSAR analysis. The derived QSAR model is expected to deepen our knowledge on the SAR of this potent series of CFTR modulators. Initially, this study was managed based on different descriptors referring to two-dimensional (2D) and three-dimensional (3D) parameters (three hundred molecular descriptors), which were estimated thanks to the MOE software [30]. 2D molecular descriptors can be divided in seven sub-series, related to physical properties (2D-I), subdivided surface areas (2D-II), atom and bond counts (2D-III), connectivity-based descriptors (2D-IV), partial charges descriptors (2D-V), pharmacophore features descriptors (2D-VI) and the so-called Adjacency and Distance Matrix Descriptors (2D-VII). 3D descriptors are classified in five different sub-groups, including potential energy descriptors (3D-I), Molecular Orbital PACkage (MOPAC) descriptors (3D-II), Surface Area (3D-III), Volume and Shape Descriptors (3D-IV) and Conformation-Dependent Charge Descriptors (3D-V).

The employed dataset included seventy-five compounds (**1**, **3**, **4**, **6**–**12**, **22**–**41**, **43**–**48**, **51**–**69**, **71**–**90**) chosen from the whole dataset (Supplementary Tables S1–S6), removing the totally inactive compounds and those bringing a chiral carbon atom. 

Thus, a final data matrix of 75 objects (compounds/molecules) and 306 rows (molecular descriptors) was obtained.

The chemometric package PARVUS [31] was applied to handle such information, in particular for checking the constant predictors, splitting the data into training and test sets, discarding the non-informative descriptors and extracting meaningful QSAR models able to identify the most important predictors accounting for the potentiator ability of the compounds in terms of pEC_50_ values (see Experimental Section). 

Following this approach, we identified 20 relevant descriptors, five of them belong to the 2D subdivided surface area (Table 1; SlogP_VSA4, SlogP_VSA5, SlogP_VSA9, SMR_VSA2, SMR_VSA4), four to the 2D Adjacency and Distance Matrix Descriptors (Table 2; BCUT_SLOGP_1, BCUT_SMR_1, BCUT_SMR_2, BCUT_SMR_3), two to the 2D Partial Charge Descriptors (Table 1; PEOE_VSA+5, PEOE_VSA-6), one to the 2D Pharmacophore Feature Descriptors (Table 2; a_hyd), one to the Physical Properties (Table 2; logS) and one to the 2D Atom Counts and Bond Counts (Table 2; b_1rotR). As regards the selected 3D descriptors, four of them fall in the Surface Area, Volume and Shape Descriptors (Table 3; vsurf_ID1, vsurf_ID7, vsurf_Wp2, vsurf_Wp3), while the other two were Conformation-Dependent Charge Descriptors (Table 3; dipoleY and ASA^+^).

All the selected 3D descriptors are related to the polarity profile of the modulators, extended along the molecular surface area, considering the conformational disposition of the ligands. On the other hand, all the 2D ones suggest balanced hydrophilic/hydrophobic properties onto the overall surface extent. 

The final QSAR model was developed splitting the compounds into a training and a test set using the space-filling Kennard-Stone duplex design [35], probably the most popular algorithm for the selection of a subset of samples with a distribution as close as possible to the uniform distribution. As a result, 60 objects were selected for the training set and 15 molecules were assigned to the test set (20% of the total molecules). 

In detail, the predictive model was calculated by including in the training set: **1**, **4**, **7**–**10**, **12**, **22**, **23**, **25**–**30**, **31**, **33**, **34**–**37**, **39**, **41**, **43**–**48**, **51**–**54**, **56**, **58**, **60**–**65**, **67**–**69**, **71**, **73**–**76**, **78**–**83**, **85**–**87**, **89**, **90**, which have been used to generate the QSAR model, and in the test set: **3**, **6**, **11**, **24**, **32**, **38**, **40**, **55**, **57**, **59**, **66**, **72**, **77**, **84**, **88**, in order to evaluate the reliability of the mathematical relationship. In particular, the final model was generated by employing non-cross-validated partial least square (PLS) analysis to give a cross-validated r^2^ (r^2^_cv_) = 0.74, a non-cross-validated r^2^ (r^2^_ncv_) = 0.90, root mean square error (RMSE) = 0.347 and a test set r^2^ (r^2^_pred_) = 0.86. The predicted and experimental rescue ability for all the derivatives are listed as tables together with the collected descriptors, as shown in Supplementary Data (see Supplementary Tables S26–S29).

Quantitatively, the potentiator ability of the compounds studied here is explained by the following equation (Equation (1)):

pEC_50_ = Potency = −2.51363 + 1.87312 × BCUT_SLOGP_1 + 1.58432 × BCUT_SMR_1 + 0.09344 × BCUT_SMR_2 + 1.48927 × BCUT_SMR_3 − 1.39449 × b_1rotR − 0.27817 × logS + 0.25870 × dipoleY + 0.01241 × a_hyd + 0.00253 × ASA^+^ + 0.00205 × PEOE_VSA+5 -0.02622 × PEOE_VSA-6 + 0.00522 × SlogP_VSA4 − 0.00628 × SlogP_VSA5 + 0.00393 × SlogP_VSA9 − 0.00921 × SMR_VSA2 − 0.00440 × SMR_VSA4 + 0.13782 × vsurf_ID1 + 0.41705 × vsurf_ID7 + 0.00211 × vsurf_Wp2 + 0.00957 × vsurf_Wp3 (1)

**Figure 14:**
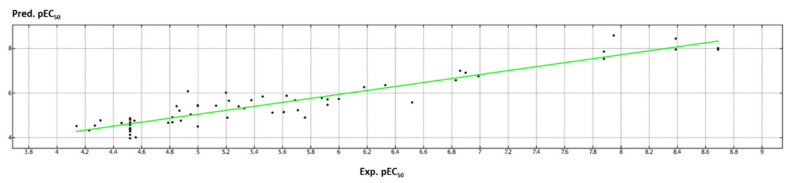
Distribution of the predicted (Pred.pEC_50_) and experimental (Exp.pEC_50_) potency values of the dataset compounds (shown as black dots).

A schematic representation of the high performance of the model is shown in Figure 14, giving a perspective of the high performance of the developed model when predicting the potentiator ability of the whole dataset (4.25 < pEC_50_ < 9.26).

Based on these results, the presence of quite flat groups decorated with polar substituents and endowed with a limited flexibility should be preferred to much more linear and flexible groups. This kind of information is in harmony with the need for a limited number of rotatable bonds and of hydrophobic atoms, as suggested by the B_1rotR and a_hyd descriptors in Supplementary Figure S30. 

Accordingly, the calculated mean values of the B_1rotR descriptor within the most potent thienopyrans (**1**–**26**; B_1rotR_mean_ = 0.119343), and of the flat pyrazoloquinolines (**70**–**80**; B_1rotR_mean_ = 0.131342), were lower than those of CQs (B_1rotR_mean_ = 0.182995), often exhibiting extended flexible chains (Table 4).

Indeed, the promising thienopyrans, **19**, **22**–**24** (pEC_50_ = 8.52–8.69), featured branched moieties or small aromatic rings as R substituent instead of linear aliphatic chain, while the most potent pyrazoloquinoline, **80** (pEC_50_ = 6.52), brings a thiophene-substituted quinoline as planar group in R3. As regards the less potent CQs, the choice of a piperazine spacer instead of diamino alkyl chain proved to be beneficial, as shown by **45**–**47** (pEC_50_ = 5.88–6.00) if compared to the diamino ethyl-based **33**–**35** (pEC_50_ = 5.22–5.63) and to the diamino propyl-containing **51**–**53** (pEC_50_ = 4.55–5.33). Along with this information, the optimal number of hydrophobic atoms should fall in a range spanning from 15 to 20 atoms (see Supplementary Figure S30). Higher values of this parameter could impair the potency profile of the compounds, as reported within the pyrazoloquinoline series exhibiting an a_hyd_mean_ value > 21.

Increasing values of the 3D descriptors Vsurf_ID7 and Vsurf_Wp3 turn in enhanced potency of the potentiators, supporting the key role played by balanced polar and hydrophobic features at the molecular surface. Conceivably, it is accompanied by differently substituted aromatic rings included in the main structure of the potentiator, as the (at least) three quite co-planar aromatic rings of thienopyrans **1**–**26**, pyrazoloquinolines **70**–**80** and AATs **81**–**90**. 

**Figure 15:**
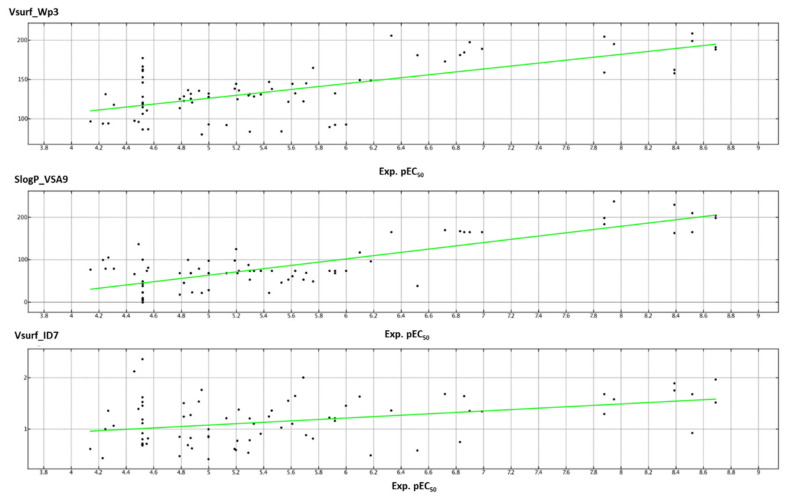
Distribution of the Vsurf_Wp3 (**top**), SlogP_VSA9 (**middle**) and Vsurf_ID7 (**bottom**) descriptor values with respect to the experimental (Exp.pEC_50_) potency data of the dataset compounds (shown as black dots).

A perspective of the distribution obtained for these descriptors points out optimal numbers of Vsurf_WP3 and Vsurf_ID7 as higher than 150 and 1.4, respectively (Figure 15).

These findings were supported by the related mean values (see Table 4) and are in agreement with the higher potency profile experienced by thienopyrans when compared to all the other classes of potentiators. On the other hand, the ideal value for the SlogP_VSA9 of at least 150 is predicted to be accompanied by pEC_50_ values > 7.00 M. 

Beyond these five descriptors, ASA+, logS and BCUT_SMR3 summarized the overall role played by balanced hydrophobic and polar properties (positively charged features) displayed by all the potentiators investigated herein. Interestingly, as shown in Supplementary Figure S31, the distribution of these calculated descriptor values with respect to the experimental potency of the compounds highlights a specific range of ideal well-tolerated values, rather than a limited threshold, to achieve the proper electrostatic and steric requirements.

Thus, effective potentiators should be characterized by ASA^+^, LogS and BCUT_SMR3 values spanning from 350 to 410 Å^2^, −5 to −4, and 2.9 to 3.0, respectively.

### 2.4. Prediction of ADMET Properties

Up to now, exploiting computational methods for the in silico prediction of descriptors related to the pharmacokinetic (PK) and toxicity profile of small molecules has offered a useful tool to identify drug-like compounds [36], as we previously performed for other case studies [37,38,39].

Thus, herein, we explored the putative absorption, distribution, metabolism, excretion and toxicity (ADMET) profile of the most promising GLPG1837 analogues, **1**, **3**, **4**, **6**–**12**, **22**–**26**, as well as of the VX-770, taken as a drug reference. 

In this work, for the aforementioned thienopyranes, the logarithmic ratio of the octanol-water partitioning coefficient (cLogP), solubility in water (mg/mL), the ability to reach and pass the blood–brain barrier (BBB) in terms of BBB permeation (LogBB) and rate of passive diffusion-permeability (LogPS), absorption at human intestinal level (HIA), volume of distribution (Vd), the role played by plasmatic protein binding (%PPB) and the ligand affinity predicted toward the human serum albumin (LogKa ^HSA^) were calculated, in order to assess a putative value of the oral bioavailability as a percentage (%F) (see Table 5).

As shown in Table 5, all the compounds were predicted endowed with a better lipophilicity and solubility in water than the reference potentiator VX-770, with the exception of the analogue **6**. In addition, among them, the most promising thienopyranes, **11**, **22**–**24**, displayed more favorable plasmatic protein binding values (%PPB = 76.56–88.45) and affinity toward the human serum albumin (logKa^HSA^ = 3.36–3.69) than VX-770 (%PPB = 98.82; logKa^HSA^ = 4.87), turning in higher bioavailability. 

Accordingly, **11**, **22**–**24** (%F = 86.9–99.4) experienced higher oral bioavailability as a percentage than VX-770 (%F = 49.2). For all the compounds, optimal absorption values as HIA have been predicted (HIA = 100%), while three of them, **7**–**9**, proved to be unable to pass the BBB. 

The putative metabolism and toxicity profiles of any compound was determined on the basis of a number of parameters, such as the ability to inhibit the Ether-a-go-go Related-Gene (hERG) potassium channel or to interact with the endocrine system, as well as to behave as cytochrome P450 3A4 and 2D6 inhibitor or substrate (see Table 6). Evaluation of acute toxicity for mice after oral administration (LD_50_) was also estimated in silico.

Based on an overall analysis of the parameters reported in Table 6, none of the compounds explored here should be involved in genotoxicity events such as hERG inhibitory activity or binding to the endocrine system, being accompanied by a high reliability index (RI > 0.40 and RI > 0.50, respectively). Furthermore, none of them proved to inhibit the cytochrome P450 3A4, with all of them being, on the contrary, substrates for this enzyme. 

Finally, all the compounds exhibit a favorable toxicity profile. All the thienopyranes, with the exception of **24**, displayed the estimated LD_50_ values in the range of 790–2700 mg/kg for mice after oral administration, being higher than that of VX-770 (LD_50_ = 780 mg/kg).

## 3. Discussion

Preliminary visual inspection of the X-ray crystallographic data reported by Liu [21] especially suggested a high number of hydrophobic contacts, such as Van der Waals interactions and π–π stacking with residues F236, Y304, F305, S308, A309 and F312, in tandem with H-bonding to S308 and F931. These contacts were detected by the quinolone moiety of the reference drug VX-770 as well as the most promising series of potentiators discussed herein. This binding mode would allow the potentiator to properly arrange within the channel crevice. Refined docking calculations performed herein revealed a key role also played by H-bonding to Y304, F305 and R933, detecting with the last residue not only H-bonds but also cation-π contacts. Exploring the docking mode of different chemotypes enlightened the relevance of the potentiator to be stabilized within the protein cavity by means of hydrophobic contacts or π–π stacking, but also by additional polar interactions involving Y30, S308 and F305.

Notably, H-bonding F931, R933, S308 and Y304 led to derivatives endowed by higher potency as CFTR potentiators, as described for most of the GPLG1837 analogues (series **1**–**26**). The other series of potentiators experienced a lot of these contacts, showing a variable potency ability. More details about the most important contacts exhibited by all the potentiator chemo-types studied herein are summarized in Supplementary Tables S32–S36.

Pharmacophore analyses proved to be in agreement with the results obtained with the aforementioned structure-based studies. Indeed, most of the key features suggested by these analyses promoted the introduction of H-bond acceptor and donor functions onto a planar hydrophobic main core. 

Compound **11** (pEC_50_ = 8.52), chosen as a representative thienopyrane derivative, fulfills these requirements, through the bicyclic ring at F4:AroǀPiNǀHyd and exhibiting the proper H-bonding features F1–F3, thanks to the primary 3-carboxamide substituent onto the main core of the ligand. Beyond these key properties to achieve CFTR potentiator ability, the oxygen atom of the thienopyran ring and the carboxamide moiety linked to the position 2 of the same core made the compound more potent. 

Accordingly, within the CQ series (pEC_50_ = 4.25–6.00), compound **45** (pEC_50_ = 6.00) was the most potent derivative, bearing the piperazine ring in correspondence with the thienopyran ring of **11**, the nitrogen atom of the quinolone core at F3:Acc and the oxygen atom of the methoxy-substituted benzoyl group in proximity of the compound **11** pyran group as F8:Acc (Supplementary Figure S37). 

On the contrary, the analogue **50** (inactive) properly oriented only the quinolone nitrogen atom as F3:Acc.

In accordance with this information, the most effective pyrazolquinolines, **80** (pEC_50_ = 6.52), experienced most of the suggested pharmacophore features, such as the hydrophobic (hetero)aromatic groups reported as F4:AroǀPiNǀHyd, F5:PiN by means of the main tricyclic core and the H-bonding moieties as F1–F3 and F8:Acc through the primary amine group, the nitrogen atoms of the pyrazolyl ring, and by the methoxy substituent onto the quinolone core, respectively (Supplementary Figure S38).

Conversely, the analogue **71** (pEC_50_ = 4.52) maintained only the hydrophobic F4 feature, because of the reversed positioning.

The less-potent tetrahydropyridoindole **57**–**69** (pEC_50_ = 4.52–5.69) and aminoarylthiazole **81**–**88** (pEC_50_ = 4.14–5.19) derivatives satisfied a limited number of pharmacophore requirements compared to the thienopyranes **1**–**26** (pEC_50_ = 6.10–9.26), thus displaying in any case lower potency values as F508del CFTR potentiators. Among them, the AAT **89** (pEC_50_ = 5.71) properly moved the indolone ring in proximity to the reference thienopyrane core as F4:Aro group, properly orienting one of the oxygen atoms of the carbamate moiety in proximity to that of the reference compound pyran ring, as F8:Acc feature (Supplementary Figure S39). In addition, potentiator **89** exhibited the F3:Acc moiety through the nitrogen atom of the thiazole scaffold, being the most promising within this series. Indeed, other thiazole-containing potentiators, such as **82** (pEC_50_ = 4.56) shown in Supplementary Figure S39, only guarantee the H-bonding features F2:Don and F3:Acc thanks to secondary amine group and by the thiazole nitrogen atom. 

Based on these results, the CQ series could be optimized modifying the piperazine ring with a different spacer featuring further H-donor groups (as F2:Don), while the same cyanoquinoline ring could be replaced by a benzimidazole ring as main core, gaining additional H-bonding features in agreement with F7:Don. Concerning the pyrazolquinolines, new analogues featuring an amide function instead of the carbonyl moiety could allow to better mimic the most potent thienopyranes, as well as introducing the isoquinoline moiety. The potency trend of the tetrahydropyridoindoles could be ameliorated, modifying the main core with a pyrazolopyrimidine scaffold, featuring more H-bonding moieties. The introduction of a pyridine ring instead of the thiomethyl phenyl one could guarantee an improvement of the AAT effectiveness, as well as the design and synthesis of analogues bearing a bioisostere replacement of the thiazole core with the imidazole one. On the other hand, the benzoxazolone or indolone groups could be modified into a phthalimide moiety in order to achieve a higher number of polar contacts with the channel.

With the aim at gaining a validation of the whole computational approach applied herein, we proceeded by collecting from the literature and evaluating in silico compounds **91**–**139** (see for the structure Supplementary Tables S40–S42), with only five of them being modest CFTR potentiators (the other ones proved to be completely inactive) [23,25,40]. 

All of them have been submitted to molecular docking calculations based on the same docking protocol previously applied for the potentiators **1**–**90** (Supplementary Table S1–S6) and compared with the aforementioned pharmacophore model. Then, their putative CFTR potentiator ability has been predicted by means of the previously developed QSAR analysis. 

According to our docking calculations, most of the thiazoles **91**–**106** experienced a comparable docking mode which is characterized by two H-bonds contacts involving one oxygen atom of the ester moiety and R933, and a further H-bond between the oxygen of the carboxamide function and F931. Thiazoles **107**–**111** featured only two weak H-bonds with Y304 and F931, lacking any contact with R933. In particular, the di-substitution with electron-withdrawing groups at the R substituent is thought to impair the ability of the aromatic ring to be involved in cation-π contacts with R933. 

It is worth noting that all the most effective potentiators previously explored featured at least two contacts with F931 and another one with Y304 or S308, together with several H-bonds or cation-π contacts with R933. All of them were considered key anchoring points for the potentiator. 

Herein, most of these thiazoles of **91**–**111** were unable to match a high number of contacts with these residues.

Concerning **91**–**106**, the collected poses were quite variable even if maintaining these two interactions, with the exception of the congeners **100** and **101**. Indeed, both of them were better stabilized at the biological target thanks to two H-bonds with R933 and two others with F931. On this basis, only **100** and **101** were retained as putative potentiators, being in agreement with the biological assays reported in the literature [40].

In detail, the docking mode of **100** and **101** shown in Supplementary Figure S43 revealed two H-bonds with R933 and with F931 thanks to the ester moiety and the carboxamide and thiazole nitrogen atom. This positioning allowed the compounds to be efficiently anchored at the binding site, moving the ester chain and the phenyl ring towards the hydrophobic pockets delimited by F931, F932 and L233, F236 and F305, respectively. 

As a consequence, the two hydrophobic cores partially mimicked the quinolone ring and the *tert-*butyl substituent of VX-770, while the tertiary amide chain was involved in Van deer Waals contacts with F931 and F305 (Supplementary Figure S44). 

Compounds **112**–**139** were conceived as hybrid derivatives of the F508del-CFTR corrector VX809, featuring comparable or higher potency ability [25]. Among them, only **127**, **128** and **139** were endowed with a modest CFTR potentiator activity, with all the other analogues being completely inactive. Accordingly, the docking poses obtained for **127** and **128** highlighted two H-bonds and cation-π contacts between the benzodioxole group and R933, one H-bond between the oxygen atom of the carboxamide moiety and F931 and an additional H-bond involving the *m*-OH-phenyl ring and F305 (Supplementary Figure S45). 

The predicted positioning of the hybrid **139** was properly superposed on that of the reference VX-770, maintaining π–π stacking and Van der Waals contacts with Y304, F305, F236, F312, and L233 and A309 respectively, thanks to the **139** quinoline. In addition, the two oxygen atoms of the benzodioxole ring guarantee H-bonds with R933 (Supplementary Figure S46).

By a comparison of compounds **91**–**139** with the previously developed pharmacophore model, **100**, **101**, **127** and **128** partially matched the main required features (Supplementary Figure S47). Indeed, the tertiary amide group and the halogen-substituted-phenyl ring of **100** and **101** satisfied F4:AroǀPiNǀHyd, F5:PiN and F8:Acc, respectively. On the other hand, the carboxamide group falls around the F3:Acc feature. 

Concerning hybrids, **127** moved the hydroxyl group and the methyl substituent in proximity of F8:Acc and F4:AroǀPiNǀHyd, while the carboxamide group mimicked the role played by F7:Don and F3:Acc. More interestingly, the potency trend of **91**–**139** was efficiently predicted by the QSAR model, revealing only for compounds **100**–**101** and **127**, **128** and **139** a modest potentiator activity (predicted pEC_50_ = 4.33–5.31), while for all the other (inactive) compounds, the predicted pEC_50_ range was < 4.24 (Supplementary Table S48).

On this basis, the reliability of the applied docking protocol, pharmacophore analysis and also of the developed QSAR equation was confirmed by explaining the structure–activity relationship within the collected dataset, including the external set of derivatives, properly.

## 4. Materials and Methods

All the compounds were manually built by the ‘MOE Builder’ program and then were parameterized (AM1 partial charges as calculation method) and energy minimized by the ‘Energy Minimize Program’ using MMFF94x forcefield and RMS (root mean square) equal to 0.0001 Kcal/mol/A^2^ of the MOE compute module, to produce a single low-energy conformation for each ligand [30]. 

Molecular docking of all the investigated compounds were carried out within the X-ray structure of human CFTR in complex with the potentiator VX-770 (PDB code = 6O2P) and GLPG1837 (PDB code = 6O1V) [21], which were prepared through the ‘Structure Preparation’ program of the MOE software [30]. 

Docking calculations were done thanks to the LeadIT 2.1.8 software suite (www.biosolveit.com). In particular, the FlexX scoring algorithm is employed by LeadIT 2.1.8, relying on binding free energy calculations taking into account the Gibbs–Helmholtz equation [41,42,43]. The docking approach is focused on a spherical search space which is defined detecting the binding site as a radius of 6 Å far from the co-crystallized ligand. The standard Hybrid Approach (enthalpy and entropy criteria) setting has been chosen as docking strategy, being the related scoring function described in the literature [44]. 

The docking algorithm is a state-of-the-art fragment docker: Ligands are split into so-called fragments, and an initial fragment (or combinations thereof) are placed into multiple places in the pocket and scored using a simple yet very fast pre-scoring scheme. From the *n* solutions placed, the ligand is further built up, fragment by fragment, and the interim solutions are scored against each other. The best scored poses survive the process, and those are delivered to the user. The initial idea relates back to the FlexX algorithm, however, many improvements have been made over the years, such as the “Single Interaction Scan” (SIS) placement that also finds solutions when there are only very few polar groups in a compound. The SIS algorithm uses virtual lines between protein and ligand interaction spots and is trimmed for a very speedy rotation around these lines, thus generating solutions quickly for ligands of more hydrophobic character. The calculated docking poses were ranked by the score values of the lowest energy pose of the derivatives docked to the macromolecule. All compounds were refined and re-ranked by assessment with the algorithm HYDE, belonging to the LeadIT 2.1.8 software [45,46]. 

Taking into account the selected docking poses, a pharmacophore model focused on the most promising series of CFTR potentiators (**1**–**26**, thienopiranes) has been built and then compared with the chemical features experienced by all the other series of less potent potentiators. 

The pharmacophore model has been calculated using the pharmacophore search module implemented in the MOE software. The module pharmacophore consensus of the software MOE generates a set of suggested features based on the proposed alignment of the selected docking conformations. These are characterized by a position, radius and a type expression. The relevance of any feature when based on equal scores is assessed by secondary keys in order: radius, number of molecules, number of conformations, length of the expression and alphabetical sequence.

This kind of analysis is reported in the literature as a successful and well-known approach for drug design (both ligand- and structure-based) as well as for virtual screening. Since several methods for describing pharmacophores could underline significant differences in the detected pharmacophores, it would be useful to rely on molecular docking-based alignments to take into account the interactions with the biological target. In any case, comparing the two approaches could be beneficial for mapping the structural requirements of the ligands. The computational part of pharmacophore modeling has significantly improved during the last years thanks to the availability of software packages, such as MOE software [47,48,49,50]. 

Quantitative Structure–Activity Relationships (QSAR) studies were performed based on calculations of three hundred molecular descriptors, including 2D and 3D parameters, by means of MOE 2019.01 software. 2D molecular descriptors are defined to be numerical properties that can be calculated from the connection table representation of a molecule (e.g., elements, formal charges and bonds, but not atomic coordinates). 2D descriptors are, therefore, not dependent on the conformation of a molecule and are most suitable for large database studies. They include descriptors related to physical properties, subdivided surface areas, atom and bond counts, connectivity-based descriptors, partial charges descriptors, pharmacophore features descriptors and the so-called Adjacency and Distance Matrix Descriptors. The 3D descriptors consist of potential energy descriptors, MOPAC descriptors, Surface Area, Volume and Shape Descriptors and Conformation-Dependent Charge Descriptors.

Afterwards, 306 molecular descriptors (2D and 3D) were computed by MOE and the resulting matrix was submitted to the statistical analyses and (QSAR), objects of the present work. 

A final data matrix of 75 objects (compounds/molecules) and 306 rows (molecular descriptors) was obtained. The chemometric package PARVUS [31] was applied to handle such information, in particular for checking the constant predictors, splitting the data into training and test sets, discarding the non-informative descriptors and extracting meaningful QSAR models able to identify the most important predictors accounting for potentiator pEC_50_ value, as described in detail below.

First of all, the CHECK module implemented in PARVUS was used for checking the constancy of variables in five cancellation groups and 283 molecular descriptors were retained after elimination of the constant predictors. 

Then, the space-filling Kennard-Stone duplex design [35] was used in order to split the data into representative training and test sets. This algorithm was applied using the first 10 principal components of the auto-scaled data, considering 90% of the total variance. Sixty objects were selected for the training set and fifteen molecules were assigned to the test set (20% of the total molecules).

Iterative stepwise elimination PLS (ISEPLS) [51] was applied, as a variable selection method, in order to evaluate the relevance of the selected predictors with regard to the possibility of predicting the response variable y (pEC_50_). ISE is based on the importance of the predictors, defined as:

$$z_{v}=\frac{\left|b_{v}\right|s_{v}}{\sum_{v=1}^{V}\left|b_{v}\right|s_{v}}$$

where b_v_ is the regression coefficient and s_v_ is the standard deviation of the descriptor v. In each elimination cycle, the descriptor with the minimum importance is eliminated, and the model is computed again with the remaining predictors. The final model, that with the maximum predictive ability in cross-validation, retained 20 relevant descriptors.

QSAR was performed by the application of various iterations of partial least-squares (PLS) multivariate analysis by means of MOE software, considering the molecular descriptors as independent variables and corrector pEC_50_ values as the dependent variable. At each iteration, the relative importance of every descriptor in influencing corrector ability was calculated, therefore the less important ones were discarded in the following PLS analysis, until the generation of the final linear regression model. At each PLS, the Leave One Out method was used to check the internal predictability of the derived models. 

The predictive ability of the derived model was evaluated for the test set compounds (expressed as r^2^_pred_), by using the following equation:

r^2^_pred_ = (SD − PRESS)/SD

SD is the sum of the squared deviations between the biological activities of the test set molecules and the mean activity of the training set compounds, and PRESS is the sum of the squared deviation between the observed and the predicted activities of the test set compounds.

The prediction of descriptors related to ADMET properties was developed thanks to the Advanced Chemistry Development (ACD) Percepta platform (www.acdlabs.com). Any ADMET parameter was predicted by Percepta based on the training libraries included in the software, which refer to several series of derivatives whose pharmacokinetic and toxicity profile have been experimentally evaluated.

## 5. Conclusions

Herein, we reported deepened molecular docking studies within the recent X-ray crystallographic data of the CFTR channel in complex with VX-770, considering different series of CFTR potentiators so far described in the literature. The results allowed us to explore the key residues involved in the potentiator binding, such as F931 and R933, via H-bonds and cation-π contacts. Additional polar interactions and H-bonding involving Y304, F305 and S308 as well as π–π stacking with F236, Y304, F931 and F932 stabilized the most potent potentiators at the channel, such as GLPG1837 analogues. This information was in good agreement with the developed pharmacophore analyses and further supported by the developed QSAR model, suggesting the design of novel compounds enriched with H-bonding groups inserted onto a bulky hydrophobic main core. The reliability of the whole computational approach allowed us to set up a robust computational tool to pave the way for a further design of novel drug-like F508del CFTR potentiators. Further work on the new developed series will be disclosed in due course.

## Figures and Tables

**Table 1:** Selected two-dimensional (2D) Subdivided Surface Area ^a^ and Adjacency and Partial Charge descriptors ^b^ and the related relative importance (RI) values based on the developed QSAR model.

Descriptor	Type	Series	RI
SlogP_VSA4	Sum of *v_i_* such that *L_i_* is in (0.1, 0.15)	2D-II	0.293739
SlogP_VSA5	Sum of *v_i_* such that *L_i_* is in (0.15, 0.20)	2D-II	0.501133
SlogP_VSA9	Sum of *v_i_* such that *L_i_* > 0.40	2D-II	0.686395
SMR_VSA2	Sum of *v_i_* such that *R_i_* is in (0.26, 0.35)	2D-II	0.429419
SMR_VSA4	Sum of *v_i_* such that *R_i_* is in (0.39, 0.44)	2D-II	0.234752
PEOE_VSA+5	Sum of *v_i_* where *q_i_* is in the range (0.25, 0.30)	2D-V	0.053586
PEOE_VSA-6	Sum of *v_i_* where *q_i_* is less than −0.30	2D-V	0.461873

^a^ The Subdivided Surface Areas are descriptors based on an approximate accessible van der Waals surface area (in Å^2^) calculation for each atom, *v_i_*, along with some other atomic property, *p_i_*. The *v_i_* are calculated using a connection table approximation. Each descriptor in a series is defined to be the sum of the *v_i_* over all atoms *i,* such that *p_i_* is in a specified range (*a*, *b*). *L*_i_ denotes the contribution to logP(*o*/*w*) for atom *i* as calculated in the SlogP descriptor [32]. *R_i_* denotes the contribution to Molar Refractivity for atom _i_ as calculated in the SMR descriptor [33]. The ranges were determined by percentile subdivision over a large collection of compounds. ^b^ Let *q_i_* denote the partial charge of atom _i_. Let *v_i_* be the van der Waals surface area (Å^2^) of atom *i* (as calculated by a connection table approximation).

**Table 2:** Selected 2D Adjacency and Distance Matrix Descriptors. ^a^ Pharmacophore Feature Descriptors, Physical Properties and Atom Counts and Bond Counts descriptors. The related relative importance (RI) values based on the developed QSAR model are shown.

Descriptor	Type	Series	RI
BCUT_SLOGP_1	The BCUT descriptors using atomic contribution to logP	2D-VII	0.578303
BCUT_SMR_1	The BCUT descriptors using atomic contribution to molar refractivity	2D-VII	0.500605
BCUT_SMR_2	The BCUT descriptors using atomic contribution to molar refractivity	2D-VII	0.029230
BCUT_SMR_3	The BCUT descriptors using atomic contribution to molar refractivity	2D-VII	0.720350
a_hyd	Number of hydrophobic atoms	2D-VI	0.101682
LogS	Log of the aqueous solubility (mol/L). This property is calculated from an atom contribution linear atom type model with r^2^ = 0.90, ~1200 molecules [33]	2D-I	0.950723
b_1rotR	Fraction of rotatable single bonds: number of rotatable bonds (b_1rotN) divided by number of bonds between heavy atoms (b_heavy)	2D-III	0.211130

^a^ The (aka Burden Eigenvalues) BCUT descriptors [34] are calculated from the eigenvalues of a modified adjacency matrix. Each *ij* entry of the adjacency matrix takes the value 1/sqrt(*bij*), where *bij* is the formal bond order between bonded atoms *i* and *j*. For the reported descriptors, the diagonal takes the value of the LogP or molar refractivity. The resulting eigenvalues are sorted and the smallest, 1/3-ile, 2/3-ile, and largest eigenvalues are reported.

**Table 3:** Selected three-dimensional (3D) Surface Area, Volume and Shape Descriptors and Conformation-Dependent Charge Descriptors and the related relative importance (RI) values based on the developed QSAR model.

Descriptor	Type	Series	RI
vsurf_ID1	Hydrophobic integy moment	3D-III	0.119071
vsurf_ID7	Hydrophobic integy moment	3D-III	0.582277
vsurf_Wp2	Polar volume	3D-III	0.243649
vsurf_Wp3	Polar volume	3D-III	1.000000
dipoleY	The *y* component of the dipole moment	3D-V	0.492821
ASA^+^	Water-accessible surface area of all atoms with positive partial charge	3D-V	0.309621

**Table 4:** List of the mean values of the most relevant 3D descriptors (RI > 0.5) and of 2D descriptors chosen as reference parameters within the different 2D subtypes. All of them have been calculated based on the several chemotypes explored herein.

Potentiator Series	b_1rotR_mean_	a_hyd_mean_	Vsurf_WP3_mean_	Vsurf_ID7_mean_	SlogP_VSA9_mean_	pEC_50_
Thienopyrans	0.11934	15.8667	183.5333	1.4341	178.6650	6.10–9.26
Cyanoquinolines	0.1830	19.0741	118.6481	1.1386	68.4367	4.25–6.00
Indole-based derivatives	0.0901	21.8461	117.8750	1.09374	26.9052	4.52–5.69
Pyrazoloquinolines	0.1313	17.1000	160.6875	0.9737	40.7683	4.52–6.52
Amino aryl Thiazoles	0.17156	17.5454	108.8875	0.98697	96.0631	4.14–5.71

**Table 5:** Calculated ADME descriptors related to absorption and distribution properties. The most promising GLPG1837 analogues, **11**, **22**–**24**, have been highlighted in cyan, while the reference compounds VX-770 are reported in yellow. The only derivatives predicted as insoluble in water or unable to pass the blood–brain barrier (BBB) are shown in italic and colored in light grey.

Comp.	cLogP	LogBB ^a^	LogPS ^b^	HIA (%) ^c^	Vd (l/kg) ^d^	%PPB	LogKa ^HSA^	%F (Oral)	Solubility (mg/mL)
**1**	2.98	0.18	−1.4	100	2.1	93.86	3.86	95.0	0.10
**3**	2.56	−0.19	−1.8	100	1.3	92.00	3.72	99.2	0.25
**4**	2.93	0.02	−1.6	100	1.5	93.70	3.99	94.6	0.14
***6***	3.46	0.29	−1.4	100	1.7	95.20	4.20	83.2	*0.07*
***7***	2.90	*−0.00*	*−2.6*	100	1.5	94.37	4.18	97.9	0.41
***8***	1.55	*−0.54*	*−3.7*	100	0.29	91.58	4.65	96.7	0.92
***9***	0.88	*−0.63*	*−2.8*	100	0.32	84.39	3.59	98.4	0.75
**10**	2.16	0.17	−2.2	100	1.3	77.92	3.36	99.4	0.57
**11**	2.16	0.18	−2.2	100	1.3	77.92	3.36	99.4	0.57
**12**	2.29	0.06	−2.3	100	1.2	77.50	3.41	99.4	0.47
**22**	2.71	0.26	−1.9	100	1.2	88.45	3.69	99.0	0.26
**23**	1.78	0.25	−2.2	100	1.4	76.56	3.38	98.6	0.34
**24**	2.33	0.50	−2.2	100	1.5	85.40	3.47	86.9	0.31
**25**	1.78	0.25	−2.2	100	1.4	76.56	3.38	86.9	0.34
**26**	2.54	0.41	−1.8	100	1.3	87.76	3.72	94.0	0.14
**VX-770**	4.84	0.29	−1.3	100	3.6	98.82	4.87	49.2	0.09

^a^ Extent of brain penetration based on ratio of total drug concentrations in tissue and plasma at steady-state conditions. ^b^ Rate of brain penetration. PS represents Permeability-Surface area product and is derived from the kinetic equation of capillary transport. ^c^ HIA represents the human intestinal absorption, expressed as percentage of the molecule able to pass through the intestinal membrane. ^d^ prediction of Volume of Distribution (Vd) of the compound in the body.

**Table 6:** Calculated toxicity descriptors related to metabolism, excretion and toxicity properties for the GLPG1837 analogues **1**, **3**, **4**, **6**–**12**, **22**–**26** and for the reference potentiator VX-770. The most promising GLPG1837 analogues, **11**, **22**–**24**, have been highlighted in cyan, while the reference compounds VX-770 are reported in yellow.

Compound	LD_50_^a^ (mg/kg) (RI ≥ 0.30) Mouse, Oral	hERG Inhibitor (RI ≥ 0.40)	Endocrine System Disruption ^b^ (RI ≥ 0.50)		CYP3A4 and CYP2D6 (RI ≥ 0.40)	
			LogRBA > −3	LogRBA > 0	Inhibitor < 10 mM	Substrate
**1**	1100	No inhibitor	No binder	No binder	0.02	CYP3A4
**3**	1000	No inhibitor	No binder	No binder	0.01	CYP3A4
**4**	840	No inhibitor	No binder	No binder	0.01	CYP3A4
**6**	820	No inhibitor	No binder	No binder	0.01	CYP3A4
**7**	790	No inhibitor	No binder	No binder	0.01	CYP3A4
**8**	1500	No inhibitor	No binder	No binder	0.01	CYP3A4
**9**	2700	No inhibitor	No binder	No binder	0.02	CYP3A4
**10**	1200	No inhibitor	No binder	No binder	0.02	CYP3A4
**11**	1200	No inhibitor	No binder	No binder	0.02	CYP3A4
**12**	1000	No inhibitor	No binder	No binder	0.01	CYP3A4
**22**	1000	No inhibitor	No binder	No binder	0.01	CYP3A4
**23**	1000	No inhibitor	No binder	No binder	0.02	CYP3A4
**24**	510	No inhibitor	No binder	No binder	0.01	CYP3A4
**25**	1000	No inhibitor	No binder	No binder	0.01	CYP3A4
**26**	1000	No inhibitor	No binder	No binder	0.01	CYP3A4
**VX-770**	780	No inhibitor	No binder	No binder	0.01	CYP3A4

^a^ Acute toxicity by the median lethal dose (LD_50_) for mouse after oral administration. ^b^ RBA represents the relative binding affinity with respect to that of estradiol. Compounds showing LogRBA > 0 are classified as strong estrogen binders, while those showing LogRBA < −3 are considered as non-binders. (RI: reliability index. Borderline-allowed values for reliability parameter are ≥ 0.3, the most predictive fall in the range 0.50–1.0).

## Supplementary Materials

Supplementary Materials can be found at https://www.mdpi.com/1424-8247/13/12/445/s1.

## Abbreviations

AAT	aminoarylthiazole
CF	cystic fibrosis
CFTR	cystic fibrosis transmembrane conductance regulator
F508del	deletion of phenylalanine at position 508
PDB	protein data bank
pEC_50_	negative logarithm of the half maximal effective concentration (EC50)
