# Supplementary material for: New Insights into the Binding Features of F508del CFTR Potentiators: A Molecular Docking, Pharmacophore Mapping and QSAR Analysis Approach
Source: Pharmaceuticals (Basel). 2020 Dec 4;13(12):445. doi: 10.3390/ph13120445 (PMC7762081; doi:10.3390/ph13120445)
Supplement: Supplementary file 1 [file pharmaceuticals-13-00445-s001.pdf]

SUPPLEMENTARY MATERIALS

New insights into the binding features of F508del CFTR potentiators: a molecular docking, pharmacophore mapping and QSAR analysis approach

Giada Righetti[1], Monica Casale[2], Michele Tonelli[1], Nara Liessi[3,4], Paola Fossa[1*], Nicoletta Pedemonte[5], Enrico Millo[3,4] and Elena Cichero[1*]

S1. Chemical structures and potency profile of thienopyrazole derivatives **1- 26** as F508del CFTR potentiators [27]. Activity of the compounds was explored by YFP Halide Assay and TECC Experiments[a].

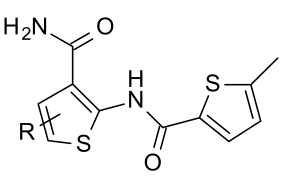

1-3

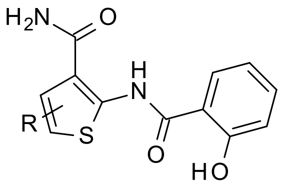

4-6

Compound	R	pEC$_{50}$ M
**1**	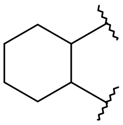	6.18
**2**	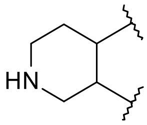	n.a
**3**	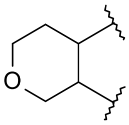	6.10
**4**	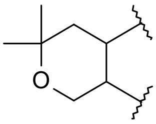	7.88
**5**	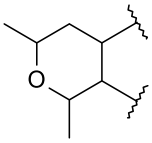	8.39
**6**	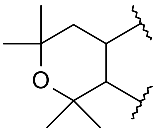	8.39

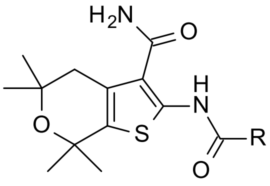

7-26

**Cp.**	**R**	**pEC$_{50}$ M**
**7**	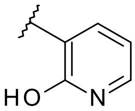	6.72
**8**	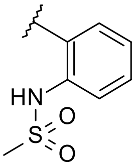	6.83
**9**	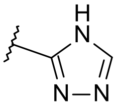	6.33
**10**	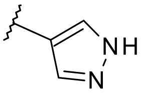	6.90
**11**	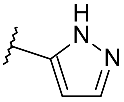	8.52
**12**	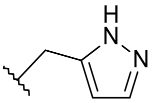	6.86
**13**	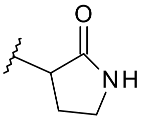	6.99
**14**	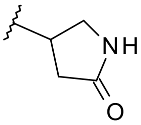	>10,000
**15**	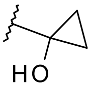	7.63
**16**	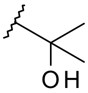	7.79
**17**	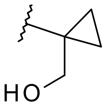	7.82

**18**	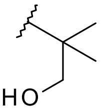	7.12
**19**	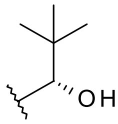	8.56
**20**	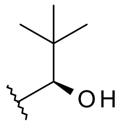	6.89
**21**	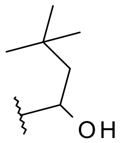	9.26
**22**	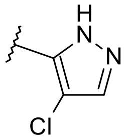	8.69
**23**	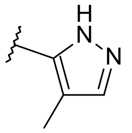	8.69
**24**	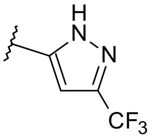	8.52
**25**	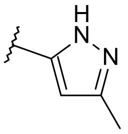	7.88
**26**	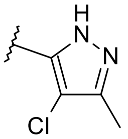	7.95

[a] A bronchial epithelial cell line derived from a CF patient (CFBE41o− cells) was used to overexpress F508delCFTR and yellow fluorescent protein. CFBe41o-cells were cultured in Eagle's Minimal Essential Medium (MEM) (Life Technologies) supplemented with 10% fetal bovine serum (FBS), 1% penicillin/streptomycin, 1% L-glutamine, and 500µg/mL hygromycin B. The cells were grown on culture flasks coated with 0.01% bovine serum albumin (BSA) (Sigma), 30µg/mL Purecol (Nutacon), and 0.001% CFBe41o-human fibronectin (Sigma). CFBe41o-cells were transduced with adenoviruses containing F508delCFTRand YFP (H148Q/I152L/F47L). Then, CFBE41o− cells were incubated for 24 h at 27 °C and treated for 10 min with 10 µM forskolin and the desired concentration of potentiator at room temperature. The YFP fluorescence was recorded during 7 s (CFBE41o−) starting immediately before addition of NaI buffer to the wells, using a fluorescence reader. Transepithelial clamp circuit (TECC) recordings were performed using the TECC instrument developed and sold by EP Design (Bertem, Belgium). For acute potentiator experiments, compounds were added on both sides to test their potential for increasing CFTR gating. Measurements were done during a 20min time frame with recordings every 2min. The transepithelial potential (PD) and transepithelial resistance

(Rt) were measured in an open circuit and transformed to I_{eq} using Ohm's law. The increase in I_{eq} (ΔI) was used as a measure for the increased CFTR activity.The capacity of potentiator to increase CFTR channel function was expressed as 1-(fluorescence after NaI addition (F)/fluorescence before NaI addition (F0)).

S2. Chemical structures and potency profile of cyanoquinolines **27- 32** as F508del CFTR potentiators [28]. Potentiator activity is assayed in low-temperature rescued F508del-CFTR-expressing cells in which the protein is targeted to the plasma membrane by 12-h incubation at reduced temperature, and test compound (together with cAMP agonist) is added just before or at the time of fluorescence or electrophysiological assay.[a.]

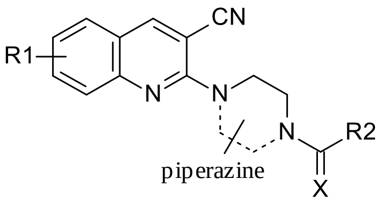

Compound	Structure	pEC50 M
**27**	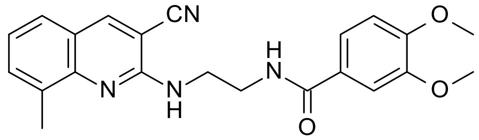	4.82
**28**	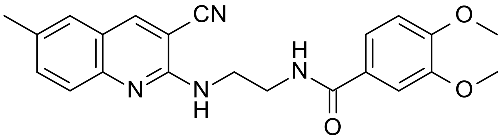	4.82
**29**	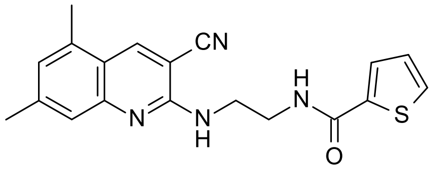	4.85
**30**	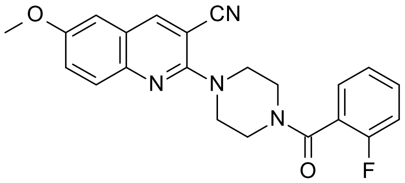	4.95
**31**	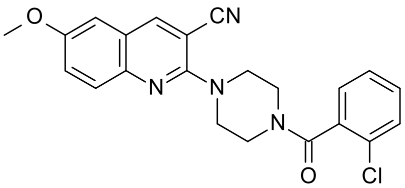	5.33
**32**	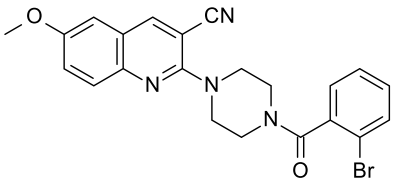	5.30

[a] Fisher rat thyroid (FRT) epithelial cells were stably transfected with F508del-CFTR. The CFTR-expressing cell lines was also transfected with halide-sensitive green fluorescent protein YFP-H148Q/I152L/F46L. For

potentiator assay, FRT cells were grown at 37°C/5% CO2 for 18 to 24 h and then for 18 to 24 h at 27°C. At the time of the assay, cells were washed with PBS and then incubated for 10 min with PBS (50 μl) containing forskolin (20 μM) and test compound (0–25 μM final concentration). each well was assayed individually for I^- influx by recording fluorescence continuously (200 ms per point) for 2 s (baseline) and then for 12 s after rapid addition of 165 μl of PBS, in which 137 μM Cl^- was replaced by I^-. All compound plates contained negative controls (DMSO vehicle) and positive controls (50 μM genistein for potentiator assay).

S3. Chemical structures and potency profile of cyanoquinolines **33-56** as F508del CFTR potentiators[a] [29].

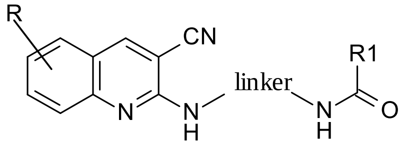

Compound	Structure	pEC_{50} M
**33**	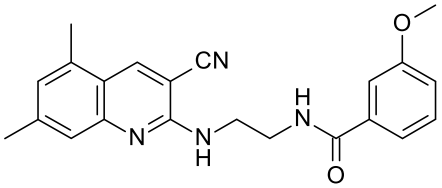	5.22
**34**	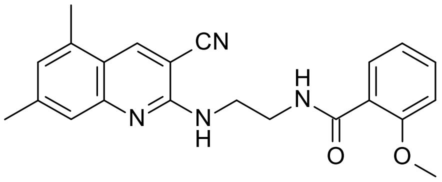	5.63
**35**	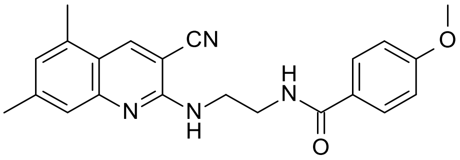	5.38
**36**	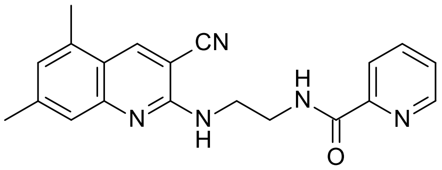	5.92
**37**	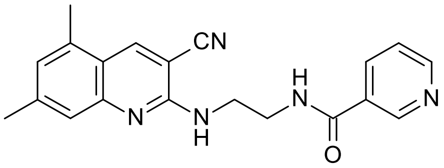	4.87
**38**	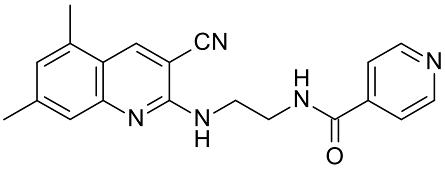	5.00

**39**	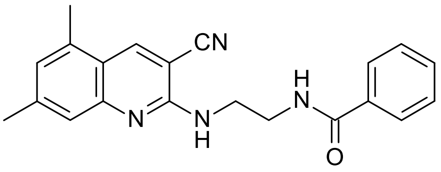	5.00
**40**	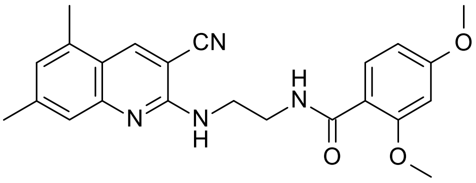	4.25
**41**	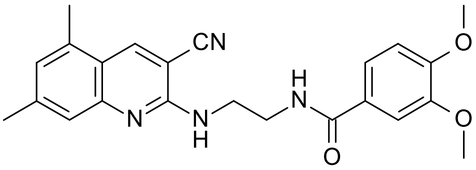	4.31
**42**	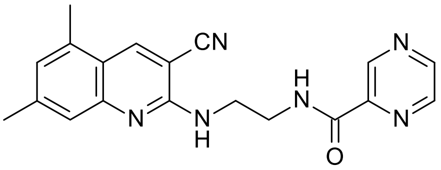	n.a
**43**	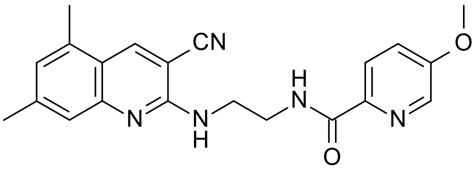	5.46
**44**	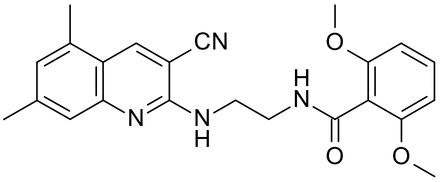	4.93
**45**	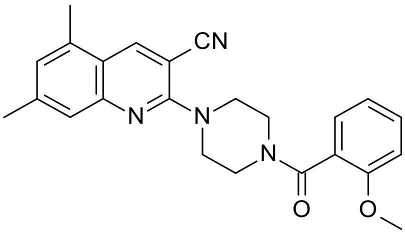	6.00
**46**	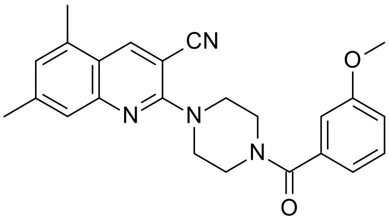	5.92

**47**	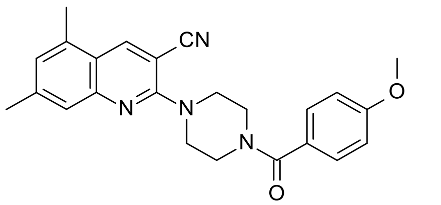	5.88
**48**	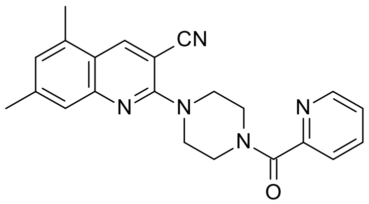	5.13
**49**	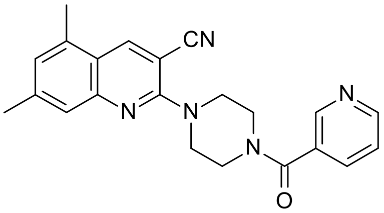	n.a
**50**	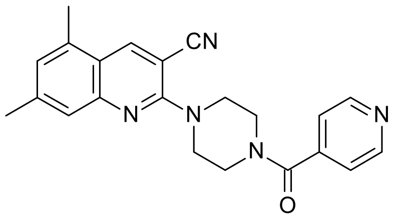	n.a
**51**	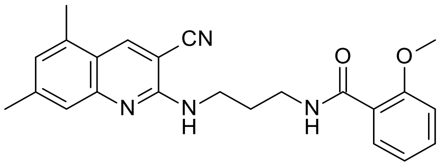	4.55
**52**	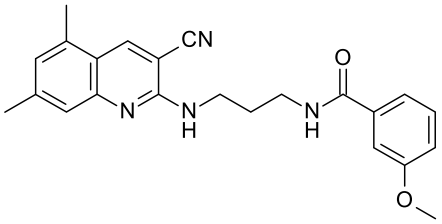	5.33
**53**	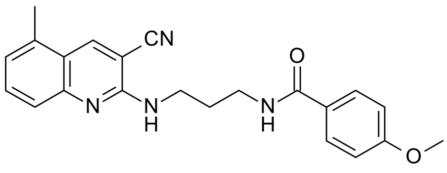	5.30
**54**	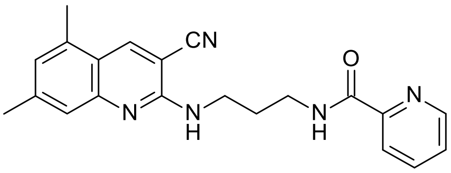	5.21

**55**	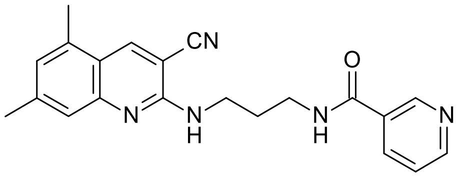	4.79
**56**	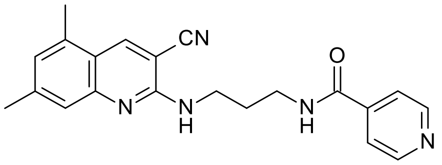	4.87

[a] For potentiator assay, cells were grown at 37 °C for 18–24 h and then for 18–24 h at 27 °C. At the time of the assay, cells were washed with PBS and then incubated for 10 min with PBS (50 μL) containing forskolin (20 μM) and test compound (0–50 μM final concentration). Measurements were carried out using FLUOstar fluorescence plate readers (Optima, BMG LABTECH Gmbh). Each well was assayed individually for I− influx by recording fluorescence for 2 s (baseline) and then for 12 s after rapid addition of 165 μL of PBS in which 137 mM Cl− was replaced by I−. I− influx rate was computed by exponential regression. All experiments contained negative control (DMSO vehicle) and positive controls (potentiator assay, genistein). EC_{50} and Vmax, and their associated uncertainties, were determined by 4 parameter logistic nonlinear regression from concentration-activity data using GraphPad Prism Version 5.01.

S4. Chemical structures and potency profile of piperidine-pyridoindole analogs **57-69** as F508del CFTR potentiators[a] [26].

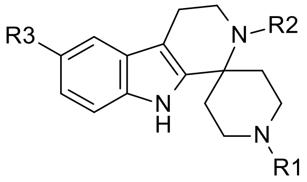

**Compound**	**R_1**	**R_2**	**R_3**	**pEC_{50} M**
**57**	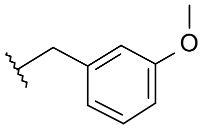	H	OMe	5.00
**58**	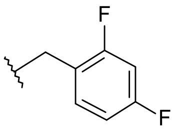	H	OMe	5.69
**59**	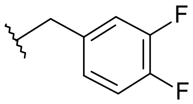	H	OMe	5.58
**60**	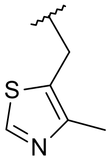	H	OMe	5.29
**61**	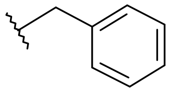	H	H	4.79

**62**	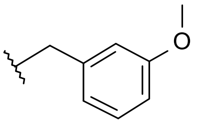	H	H	4.88
**63**	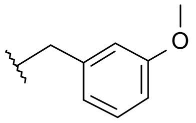	Me	H	< 4.52
**64**	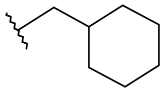	H	OMe	< 4.52
**65**	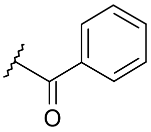	H	H	< 4.52
**66**	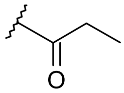	H	OMe	< 4.52
**67**	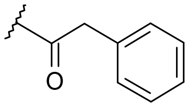	H	OMe	< 4.52
**68**	SO_2Me	H	OMe	< 4.52
**69**	Me	H	OMe	< 4.52

[a] Screening of potentiators was done using FRT cells stably expressing different mutant of the CFTR protein and the halide-sensitive EYFP-H148Q/I152L/F46L (YFP) that were treated for 24 hours with 3 μM VX-809 to increase CFTR1281 cell surface expression. Just prior to assay cells were treated for 10 min with test compounds at 25 μM together with 20 μM forskolin and 15 nM VX-770. CFTR channel activity was deduced from the initial rate of YFP fluorescence quenching in response to addition of iodide-substituted phosphate buffered saline. After initial confirmation with plate reader assays, short-circuit current measurement revealed the most promising derivatives. .

S5. Chemical structures and chemical compounds of pyrazoloquinolines analogs **70-80** as F508del CFTR potentiators[a] [26].

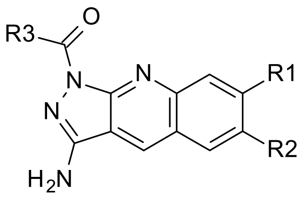

**Compound**	**R_1**	**R_2**	**R_3**	**pEC_{50} M**
**70**	OMe	H	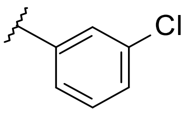	4.82
**71**	OMe	H	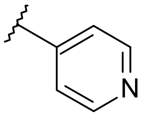	< 4.52

**72**	OMe	H	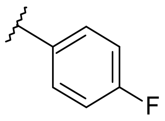	5.44
**73**	OMe	H	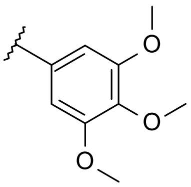	< 4.52
**74**	OMe	H	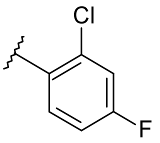	5.61
**75**	OMe	H	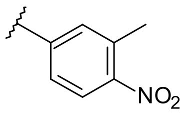	< 4.52
**76**	OMe	H	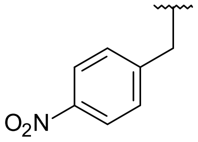	< 4.52
**77**	OMe	H	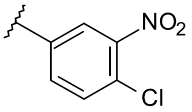	< 4.52
**78**	OMe	H	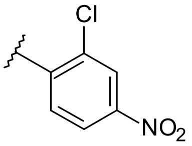	5.76
**79**	OMe	H	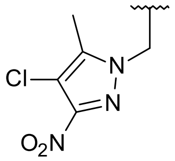	< 4.52
**80**	OMe	H	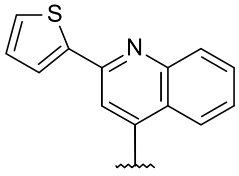	6.52

[a] Screening of potentiators was done using FRT cells stably expressing different mutant of the CFTR protein and the halide-sensitive EYFP-H148Q/I152L/F46L (YFP) that were treated for 24 hours with 3 μM VX-809 to increase CFTR1281 cell surface expression. Just prior to assay cells were treated for 10 min with test compounds at 25 μM together with 20 μM forskolin and 15 nM VX-770. CFTR channel activity was deduced from the initial rate of YFP fluorescence quenching in response to addition of iodide-substituted phosphate buffered saline. After initial confirmation with plate reader assays, short-circuit current measurement revealed the most promising derivatives. .

S6. Chemical structures and potency profile of aminoarylthiazoles **81-88** as F508del CFTR potentiators[a] [22-23].

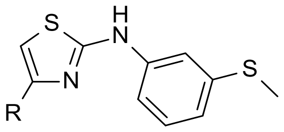

Compound	R	pEC$_{50}$ M
**81**	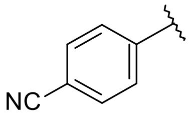	4.46
**82**	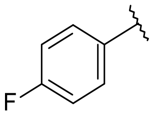	4.56
**83**	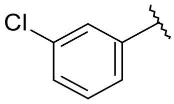	4.27
**84**	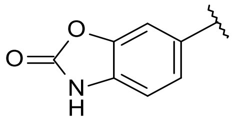	5.19
**85**	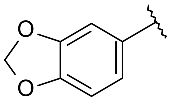	4.14
**86**	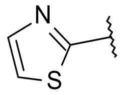	5.00
**87**	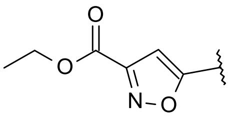	4.23
**88**	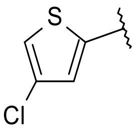	4.49
**89**	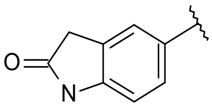	5.71
**90**	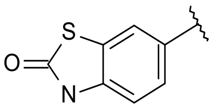	5.20

[a] For determination of potentiator activity on F508del-CFTR, CFBE41o-cells were incubated for 24 h at 27 °C to allow trafficking of the mutant protein to plasma membrane. Cells were then stimulated with for 30 min with PBS containing forskolin (20 µM) plus the compound to be tested at the desired concentration. For determination of potentiator activity on G551D-CFTR, FRT cells were directly stimulated with forskolin plus compound cocktail without previous incubation at low temperature.

S7. Superimposition of the X-ray crystallographic data of CFTR in complex with **VX-770** (pdb code = 6O2P; ribbon in light blue.) and with **GLPG1837** (pdb code = 6O1V; ribbon in pink.). [21]

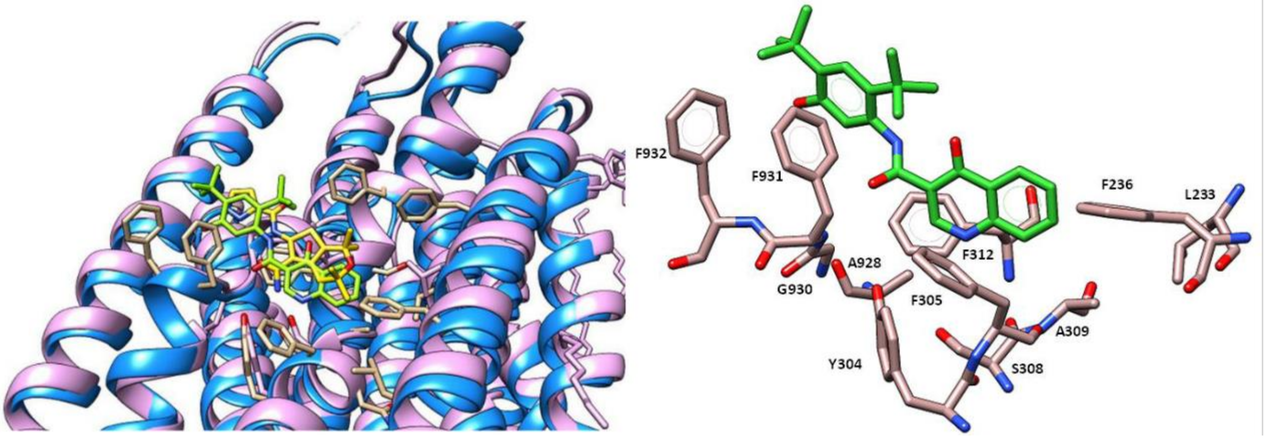

S8. Predicted binding affinity values and related scoring functioning obtained by molecular docking studies of the compounds VX-770 and GLPG-1837 by means of LeadIT software.

hCFTR-Potentiator Complex (LeadIT)	Binding Affinity Energy ΔG (kJ/mol)	Score
**hCFTR-VX-770**	-20.0	-24.5839
**hCFTR-GLPG1837**	-13.0	-25.0793

S9. Predicted binding affinity values and related scoring functioning obtained by molecular docking studies of thienopyrane derivatives **1-26** by means of LeadIT software.

.

hCFTR-Potentiator Complex (LeadIT)	Binding Affinity Energy ΔG (kJ/mol)	Score
**hCFTR-1**	-27.0	-22.8042
**hCFTR-2**	-24.0	-22.0944
**hCFTR-3**	-24.0	-21.9986
**hCFTR-4**	-16.0	-20.2080
**hCFTR-5**	-13.0	-18.2435
**hCFTR-6**	-18.0	-22.5589
**hCFTR-7**	-16.0	-23.8179
**hCFTR-8**	-15.0	-23.7156
**hCFTR-9**	-13.0	-20.2545
**hCFTR-10**	-17.0	-24.3329
**hCFTR-11**	-19.0	-23.3311
**hCFTR-12**	-17.0	-20.1142
**hCFTR-13**	-15.0	-21.3397
**hCFTR-14**	-17.0	-22.2725
**hCFTR-15**	-16.0	-18.5975
**hCFTR-16**	-18.0	-20.5363
**hCFTR-17**	-19.0	-18.0243
**hCFTR-18**	-18.0	-17.6943
**hCFTR-19**	-20.0	-19.9937
**hCFTR-20**	-18.0	-19.1455
**hCFTR-21**	-18.0	-17.8490
**hCFTR-22**	-16.0	-25.0372
**hCFTR-23**	-17.0	-24.6190
**hCFTR-24**	-18.0	-20.7271
**hCFTR-25**	-16.0	-25.9844
**hCFTR-26**	-16.0	-23.8951

S10. Predicted binding affinity values and related scoring functioning obtained by molecular docking studies of the cyanoquinoline derivatives **27-56** by means of LeadIT software.

hCFTR-Potentiator Complex (LeadIT)	Binding Affinity Energy ΔG (kJ/mol)	Score
**hCFTR-27**	-17.0	-16.9254
**hCFTR-28**	-13.0	-13.3363
**hCFTR-29**	-10.0	-17.2431
**hCFTR-30**	-13.0	-17.9474
**hCFTR-31**	-19.0	-17.1064
**hCFTR-32**	-11.0	-15.8932
**hCFTR-33**	-16.0	-16.1000
**hCFTR-34**	-13.0	-15.9863
**hCFTR-35**	-9.0	-16.2100
**hCFTR-36**	-21.0	-16.5779
**hCFTR-37**	-12.0	-17.2708
**hCFTR-38**	-21.0	-15.9125
**hCFTR-39**	-24.0	-16.7263
**hCFTR-40**	-17.0	-12.1516
**hCFTR-41**	-11.0	-10.7610
**hCFTR-42**	-14.0	-15.3367
**hCFTR-43**	-20.0	-14.4144
**hCFTR-44**	-9.0	-10.5040
**hCFTR-45**	-22.0	-20.1247
**hCFTR-46**	-17.0	-20.9311
**hCFTR-47**	-23.0	-19.7379
**hCFTR-48**	-19.0	-18.2752
**hCFTR-49**	-9.0	-18.6320
**hCFTR-**	-21.0	-19.5051

S11. Predicted binding affinity values and related scoring

**50**		
**hCFTR-51**	-13.0	-15.0328
**hCFTR-52**	-18.0	-14.8880
**hCFTR-53**	-11.0	-12.3257
**hCFTR-54**	-10.0	-14.7449
**hCFTR-55**	-21.0	-15.4514
**hCFTR-56**	-28.0	-14.9877

functioning obtained by molecular docking studies of the piperidine-pyridoindole derivatives **57-69** by means of LeadIT software.

.

**hCFTR-Potentiator Complex (LeadIT)**	**Binding Affinity Energy ΔG (kJ/mol)**	**Score**
**hCFTR-57**	-28.0	-13.5717
**hCFTR-58**	-25.0	-14.9293
**hCFTR-59**	-33.0	-13.2128
**hCFTR-60**	-31.0	-13.6552
**hCFTR-61**	-31.0	-12.4418
**hCFTR-62**	-34.0	-12.9565
**hCFTR-63**	-16.0	-9.9620
**hCFTR-64**	-36.0	-15.5064
**hCFTR-65**	-16.0	-14.9051
**hCFTR-66**	-32.0	-15.1299
**hCFTR-67**	-33.0	-15.3735
**hCFTR-68**	-30.0	-14.7068
**hCFTR-69**	-29.0	-14.0463

S12. Predicted binding affinity values and related scoring functioning obtained by molecular docking studies of the pyrazoloquinolines derivatives **70-80** by means of LeadIT software.

hCFTR-Potentiator Complex (LeadIT)	Binding Affinity Energy ΔG (kJ/mol)	Score
**hCFTR-70**	-21.0	-23.6952
**hCFTR-71**	-20.0	-24.9928
**hCFTR-72**	-10.0	-22.0682
**hCFTR-73**	-21.0	-18.2053
**hCFTR-74**	-22.0	-23.3145
**hCFTR-75**	-19.0	-23.1269
**hCFTR-76**	-24.0	-24.4577
**hCFTR-77**	-12.0	-21.6711
**hCFTR-78**	-11.0	-22.3452
**hCFTR-79**	-8.0	-25.5199
**hCFTR-80**	-12.0	-21.1932

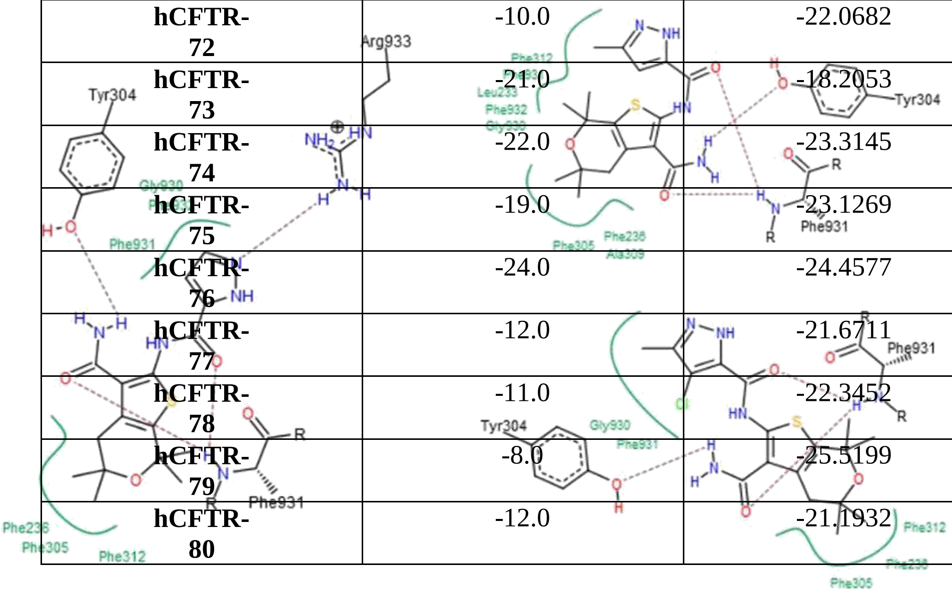

S13. Predicted binding affinity values and related scoring functioning obtained by molecular docking studies of the AATs **81-90** by means of LeadIT software.

hCFTR-Potentiator Complex (LeadIT)	Binding Affinity Energy ΔG (kJ/mol)	Score
**hCFTR-81**	-17.0	-16.2965
**hCFTR-82**	-24.0	-21.7077
**hCFTR-83**	-24.0	-22.0010
**hCFTR-84**	-23.0	-20.1156
**hCFTR-85**	-25.0	-21.6964
**hCFTR-86**	-20.0	-19.0612
**hCFTR-87**	-29.0	-13.0829
**hCFTR-88**	-22.0	-19.0818
**hCFTR-89**	-21.0	-17.6904
**hCFTR-90**	-24.0	-19.1025

S14. Ligplot of the derived docking poses of compound **11** (left) and **22**, **23** (right) at the CFTR cavity.

S15. Ligplot of the derived docking poses of compound **11** (left) and **10**, **12** at the CFTR cavity.

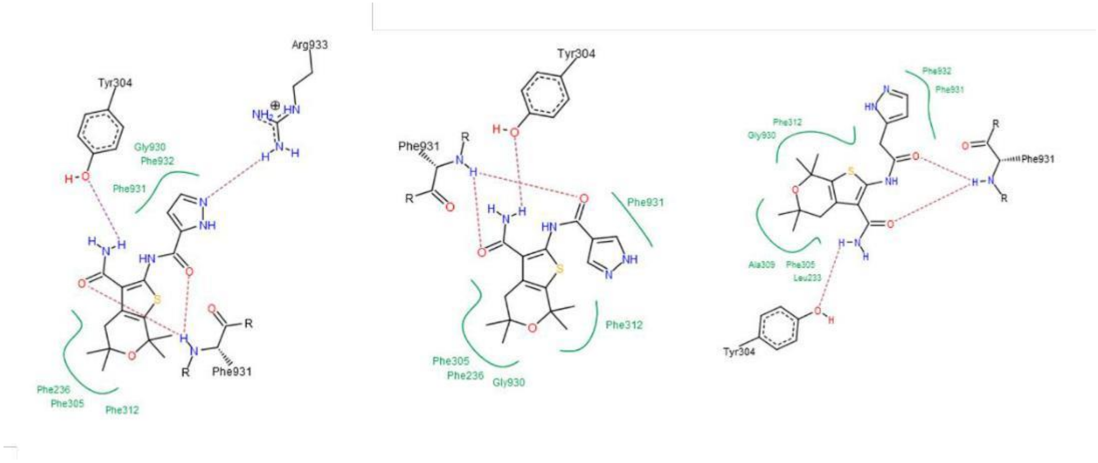

S16. Ligplot of the derived docking poses of compound **9** *(left)* and **12** ***(****right)* at the CFTR cavity.

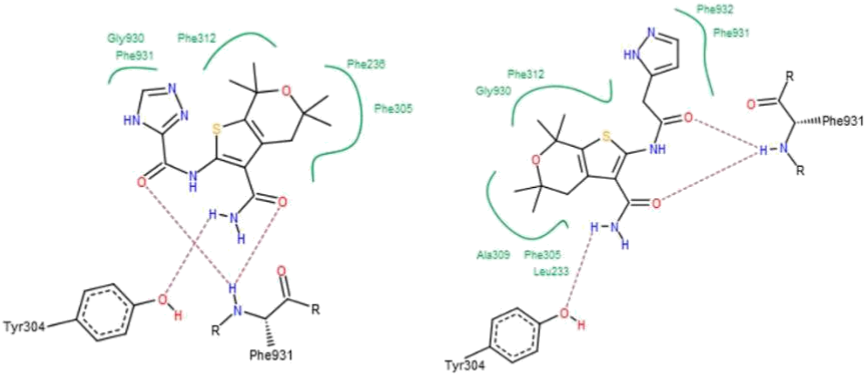

S17. Docking positioning of compound **19** (*left*; *C* atom in deep pink) and of compound **20** (*right*; *C* atom in green) within the human CFTR protein.

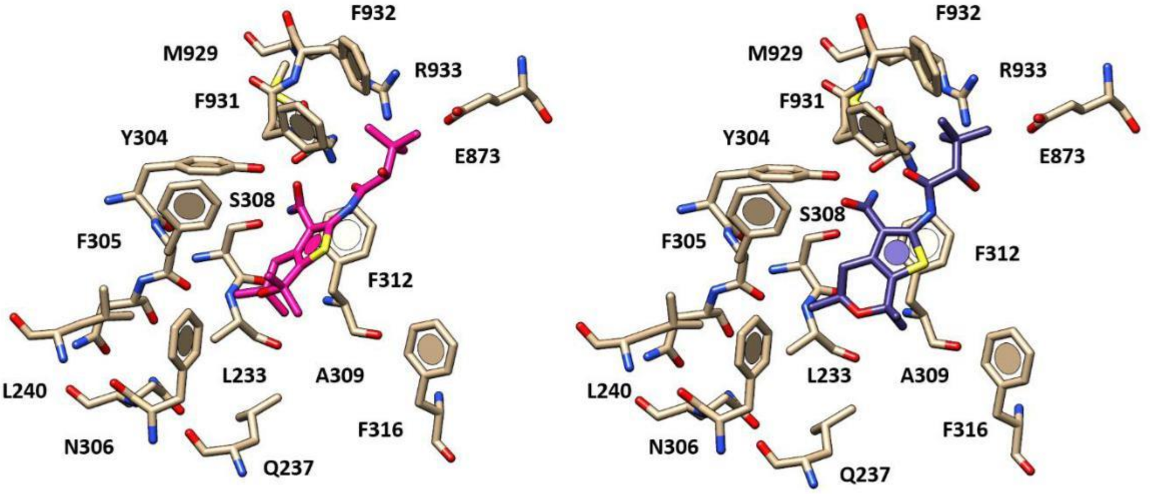

S18. Docking positioning of the compound **21** *S* enantiomer (*C* atom; yellow) within the human CFTR protein.

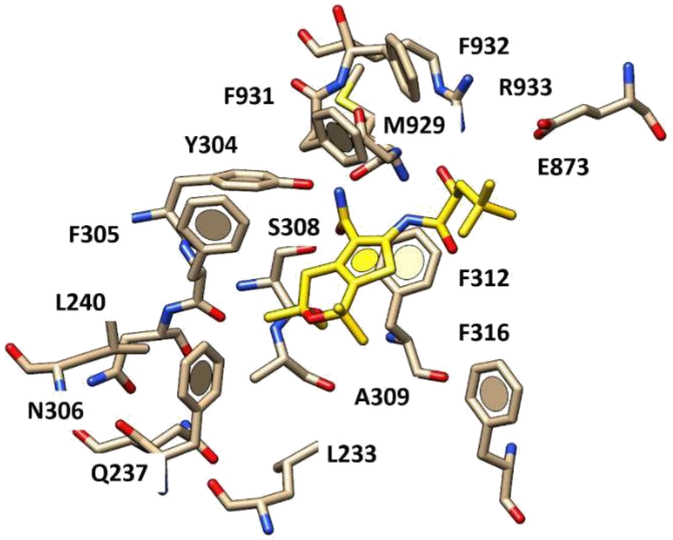

S19. Ligplot of the derived docking poses of compound **45** *(left)* and **48** *(right)* at the CFTR cavity.

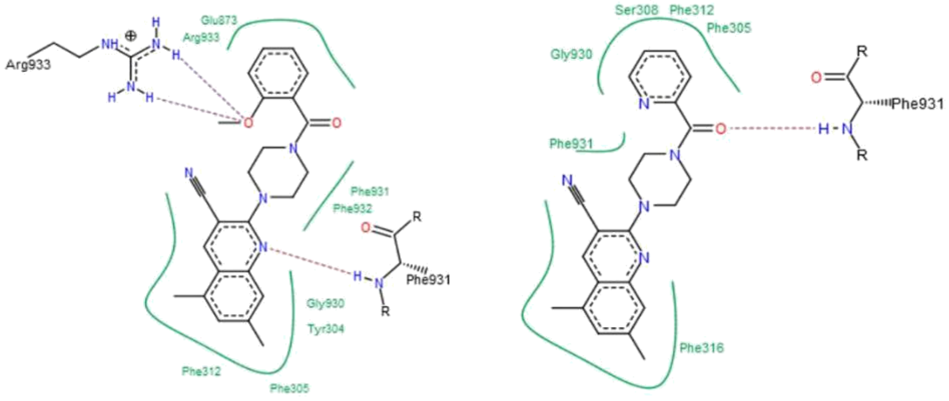

S20. Docking positioning of compound **34** (*C* atom; pink) compared to that of compound **45** (*C* atom; light green) within the human CFTR protein.

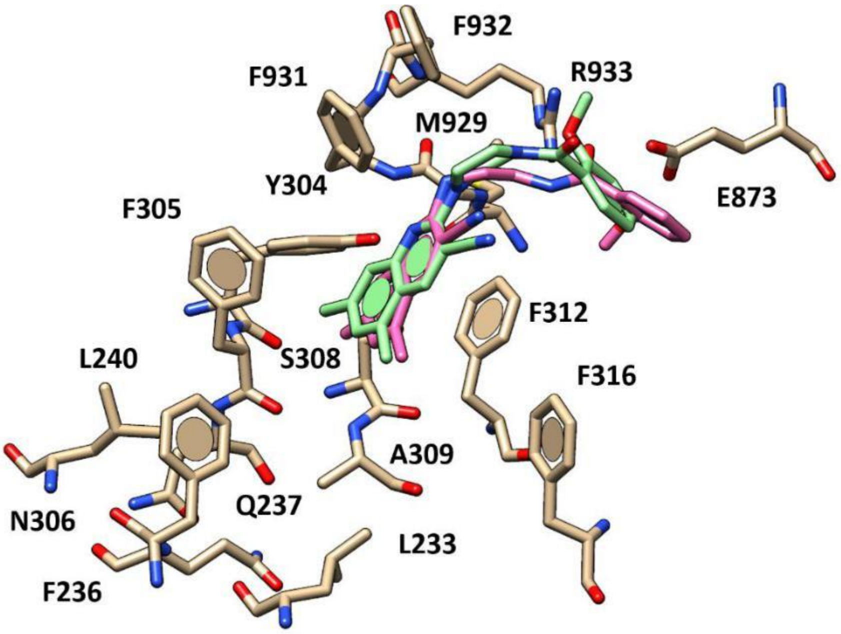

S21. Docking positioning of compound **31** (*C* atom; yellow) compared to that of compound **45** (*C* atom; light green) within the human CFTR protein.

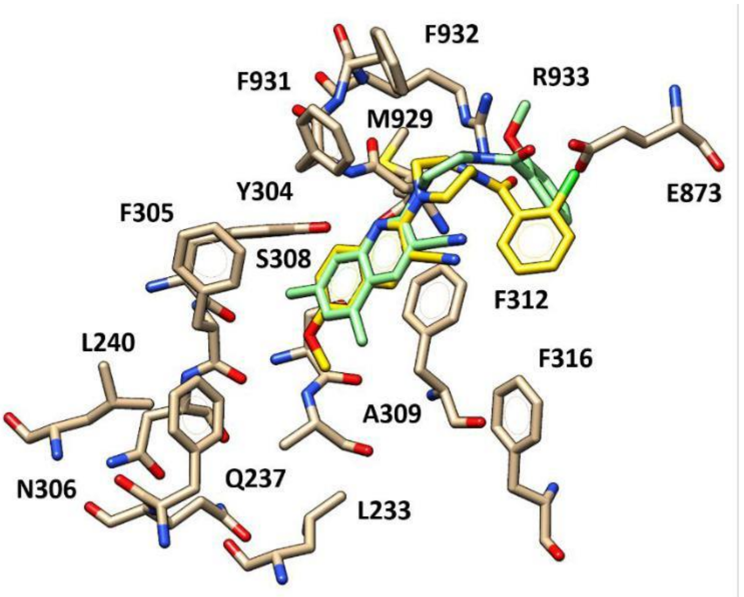

S22. Ligplot of the derived docking poses of compound **62** *(left)* and **67**, **64** *(right; upper and lower side)* at the CFTR cavity.

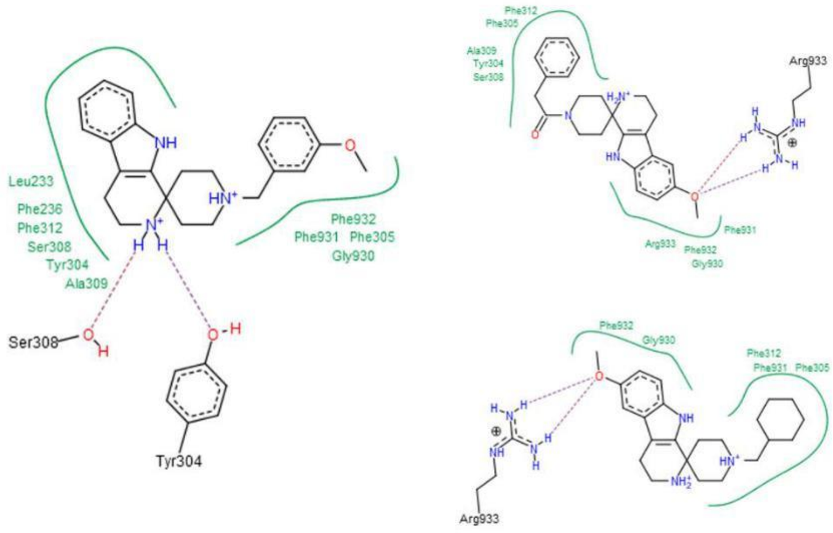

S23. Docking positioning of compound **80** (*C* atom; green) within the X-ray crystallographic structure of the human CFTR in presence of the docked VX-770 (*C* atom; magenta).

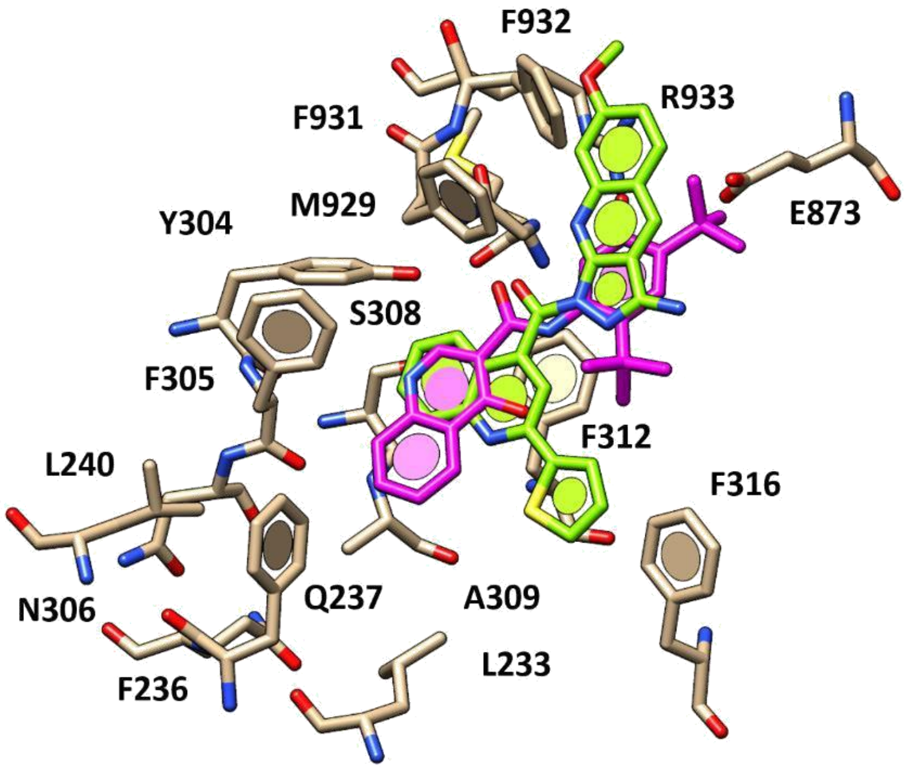

S24. Alignment of derivatives **1**-**26** used for the development of the pharmacophore model. Compound **11** has been used as reference ligand.

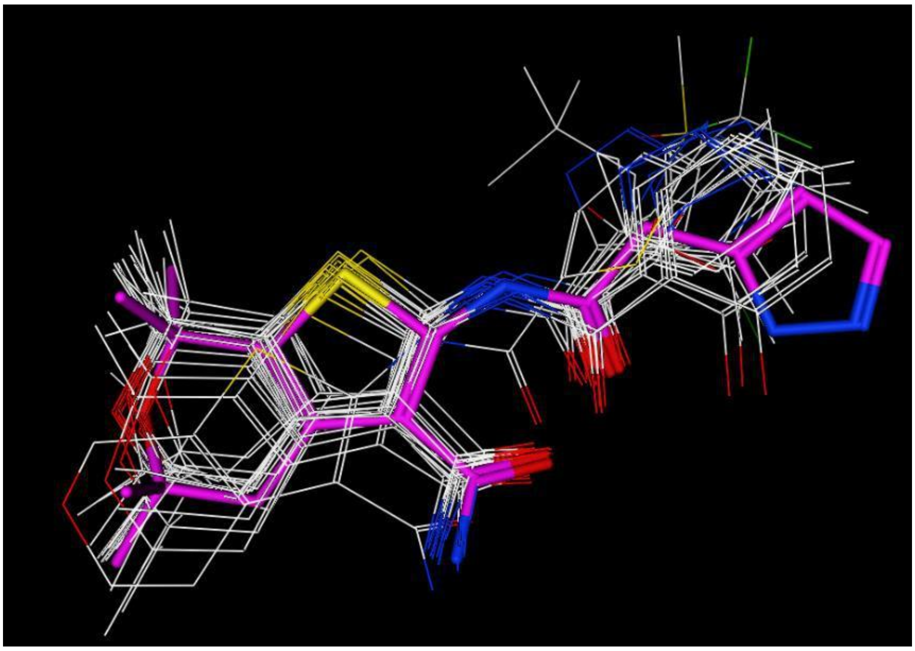

S25. Distanced among the pharmacophore features exhibited by at least the 80% of the thieno-containing derivatives **1**-**26**, acting as CFTR potentiators.

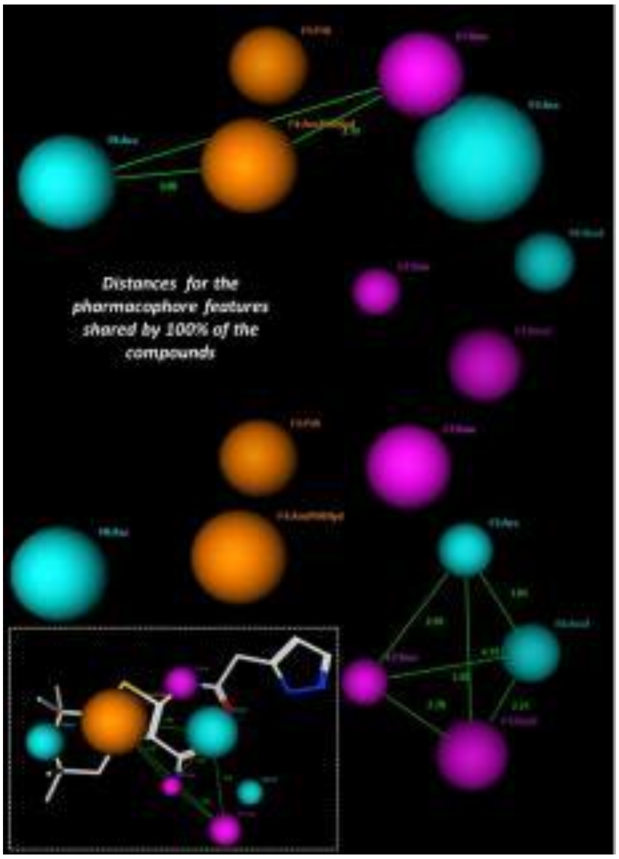

S26. Experimental (Exp.pEC_{50}) and predicted (Pred.pEC_{50}) values of the training set compounds according to the developed QSAR model. The selected 2D Adjacency and Distance Matrix Descriptors, Pharmacophore Feature Descriptors, Physical Properties, Atom Counts and Bond Counts descriptors and the 3D Conformation Dependent Charge Descriptors values have been reported.

Cp.	Exp. pEC_{50}	BCUT_SLOGP_1	BCUT_SMR_1	BCUT_SMR_2	BCUT_SMR_3	b_1rotR	logS	dipoleY	a_hyd	ASA+	Pred. pEC_{50}	Residual
**1**	6.18	-0.35568	-0.28266	0.798257	2.883142	0.130435	-4.78087	1.204581	15	371.5502	6.12	0.0584
**4**	7.88	-0.32796	-0.25849	0.652405	2.905984	0.12	-4.79947	1.044069	16	390.8534	7.32	0.5626
**7**	6.72	-0.35712	-0.26889	0.656855	2.955143	0.107143	-4.025	0.745493	16	382.3849	6.94	-0.2237
**8**	6.83	-0.34052	-0.25029	0.62132	2.955196	0.15625	-4.22493	1.129376	17	390.6831	6.59	0.2433
**9**	6.33	-0.34229	-0.26413	0.633213	2.955108	0.115385	-3.76415	0.740564	12	340.8268	6.95	-0.6186
**10**	6.90	-0.24732	-0.14753	0.604097	2.955141	0.115385	-3.76987	0.922183	15	354.6852	7.36	-0.4613
**12**	6.86	-0.25409	-0.13852	0.544934	2.955387	0.148148	-3.98426	0.652829	16	374.0897	7.08	-0.2195
**22**	8.69	-0.24278	-0.14413	0.618789	2.955143	0.111111	-4.65708	0.762706	16	386.3489	8.07	0.6208
**23**	8.69	-0.12432	-0.0424	0.496516	2.95516	0.111111	-4.08326	0.870343	16	369.6105	8.03	0.6621
**25**	7.88	-0.21336	-0.11747	0.566128	2.955142	0.111111	-4.23618	0.861456	16	389.3108	7.90	-0.0228
**26**	7.95	-0.19151	-0.06593	0.597892	2.95515	0.107143	-4.97047	0.794395	17	413.9033	8.50	-0.5481
**27**	4.82	-0.54106	-0.41293	0.753114	2.741984	0.225806	-4.63377	-0.6466	19	352.3749	4.31	0.5125
**28**	4.82	-0.57448	-0.41224	0.756553	2.741531	0.225806	-4.94722	-0.54837	19	373.7593	4.60	0.2203
**29**	4.85	-0.47238	-0.41315	0.724984	2.741931	0.185185	-5.12705	-0.83096	17	348.8117	5.18	-0.3335
**33**	5.22	-0.51784	-0.38127	0.74058	2.741941	0.2	-5.37076	0.008091	19	368.5545	5.48	-0.2638
**30**	4.95	-0.51287	-0.423	0.763975	2.924864	0.125	-4.8917	0.258115	20	329.1659	5.12	-0.1726

**31**	5.53	-0.47932	-0.38118	0.7931	2.925107	0.125	-5.33101	0.229203	20	374.0451	5.37	0.1581
**34**	5.63	-0.50703	-0.40016	0.750418	2.742956	0.2	-5.37076	-0.19278	19	365.1611	5.51	0.1183
**35**	5.38	-0.51276	-0.37429	0.73428	2.741745	0.2	-5.37076	-0.11834	19	368.825	5.22	0.1587
**36**	5.92	-0.47947	-0.40816	0.738283	2.739221	0.178571	-4.21516	-0.32604	17	333.2777	5.14	0.7827
**37**	4.87	-0.50561	-0.41076	0.752206	2.740608	0.178571	-4.06224	-0.05267	17	314.3405	5.14	-0.2747
**39**	5.00	-0.53808	-0.43228	0.76661	2.741382	0.178571	-5.32038	-0.11748	18	336.9756	5.18	-0.1837
**41**	4.31	-0.50842	-0.323	0.720619	2.742418	0.21875	-5.42114	0.140607	20	244.9699	4.62	-0.3131
**43**	5.46	-0.48756	-0.34619	0.715961	2.739452	0.2	-4.26554	-0.41474	18	364.4208	5.20	0.2614

**44**	4.93	-0.47628	-0.35879	0.734753	2.744759	0.21875	-5.42114	-0.54154	20	391.6472	5.35	-0.4220
**45**	6.00	-0.45021	-0.3566	0.742185	2.92537	0.121212	-5.54456	-0.63642	21	390.8438	5.77	0.2321
**46**	5.92	-0.47322	-0.34009	0.724696	2.925081	0.121212	-5.54456	-0.33508	21	390.7534	5.72	0.1971
**47**	5.88	-0.50543	-0.33309	0.725162	2.925036	0.121212	-5.54456	-0.50312	21	395.551	5.64	0.2367
**48**	5.13	-0.43985	-0.34697	0.726749	2.924191	0.096774	-4.38896	-0.57797	19	354.7831	5.55	-0.4168
**51**	4.55	-0.50608	-0.40037	0.747983	2.792774	0.225806	-5.57253	-0.28617	20	389.7123	4.93	-0.3806
**52**	5.33	-0.5166	-0.38144	0.737511	2.7923	0.225806	-5.57253	-0.04607	20	389.6762	5.41	-0.0813
**53**	5.30	-0.51268	-0.37444	0.730729	2.792214	0.225806	-5.57253	-0.13285	20	391.6064	5.33	-0.0334
**54**	5.21	-0.47884	-0.40832	0.735546	2.790994	0.206897	-4.41693	-0.20078	18	350.9011	5.04	0.1700
**56**	4.87	-0.53307	-0.42304	0.765963	2.791864	0.206897	-4.26401	0.021417	18	340.02	5.01	-0.1352
**58**	5.69	-0.50701	-0.412	0.750792	3.034299	0.090909	-4.61058	-0.22471	25	398.6733	5.47	0.2185
**60**	5.29	-0.39696	-0.27355	0.776773	3.034615	0.096774	-3.35656	-0.37939	22	429.9749	5.23	0.0564
**61**	4.79	-0.50193	-0.43781	0.787634	3.034265	0.068966	-3.97024	-0.10717	22	402.551	4.76	0.0311
**62**	4.88	-0.48485	-0.41943	0.745826	3.034298	0.096774	-4.02062	-0.1516	23	428.7566	4.77	0.1099
**63**	4.52	-0.44583	-0.36277	0.718034	3.047983	0.09375	-4.15157	-0.22446	24	429.1572	4.57	-0.0495
**64**	4.52	-0.49038	-0.37638	0.700285	3.061642	0.096774	-4.42709	-0.11681	23	488.7885	4.94	-0.4189
**65**	4.52	-0.53808	-0.4561	0.823608	3.028354	0.066667	-4.13893	-0.51908	21	367.1241	4.92	-0.4048
**67**	4.52	-0.52735	-0.43351	0.783545	3.03069	0.121212	-4.25078	-0.57581	23	414.729	4.48	0.0375
**68**	4.52	-0.44858	-0.27355	0.67972	3.058267	0.074074	-2.23887	0.122182	17	351.7489	4.75	-0.2291
**69**	4.52	-0.3969	-0.27356	0.67976	3.030895	0.041667	-2.25272	-0.17549	17	362.3517	4.19	0.3275
												0.2087

**71**	4.52	-0.53808	-0.49986	0.854311	2.582083	0.111111	-4.3668	-0.30719	14	300.6806	4.31	
**73**	4.52	-0.54843	-0.34375	0.705257	2.61022	0.181818	-5.77608	-0.46157	18	380.1791	4.79	-0.2698
**74**	5.61	-0.49763	-0.41475	0.867923	2.587962	0.103448	-6.65421	-0.27854	17	335.5798	5.51	0.0977
**75**	4.52	-0.5085	-0.3597	0.719454	2.606652	0.129032	-6.57564	-1.07103	17	298.6164	4.82	-0.3039
**76**	4.52	-0.53808	-0.45456	0.854311	2.643398	0.16129	-6.47664	-1.6727	17	322.3822	4.63	-0.1123
**78**	5.76	-0.5087	-0.40627	0.92341	2.591069	0.129032	-7.14946	-1.06612	17	314.7956	5.08	0.6800
**79**	4.52	-0.5071	-0.34196	0.717573	2.653562	0.15625	-6.1306	-0.849	15	341.0613	4.42	0.1031
**80**	6.52	-0.50761	-0.43667	0.885183	2.606634	0.105263	-8.23121	-0.67945	23	378.4928	6.34	0.1828
**81**	4.46	-0.52308	-0.46709	0.905181	2.609082	0.166667	-6.24408	-1.06445	17	304.207	4.86	-0.3981
**82**	4.56	-0.49315	-0.45805	0.909364	2.608691	0.173913	-6.18813	-0.7946	18	321.3681	4.13	0.4298
**83**	4.27	-0.49295	-0.45743	0.936491	2.609314	0.173913	-6.62744	-0.54232	18	305.2247	4.84	-0.5710

**85**	4.14	-0.49312	-0.45774	0.909421	2.633948	0.153846	-5.84825	-0.54119	17	371.2936	4.32	-0.1769
**86**	5.00	-0.47654	-0.40829	0.92898	2.606236	0.190476	-5.02206	-0.49993	15	329.1525	4.29	0.7073
**87**	4.23	-0.34205	-0.28855	0.920589	2.609608	0.230769	-5.37016	-0.6221	15	400.5386	4.59	-0.3630
**89**	5.71	-0.51372	-0.37607	0.913362	2.750752	0.148148	-6.27911	-0.7058	18	377.1368	377.1368	0.3582
**90**	5.20	-0.4931	-0.45771	0.93233	2.697315	0.148148	-6.72573	-0.83967	18	382.8594	382.8594	-0.3414

S27. Experimental (Exp.pEC$_{50}$) and predicted (Pred.pEC$_{50}$) values of the training set compounds according to the developed QSAR model. The selected 2D

Subdivided Surface Area, Adjacency and Partial Charge descriptors and the 3D Surface Area, Volume and Shape Descriptor values have been reported.

**Cp.**	**Exp. pEC$_{50}$**	**PEOE _VSA+5**	**PEOE_ VSA-6**	**SlogP _VSA4**	**SlogP _VSA5**	**SlogP _VSA9**	**SMR _VSA2**	**SMR _VSA4**	**vsurf_ ID1**	**Vsurf _ID7**	**vsurf_Wp2**	**vsurf_Wp3**	**Pred. pEC$_{50}$**	**Residual**
**1**	6.18	12.94953	6.788011	58.17079	37.73681	96.81097	0	51.83751	0.800828	0.493301	469.375	148.875	6.12	0.0584
**4**	7.88	12.94953	9.291766	58.17079	18.86841	184.3893	0	51.83751	1.128163	1.685388	472.75	159.25	7.32	0.5626
**7**	6.72	25.89906	17.05931	58.63956	18.86841	170.3259	0	51.83751	0.600447	1.687995	513.75	173.375	6.94	-0.2237
**8**	6.83	25.89906	14.97434	58.63956	50.55958	167.8393	0	70.57043	0.27258	0.752095	542.375	181.5	6.59	0.2433
**9**	6.33	25.89906	9.428658	57.7819	18.86841	165.5113	16.78553	51.83751	0.430511	1.366267	529.875	206.125	6.95	-0.6186
**10**	6.90	25.89906	9.291766	58.63956	18.86841	165.5113	16.66301	51.83751	0.565331	1.356548	523.75	197.875	7.36	-0.4613
**12**	6.86	12.94953	9.291766	34.34852	37.73681	165.5113	16.66301	51.83751	0.817541	1.646662	503.125	184.875	7.08	-0.2195
**22**	8.69	25.89906	9.291766	58.21073	18.86841	204.66	16.66301	51.83751	1.014075	1.970239	514.375	191.5	8.07	0.6208
**23**	8.69	25.89906	9.291766	61.39631	18.86841	198.8373	16.66301	51.83751	0.951192	1.522542	509.75	188.625	8.03	0.6621
**25**	7.88	25.89906	9.291766	60.96748	18.86841	198.8373	16.66301	51.83751	0.888762	1.297468	516.125	204.875	7.90	-0.0228
**26**	7.95	25.89906	9.291766	60.96748	18.86841	237.986	16.66301	51.83751	1.027718	1.585446	515.75	195.5	8.50	-0.5481

**27**	4.82	12.94953	5.144404	33.41894	70.76774	46.14079	18.01075	25.05577	0.653004	1.248182	516	123.125	4.31	0.5125
**28**	4.82	12.94953	5.144404	33.41894	70.76774	46.14079	18.01075	25.05577	0.731779	1.509997	521.875	128.875	4.60	0.2203
**29**	4.85	0	0.136891	34.77734	0	100.2576	18.01075	25.05577	0.610643	0.692451	504.125	136.875	5.18	-0.3335
**33**	5.22	12.94953	2.640647	36.60452	35.38387	74.22338	18.01075	25.05577	0.947415	1.384679	512.875	136.375	5.48	-0.2638
**30**	4.95	12.94953	2.503756	30.23337	35.38387	22.53146	12.24533	7.045022	1.173449	1.768574	473.5	80.375	5.12	-0.1726
**31**	5.53	12.94953	2.503756	30.23337	35.38387	46.71999	3.124314	7.045022	0.719132	1.033341	493.5	84.25	5.37	0.1581
**34**	5.63	12.94953	2.640647	36.60452	35.38387	74.22338	18.01075	25.05577	1.163676	1.649628	508.125	132.75	5.51	0.1183
**35**	5.38	12.94953	2.640647	36.60452	35.38387	74.22338	18.01075	25.05577	0.967555	0.913348	509.375	131.25	5.22	0.1587

**36**	5.92	12.94953	0.136891	36.17569	0	68.97995	18.01075	25.05577	0.899112	1.16417	507.5	132.75	5.14	0.7827
**37**	4.87	12.94953	0.136891	36.60452	0	68.97995	18.01075	25.05577	1.274256	1.278531	498.5	132.125	5.14	-0.2747
**39**	5.00	12.94953	0.136891	36.60452	0	68.97995	18.01075	25.05577	0.966741	0.86576	489.125	128.25	5.18	-0.1837
**41**	4.31	12.94953	5.144404	36.60452	70.76774	79.46681	18.01075	25.05577	1.189321	1.069763	440.75	118.125	4.62	-0.3131
**43**	5.46	12.94953	2.640647	36.17569	35.38387	74.22338	18.01075	25.05577	1.041974	1.364302	526.125	138.25	5.20	0.2614
**44**	4.93	12.94953	5.144404	36.60452	70.76774	79.46681	18.01075	25.05577	1.193353	1.539765	516.75	135.875	5.35	-0.4220
**45**	6.00	12.94953	2.503756	36.60452	35.38387	74.22338	3.124314	7.045022	0.906389	1.459584	485.25	93	5.77	0.2321
**46**	5.92	12.94953	2.503756	36.60452	35.38387	74.22338	3.124314	7.045022	0.900818	1.2158	486.375	92.625	5.72	0.1971
**47**	5.88	12.94953	2.503756	36.60452	35.38387	74.22338	3.124314	7.045022	0.933468	1.22687	495	89.875	5.64	0.2367
**48**	5.13	12.94953	0	36.17569	0	68.97995	3.124314	7.045022	0.752685	1.216956	484.75	92.375	5.55	-0.4168
**51**	4.55	12.94953	2.640647	36.60452	35.38387	74.22338	18.01075	25.05577	0.160016	0.717382	512.625	110.875	4.93	-0.3806
**52**	5.33	12.94953	2.640647	36.60452	35.38387	74.22338	18.01075	25.05577	0.558261	1.108011	522.75	128.75	5.41	-0.0813
**53**	5.30	12.94953	2.640647	36.60452	35.38387	74.22338	18.01075	25.05577	0.470551	0.787824	540.625	131.875	5.33	-0.0334
**54**	5.21	12.94953	0.136891	36.17569	0	68.97995	18.01075	25.05577	0.326577	0.776319	522.375	125.25	5.04	0.1700
**56**	4.87	12.94953	0.136891	36.60452	0	68.97995	18.01075	25.05577	0.701703	0.832017	529.125	126	5.01	-0.1352
**58**	5.69	0	2.777538	9.127897	54.25227	53.60323	53.03832	4.717102	1.188067	2.009089	510	122.5	5.47	0.2185
**60**	5.29	0	2.777538	10.05747	54.25227	88.28671	34.79628	4.717102	0.357714	0.54331	526	130	5.23	0.0564
**61**	4.79	0	0.273782	9.127897	18.86841	18.43958	34.79628	4.717102	0.498902	0.477126	490.375	113.875	4.76	0.0311
**62**	4.88	0	2.777538	9.127897	54.25227	23.68301	34.79628	4.717102	0.439908	0.629279	516.125	121.125	4.77	0.1099
**63**	4.52	0	2.640647	9.127897	87.14946	23.68301	16.78553	4.717102	0.393936	0.685366	484.125	86.75	4.57	-0.0495
														-0.4189

**64**	4.52	0	2.777538	10.35312	54.25227	5.243428	39.20708	4.717102	0.212023	0.722736	482.75	118	4.94	
**65**	4.52	12.94953	0.273782	32.99011	18.86841	0	34.79628	4.717102	0.848914	1.119773	480.375	119.375	4.92	-0.4048
**67**	4.52	0	2.777538	9.127897	73.12068	5.243428	34.79628	4.717102	0.449963	0.695605	527.5	128.375	4.48	0.0375
**68**	4.52	0	2.777538	5.942323	85.94345	5.243428	34.79628	4.717102	0.794642	2.366449	434.75	121	4.75	-0.2291
**69**	4.52	0	2.777538	5.942323	87.14946	5.243428	34.79628	4.717102	0.537017	1.531974	395.375	106.625	4.19	0.3275
**71**	4.52	12.94953	2.503756	27.04779	35.38387	7.571348	0	11.3333	0.701	0.923499	495.125	146.5	4.31	0.2087
**73**	4.52	12.94953	10.01502	27.04779	141.5355	23.30163	0	11.3333	0.73529	1.624358	534.25	161.25	4.79	-0.2698
**74**	5.61	12.94953	2.503756	27.04779	35.38387	61.68011	9.121018	11.3333	0.84081	1.107921	491.5	144.75	5.51	0.0977
**75**	4.52	19.64908	2.503756	30.23337	35.38387	43.65411	50.93501	14.09004	0.715313	0.807208	532.25	153.25	4.82	-0.3039
**76**	4.52	19.64908	2.503756	27.04779	54.25227	10.3281	50.93501	14.09004	0.61446	1.191052	581.875	166.875	4.63	-0.1123
**78**	5.76	19.64908	2.503756	27.04779	35.38387	49.47674	50.93501	14.09004	0.640504	0.81883	540.875	165.125	5.08	0.6800

**79**	4.52	12.94953	36.4348	26.61896	35.38387	100.8135	50.93501	13.66122	0.505315	0.708749	4.42	0.1031	4.42	0.1031
**80**	6.52	12.94953	2.503756	27.04779	35.38387	38.84904	0	20.16555	0.511739	0.588034	6.34	0.1828	6.34	0.1828
**81**	4.46	0	0	3.185575	33.24191	66.75472	1.550734	27.21057	0.739348	2.128536	4.86	-0.3981	4.86	-0.3981
**82**	4.56	0	0	0	33.24191	81.71484	10.67175	27.21057	0.286104	0.822712	4.13	0.4298	4.13	0.4298
**83**	4.27	0	0	0	33.24191	105.9034	1.550734	27.21057	0.587894	1.361566	4.84	-0.5710	4.84	-0.5710
**85**	4.14	0	5.007512	0	33.24191	77.24158	1.550734	27.21057	0.294075	0.617597	4.32	-0.1769	4.32	-0.1769
**86**	5.00	0	0	0	33.24191	98.03241	1.550734	24.95457	0.525247	1.002255	4.29	0.7073	4.29	0.7073
**87**	4.23	0	8.406116	26.61896	42.97603	100.0807	1.550734	29.47381	0.452959	0.437706	4.59	-0.3630	4.59	-0.3630
**89**	5.71	0	0.136891	3.185575	52.11031	69.51147	1.550734	47.97807	0.436705	0.886085	377.1368	0.3582	377.1368	0.3582
**90**	5.20	14.92559	0.136891	0	34.79264	125.5932	3.101468	47.97807	0.730122	0.597511	382.8594	-0.3414	382.8594	-0.3414

S28. Experimental (Exp.pEC$_{50}$) and predicted (Pred.pEC$_{50}$) values of the test set compounds according to the developed QSAR model. The selected 2D Adjacency and Distance Matrix Descriptors, Pharmacophore Feature Descriptors, Physical Properties, Atom Counts and Bond Counts descriptors and the 3D Conformation Dependent Charge Descriptors values have been reported.

**Cp.**	**Exp. pEC$_{50}$**	**BCUT_SLOGP_1**	**BCUT_SMR_1**	**BCUT_SMR_2**	**BCUT_SMR_3**	**b_1rotR**	**logS**	**dipoleY**	**a_hyd**	**ASA+**	**Pred. pEC$_{50}$**	**Residual**
**3**	6.10	-0.33047944	-0.26404753	0.73366147	2.8013721	0.13043478	-4.14505	1.025835	14	349.56198	6.47	-0.3730
**6**	8.39	-0.17167728	-0.11908972	0.59196395	2.9551604	0.11111111	-5.4538898	1.0793978	18	416.81415	8.50	-0.1134
**11**	8.52	-0.2496783	-0.14757159	0.60403872	2.9551349	0.11538462	-3.9227901	1.0493044	15	352.38104	7.60	0.9194
**24**	8.52	-0.21303622	-0.1171928	0.5663017	2.9551418	0.1	-5.1322598	0.47866929	19	354.41968	7.96	0.5565

**32**	5.30	-0.47616071	-0.36629966	0.80957109	2.9251747	0.125	-5.6871099	0.31962168	20	390.99222	5.67	-0.3738
**38**	5.00	-0.53411287	-0.42294818	0.76985401	2.7408793	0.17857143	-4.0622401	0.090998821	17	314.42032	4.92	0.0811
**40**	4.25	-0.50695312	-0.33814892	0.72322482	2.7434282	0.21875	-5.4211402	-0.35460791	20	369.87402	5.03	-0.7757
**55**	4.79	-0.5048244	-0.41092402	0.74816161	2.7917297	0.20689656	-4.26401	0.043317597	18	341.32532	5.08	-0.2927
**57**	5.00	-0.50591195	-0.34110171	0.69058079	3.0343418	0.12121212	-4.0710001	-0.099414699	24	451.85364	4.62	0.3786
**59**	5.58	-0.50857282	-0.41164595	0.75098193	3.034306	0.090909094	-4.61058	-0.23075099	25	399.97519	5.23	0.3450
**66**	4.52	-0.50590354	-0.33911341	0.68981081	3.0312951	0.11111111	-2.6305399	-0.617037	18	383.77551	4.33	0.1878

**72**	5.44	-0.52836478	-0.46312457	0.81207639	2.5848756	0.10714286	-5.91992	-0.3858822	16	307.17242	4.96	0.4835
**77**	4.52	-0.50872862	-0.36945948	0.8581714	2.5899618	0.12903225	-7.1494598	-0.35955641	17	324.83478	5.28	-0.7613
**84**	5.19	-0.49309871	-0.45771432	0.93088794	2.6487799	0.14814815	-6.67063	-0.80477411	17	343.14407	5.12	0.0659
**88**	4.49	-0.34952211	-0.29147619	0.93386561	2.6100163	0.18181819	-6.24999	-0.531663	17	384.87665	5.60	-1.1057

S29. Experimental (Exp.pEC_{50}) and predicted (Pred.pEC_{50}) values of the test set compounds according to the developed QSAR model. The selected 2D Subdivided Surface Area, Adjacency and Partial Charge descriptors and the 3D Surface Area, Volume and Shape Descriptor values have been reported.

**Cp.**	**Exp. pEC_{50}**	**PEOE _VSA+5**	**PEOE_ VSA-6**	**SlogP _VSA4**	**SlogP _VSA5**	**SlogP _VSA9**	**SMR _VSA2**	**SMR _VSA4**	**vsurf_ ID1**	**Vsurf _ID7**	**vsurf_Wp2**	**vsurf_Wp3**	**Pred. pEC_{50}**	**Residual**
**3**	6.10	12.949531	9.2917662	58.170788	18.868406	117.73722	0	51.837513	1.4823005	1.6377909	459.875	149.625	6.47	-0.3730
**6**	8.39	12.949531	9.2917662	58.170788	18.868406	230.11502	0	51.837513	1.0830811	1.896114	480.875	162.625	8.50	-0.1134
**11**	8.52	25.899061	9.2917662	58.210732	18.868406	165.51132	16.663008	51.837513	0.84125829	1.6844577	517.75	199.375	7.60	0.9194
**24**	8.52	25.899061	9.2917662	60.96748	18.868406	210.39166	24.422523	51.837513	1.641968	0.92884934	507.25	209	7.96	0.5565
**32**	5.30	12.949531	2.503756	30.233366	38.438972	53.52544	6.1794186	7.0450215	0.92542022	1.2124147	506.125	83.875	5.67	-0.3738
**38**	5.00	12.949531	0.13689101	36.604515	0	68.97995	18.01075	25.055773	1.0961233	0.84826136	502.25	132.5	4.92	0.0811
**40**	4.25	12.949531	5.1444035	36.604515	70.767738	79.466805	18.01075	25.055773	1.2389504	1.0051649	500.375	131.625	5.03	-0.7757
**55**	4.79	12.949531	0.13689101	36.604515	0	68.97995	18.01075	25.055773	0.69999182	0.85419071	525.75	125.625	5.08	-0.2927
**57**	5.00	0	5.2812943	9.1278973	89.636139	28.926434	34.79628	4.7171016	0.26172695	0.41990376	527.75	127.625	4.62	0.3786
		0	2.7775381	9.1278973	54.252274	53.603233	53.038315	4.7171016	0.90120083	1.5566729	508.625	122	5.23	0.3450

**59**	5.58													
**66**	4.52	0	2.7775381	5.9423227	54.252274	38.569443	34.79628	4.7171016	0.70693582	1.4608345	430	115.125	4.33	0.1878
**72**	5.44	12.949531	2.503756	27.047791	35.383869	22.531462	9.1210184	11.333296	0.81351507	1.249822	484	147.25	4.96	0.4835
**77**	4.52	19.649082	2.503756	27.047791	35.383869	49.476738	50.935009	14.090043	0.6767658	0.71812558	540.875	163	5.28	-0.7613
**84**	5.19	0	0	0	33.241909	98.617111	1.5507339	47.978065	0.68520218	0.61810213	524.25	138.625	5.12	0.0659
**88**	4.49	0	0	0	33.241909	137.18106	1.5507339	25.383394	0.48546532	1.3971066	448.5	96.375	5.60	-1.1057

S30. Distribution of the b_1RotR (*up*) and a_hyd (*down*) descriptor values with respect to the experimental (Exp.pEC50) potency data of the dataset compounds (shown as white dots).

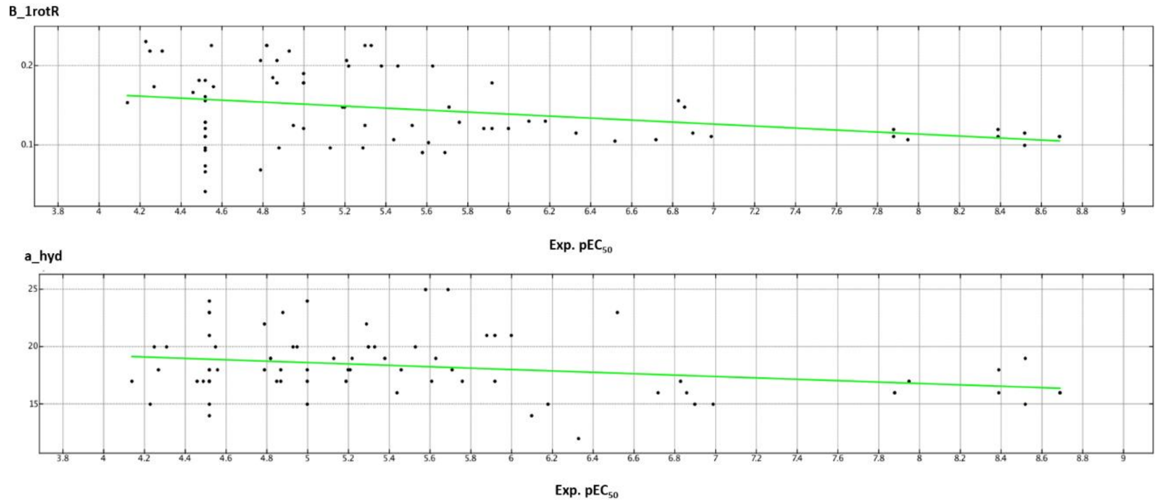

S31. Distribution of the ASA^{+} (*up*), LogS and BCUT_SMR3 (*down*) descriptor values with respect to the experimental (Exp.pEC50) potency data of the dataset compounds (shown as white dots).

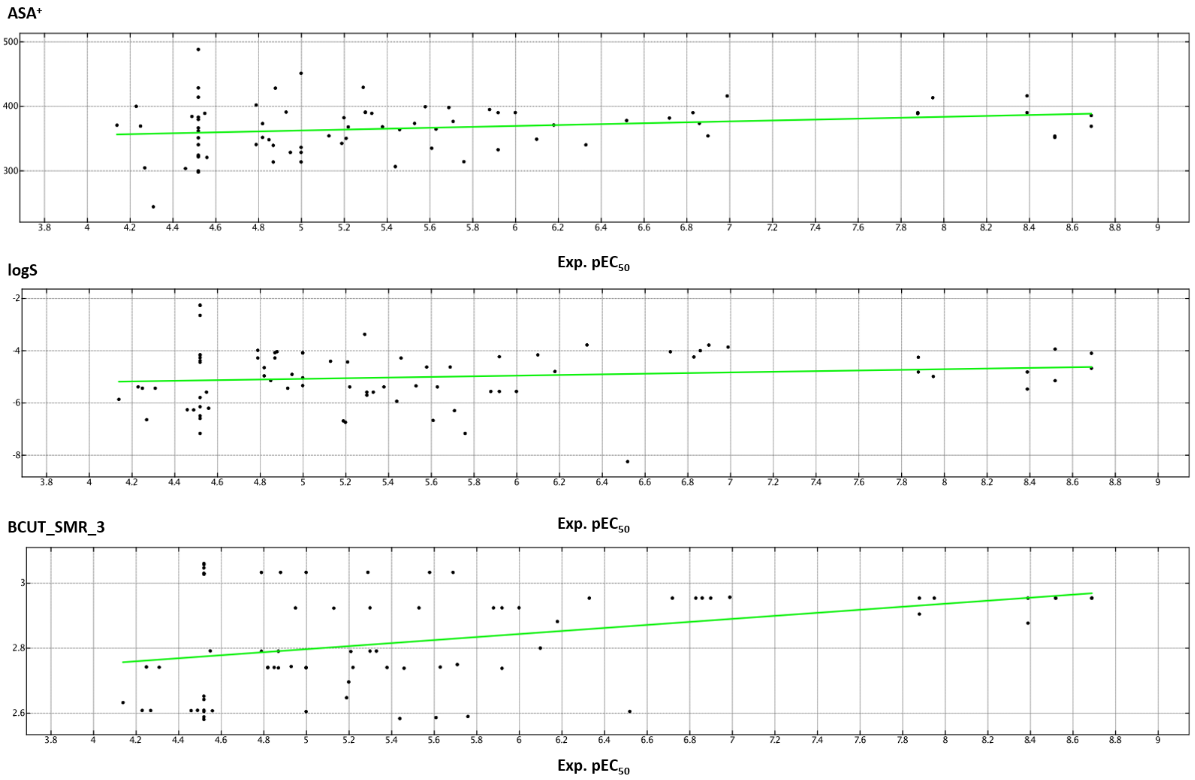

S32. Pattern of the most important interactions within the CFTR protein observed for the most promising GLPG1837 analogues (pEC_{50} = 7.88-9.26).

**Potentiator**	**H-bonds**		**π-π stacking**		**Cation - π**		**Van der Waals**	
	Amino acid residues	Ligand portion	Amino acid residues	Ligand portion	Amino acid residues	Ligand portion	Amino acid residues	Ligand portion
**4**	F931	oxygen atom of carbonyl moiety	F312	Thienopyran-based core	R933	Thienopyran-based core	A309	Thienopyran-based core
	Y304	Primary amide group						
	F932	Hydroxyl group of phenol ring						
**5**	F931	Two oxygen atoms of carbonyl moieties	F305<br>F312	Thienopyran-based core	R933	Thienopyran-based core	A309	Thienopyran-based core
	Y304	Primary amide group						
	F932	Hydroxyl group of phenol ring						
**6**	F931	Two oxygen atoms of carbonyl moieties	F305<br>F312	Thienopyran-based core	R933	Thienopyran-based core	A309	Thienopyran-based core
	Y304	Primary amide group						
	F932	Oxygen atom of amide group						
**11**	F931	Two oxygen atoms of carbonyl moieties	F931<br>F932	Pyrazole ring			F236<br>F305<br>F312	Thienopyran-based core
	Y304	Primary amide group	F236<br>F305<br>F312	Thienopyran-based core				
	R933	Pyrazole ring						
**20**	F931	Two oxygen atoms of carbonyl moieties	F931<br>F932	Pyrazole ring			F236<br>F305<br>F312	Thienopyran-based core

	Y304	Primary amide group	F236 F305 F312	Thienopyran-based core				
	R933	Hydroxyl group						
**21 (*S* enantiomer)**	F932 Y304 S308	Two oxygen atoms of carbonyl moieties	F236 F305 F312	Thienopyran-based core			F236 F305 F312	Thienopyran-based core
	R933	Alkoxy group						
**22**	F931	Two oxygen atoms of carbonyl moieties	F932 F931	4-Cl-1H-pyrazole				
	Y304	Primary amide group	F236 F305 F312	Thienopyran-based core				
	R933	Alkoxy group						
**23**	F931	Two oxygen atoms of carbonyl moieties	F932 F931	3-methyl-1H-Pyrazole			F236 F305 F312	Thienopyran-based core
	Y304	Primary amide group	F236 F305	Thienopyran-based core				
	R933	Alkoxy group						

S33. Pattern of the most important interactions within the CFTR protein observed for the most promising CQs (pEC_{50} = 4.55-5.92).

**Potentiator**	**H-bonds**		**π-π stacking**		**Cation - π**		**Van der Waals**	
	Amino acid residues	Ligand portion	Amino acid residues	Ligand portion	Amino acid residues	Ligand portion	Amino acid residues	Ligand portion
**30**	F931	Methoxy group	F236	2-F-phenyl ring			F236 L233	Piperazine ring
	R933	Benzoyl moiety	F305 F312 Y304	Cyanoquinoline core				
**31**	F931	Methoxy group	F236	2-Cl-phenyl ring			F236 L233	Piperazine ring
	R933	Benzoyl moiety	F305 F312 Y404	Cyanoquinoline core				
**32**	F931	Methoxy group	F236	2-Br-phenyl ring			F236 L233	Piperazine ring
	R933	Benzoyl moiety	F305 F312 Y304	Cyanoquinoline core				

**34**	F931	Nitrogen atom of quinoline core	F236	o-Methoxy-benzoyl moiety				
	R933	Benzoyl moiety	F312 F931	Cyanoquinoline core				
**35**	F931	Nitrogen atom of quinoline core	F236	p-Methoxy-benzoyl moiety				
	R933	Benzoyl moiety	F312 F931	Cyanoquinoline core				
**45**	F931	Nitrogen atom of quinoline core	F931 F312	Cyanoquinoline core	R933	Methoxy-benzoyl moiety	F236 L233	Piperazine ring
	R933	Benzoyl group	F236	o-Methoxy-Benzoyl moiety				
**46**	F931	Nitrogen atom of quinoline core	F236	m-Methoxy-Benzoyl moiety	R933	Methoxy-benzoyl moiety	F236 L233	Piperazine ring
	R933	Benzoyl group	F312 F931	Cyanoquinoline core				
**47**	F931	Nitrogen atom of quinoline core	F236	p-Methoxy-Benzoyl ring	R933	Methoxy-benzoyl moiety	F236 L233	Piperazine ring
	R933	Benzoyl group	F312 F931	Cyanoquinoline core				
**53**	F931	Nitrogen atom of quinoline core	Y304 F236 F305	Cyanoquinoline core				
	R933	Benzoyl group						

S34. Pattern of the most important interactions within the CFTR protein observed for the most promising tetrahydropyridoindole derivatives (pEC_{50} = 5.00-5.58).

**Potentiator**	**H-bonds**		**π-π stacking**		**Cation - π**		**Van der Waals**	
	Amino acid residues	Ligand portion	Amino acid residues	Ligand portion	Amino acid residues	Ligand portion	Amino acid residues	Ligand portion
**57**	R933	Methoxy group	F236<br>Y304<br>F305	m-methoxy benzyl moiety	F931	Protonated nitrogen atom of tricyclic system	A309	m-methoxy benzyl moiety
**58**	R933	Methoxy group	F236<br>F305<br>Y304	2,4-di-F benzyl moiety	F931	Protonated nitrogen atoms of tricyclic system	A309	2,4-di-F benzyl moiety
**59**	R933	Methoxy group	F236<br>F305<br>Y304	3,4-di-F benzyl moiety	F931	Protonated nitrogen atoms of tricyclic system	A309	3,4-di-F benzyl moiety
**64**	R933	Methoxy group	F932<br>F305	Tricyclic system				

S35. Pattern of the most important interactions within the CFTR protein observed for the most promising pyrazoloquinolines analogues (pEC_{50} = 5.44-6.52).

Potentiator	H-bonds		π-π stacking		Cation - π		Van der Waals	
	Amino acid residues	Ligand portion	Amino acid residues	Ligand portion	Amino acid residues	Ligand portion	Amino acid residues	Ligand portion
**74**	F931	Pyrazole ring	F236 Y304 F305	2-Cl-4-F-benzoyl moiety			F312 F932	2-Cl-4-F-benzyl moiety
	M929 S308	Primary amine group						
	R933	Methoxy group						
**78**	F931	Nitrogen of tricyclic core	F236 Y304 F305	2-Cl-4-nitro-phenyl group				
	M929 S308	Primary amine group						
	R933	Nitro-group						
**80**	R933	Aromatic quinoline substituent	Y304 F305 F312 F316	Aromatic quinoline substituent	R933	Methoxy -group		

S36. Pattern of the most important interactions within the CFTR protein observed for the most promising AATs (pEC_{50} = 4.56-5.71).

Potentiator	H-bonds		π-π stacking		Cation - π		Van der Waals	
	Amino acid residues	Ligand portion	Amino acid residues	Ligand portion	Amino acid residues	Ligand portion	Amino acid residues	Ligand portion
**82**	F931	Thiazole nitrogen atom	F932	4-F-phenyl group			F305 F312	Aminoaryl-moiety
	Y304	Amine group	F312 F305	Aminoaryl-moiety				
**84**	R933	Carbamate moiety	F305	Benzoxalone ring				
	F931	Thiazole nitrogen atom	F312	Thiazole ring				
**86**	F931	Thiazole nitrogen atom	F932	Thiazole ring				
	Y304	Amine group						

S37. Comparison of the CQ **45** (*left*; *C* atom in green) and **50** (*right*; *C* atom in yellow) docking positioning with the pharmacophore model built onto the thienopyranes. Potentiator **11** is depicted as reference compound (*C* atom; white).

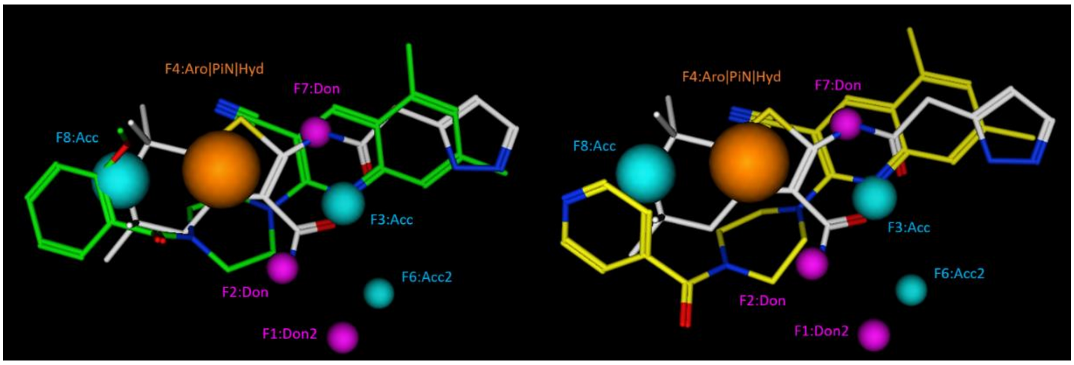

S38. Comparison of the pyrazolquinolines **80** (*left*; *C* atom in orange) and **71** (*right*; *C* atom in yellow) docking positioning with the pharmacophore model built onto the thienopyranes. Potentiator **11** is depicted as reference compound (*C* atom; white).

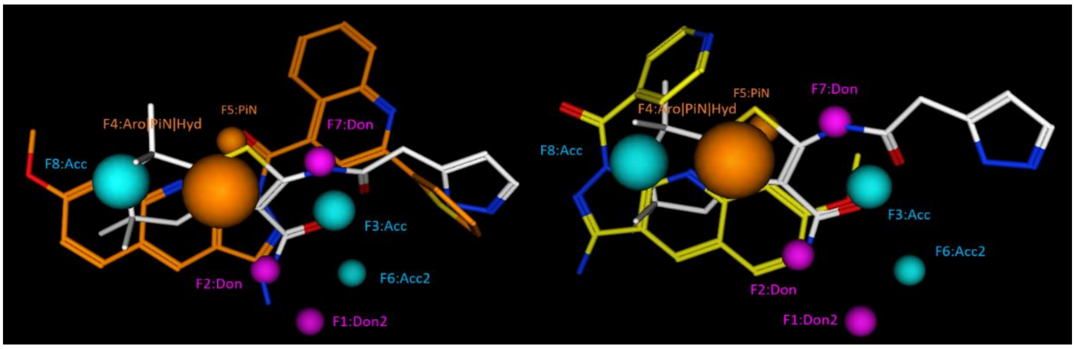

S39. Comparison of the AAT **89** (*left*; *C* atom in green) and **82** (*right*; *C* atom in green) docking positioning with the pharmacophore model built onto the thienopyranes. Potentiator **11** is depicted as reference compound (*C* atom; white).

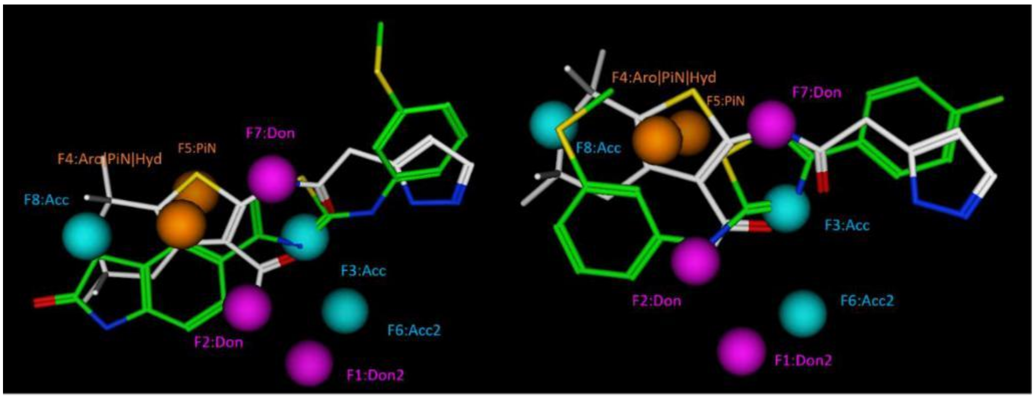

S40. Chemical structures of thiazole derivatives **91-106** as F508del-CFTR potentiators [40].

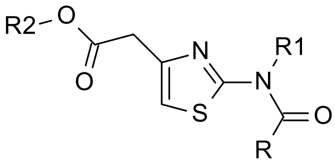

**Compound**	**R**	**R_1**	**R_2**
**91**	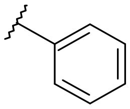	H	CH_2CH_3
**92**	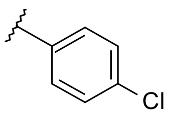	H	CH_2CH_3
**93**	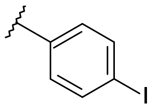	H	CH_2CH_3
**94**	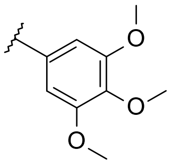	H	CH_2CH_3
**95**	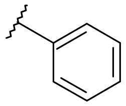	H	H
**96**	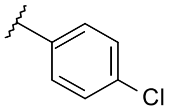	H	H
**97**	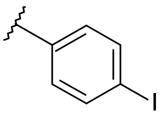	H	H

**98**	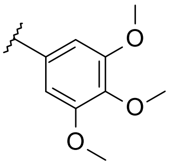	H	H
**99**	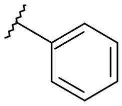	CH_2CH_3	CH_2CH_3
**100**	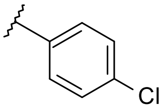	CH_2CH_3	CH_2CH_3
**101**	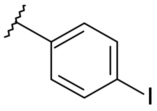	CH_2CH_3	CH_2CH_3
**102**	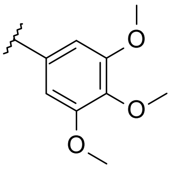	CH_2CH_3	CH_2CH_3
**103**	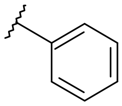	CH_2CH_3	H
**104**	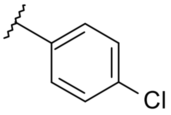	CH_2CH_3	H
**105**	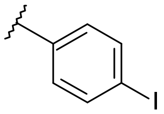	CH_2CH_3	H
**106**	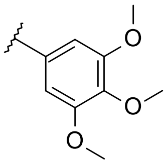	CH_2CH_3	H

S41. Chemical structures of aminoarylthiazoles **107-111** [22-23].

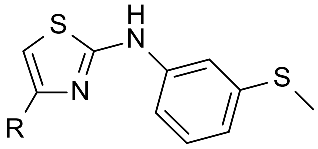

Compound	R
**107**	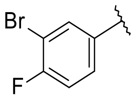
**108**	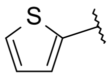
**109**	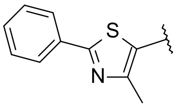
**110**	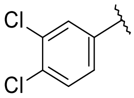
**111**	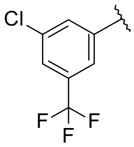

S42. Chemical structures of aminoarylthiazoles derivatives **112-139** [25].

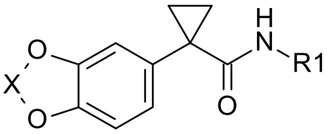

Compound	X	R_1
**112**	$-CH_2$	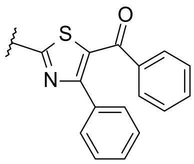
**113**	$-CH_2$	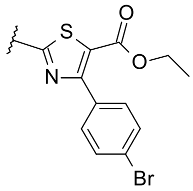

**114**	$-CH_2$	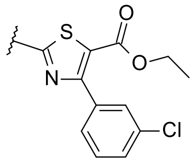
**115**	$-CH_2$	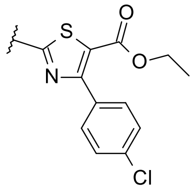
**116**	$-CH_2$	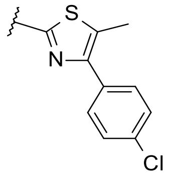
**117**	$-CH_2$	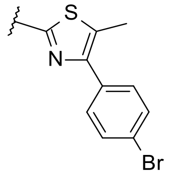
**118**	$-CH_2$	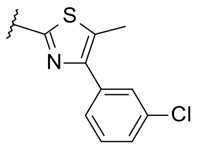
**119**	$-CH_2$	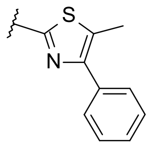
**120**	$-CH_2$	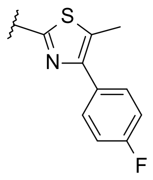
**121**	$-CH_2$	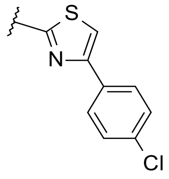
**122**	$-CH_2$	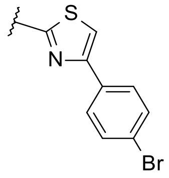
**123**	$-CH_2$	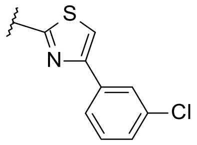

**124**	$-CH_2$	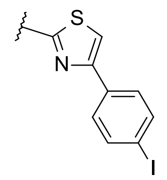
**125**	$-CH_2$	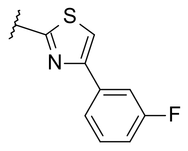
**126**	$-CH_2$	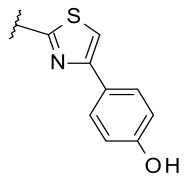
**127**	$-CH_2$	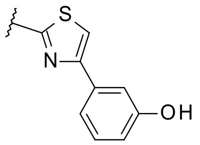
**128**	$-CH_2$	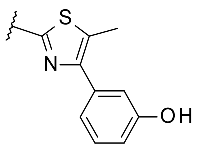
**129**	$-CH_2$	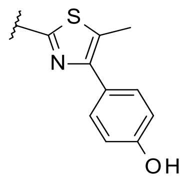
**130**	$-CH_2$	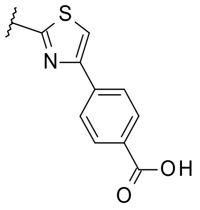
**131**	$-CH_2$	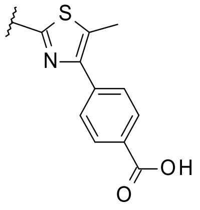
**132**	$-CH_2$	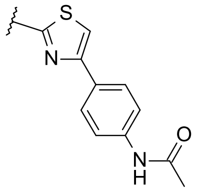

**133**	-CF_2	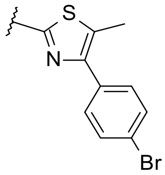
**134**	-CF_2	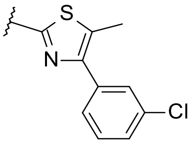
**135**	-CF_2	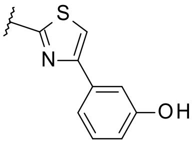
**136**	-CF_2	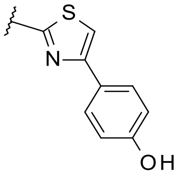
**137**	-CF_2	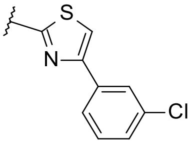
**138**	-CH_2	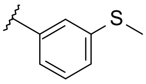
**139**	-CH_2	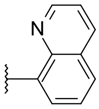

S43. Ligplot of the calculated docking mode of **100** and **101** within the CFTR cavity.

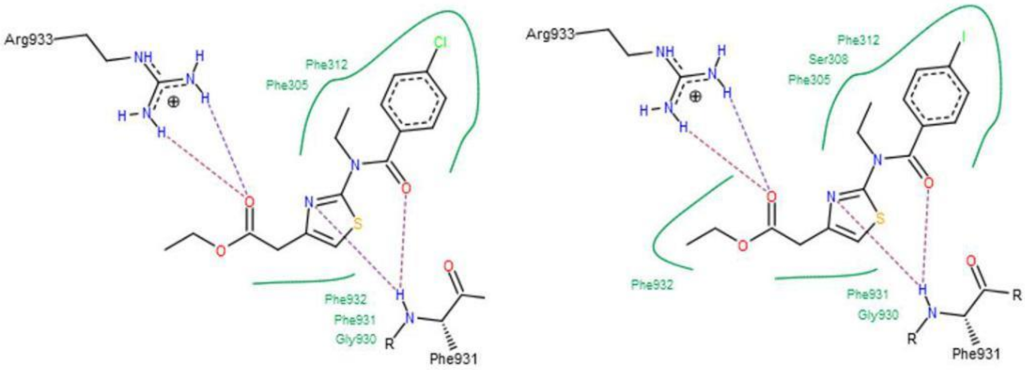

S44. Docking positioning of compound **100** (*C* atom; cyan) and **101** (*C* atom; green) at the CFTR protein in presence of VX-770 (*C* atom; magenta).

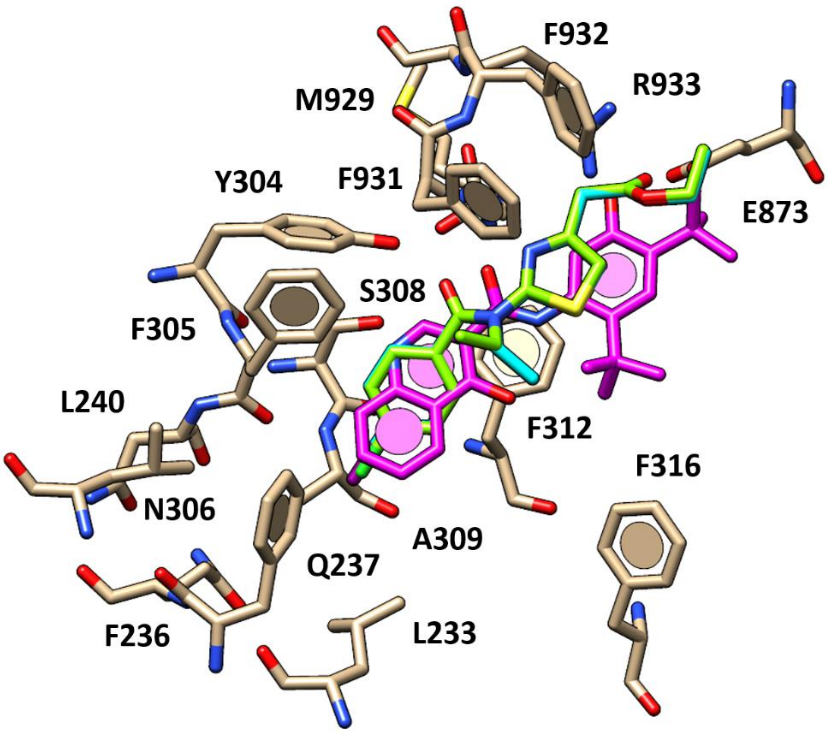

S45. Docking mode of **127** (*C* atom; green) within the CFTR cavity compared to that of VX-770 (*C* atom; magenta).

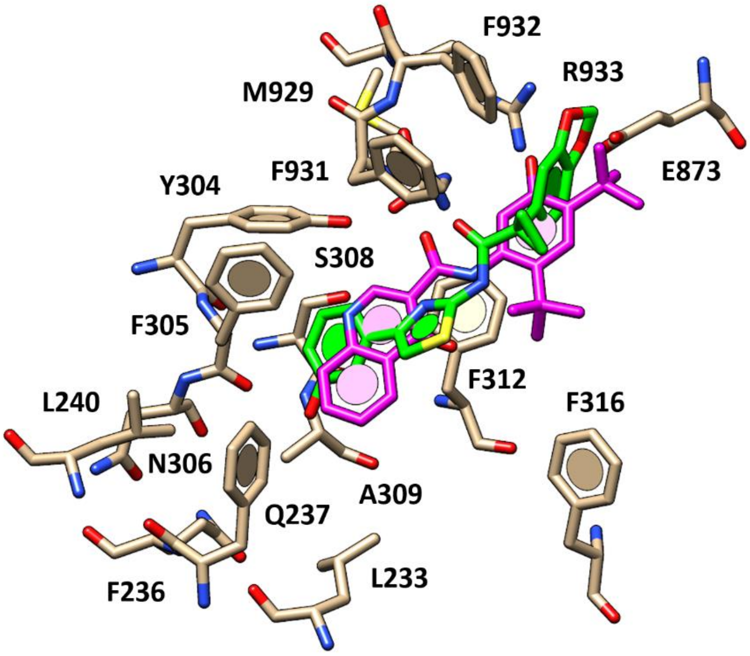

S46. Docking positioning of compound **139** (*C* atom; yellow) compared to that of VX-770 (*C* atom; magenta).

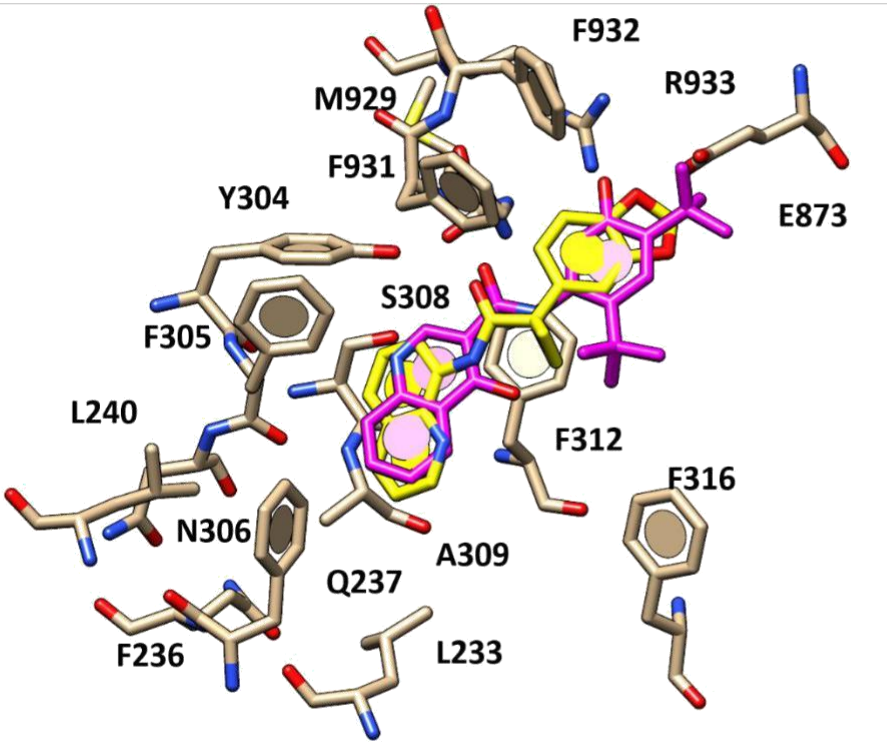

S47. Comparison of the thiazole **100** (*left*; *C* atom in coral) and **127** (*right*; *C* atom in green) with the potentiator **11** (*C* atom; white), depicted as reference compound, within the developed pharmacophore model.

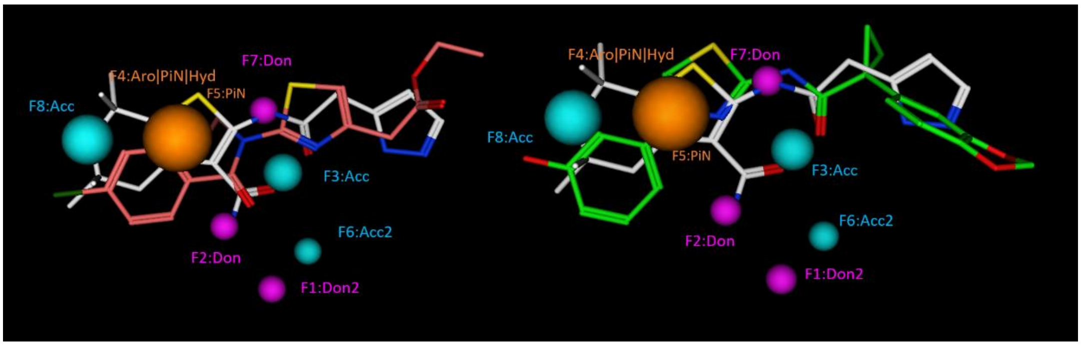

S48. Predicted pEC_{50} values of compounds **91**-**139** as putative F508del-CFTR potentiators, calculated by means of the developed QSAR model. Those compounds described in the literature as potentiators are shown in italic, blue colored. All the other compounds proved to be inactive.

Compound	Pred. pEC_{50}
**91**	3.76
**92**	4.18
**93**	4.35
**94**	3.56
**95**	2.82
**96**	3.44
**97**	3.47
**98**	2.65
**99**	4.04
**100**	5.02
101	5.21
102	4.33
**103**	3.11
**104**	3.77
**105**	3.74
**106**	2.96
**107**	4.06
**108**	4.02
**109**	4.04
**110**	4.09
**111**	3.96
**112**	4.07
**113**	4.09
**114**	4.23
**115**	4.24
**116**	3.89
**117**	4.16
**118**	3.99
**119**	3.48
**120**	3.24
**121**	3.49
**122**	3.72
**123**	4.58
**124**	3.82
**125**	3.97
**126**	4.02
127	4.87
128	5.31
**129**	3.27
**130**	3.90
**131**	4.23
**132**	3.00
**133**	3.00
**134**	3.62
**136**	4.02

**135**	4.17
**136**	3.66
**137**	4.09
**138**	3.57
139	4.72
